# Bayesian adaptive randomization in the I-SPY2 sequential multiple assignment randomized trial

**DOI:** 10.1093/biomtc/ujag063

**Published:** 2026-04-09

**Authors:** Peter Norwood, Christina Yau, Denise Wolf, Philip Beineke, Andrew Chapple, Anastasios Tsiatis, Marie Davidian

**Affiliations:** 1 Quantum Leap Healthcare Collaborative, San Francisco, CA 94104, United States; 2 Department of Surgery, University of California, San Francisco, San Francisco, CA 94143, United States; 3 Department of Statistics, North Carolina State University, Raleigh, NC 27695-8203, United States

**Keywords:** Bayesian response-adaptive randomization, breast cancer, precision oncology, sequential multiple assignment randomized trial

## Abstract

I-SPY2 is a long-running phase 2 platform trial that evaluates neoadjuvant treatments for locally advanced breast cancer to identify those with high efficacy that are likely to be successful in phase 3 trials, assigning patients to novel agents using response-adaptive randomization (RAR). Recently, I-SPY2 was reconfigured as a sequential multiple assignment randomized trial (SMART), with up to three stages of therapy. At the first stage, a patient is assigned to a tumor-subtype-specific therapy. If the patient fails to show a satisfactory response, the patient is assigned to a second subtype-specific therapy, and receives a third, rescue therapy if response is still not achieved. The I-SPY2 SMART thus supports identification of highly efficacious entire treatment regimes. The transition of I-SPY2 to a SMART required development of a RAR scheme that updates randomization probabilities at each stage, aligned with the goal of maximizing the number of patients who achieve a pathological complete response (pCR). We present our Bayesian RAR approach, which updates randomization probabilities based on the posterior probability that treatments are part of the optimal regime. Empirical studies demonstrate that it results in more patients having treatment experience consistent with highly efficacious regimes, improves overall within-trial pCR rates, and identifies optimal regimes post trial at rates similar to or exceeding those under simple, uniform, nonadaptive randomization.

## 1 Introduction

The I-SPY2 phase 2 trial in patients with stage II and III high-risk breast cancer is one of the first multicenter adaptive platform trials (Das and Lo, 2017) and has had a major impact on trial design and treatment. I-SPY2 is not a comparative trial; rather, it is characterized by its investigators as a signal-finding trial, focused on identifying investigational neoadjuvant agents targeted to biologically-defined patient subtypes that have a high probability to be successful in phase 3 trials (Nanda et al., 2020). Over more than a decade, I-SPY2 has identified multiple agents with strong efficacy signals based on the early endpoint of pathological complete response (pCR). Hallmarks of I-SPY2 are its use of response-adaptive randomization (RAR) (Atkinson and Biswas, 2013; Berry et al., 2010; Hu and Rosenberger, 2006) to preferentially assign patients to subtype-specific agents demonstrating efficacy and its role in establishing pCR as a strong predictor of event-free survival (I-SPY2 Trial Consortium, 2020) and markedly improving pCR rates in some subtypes. In some subtypes, standard of care, while highly efficacious (with 60-70% pCR rate), has evolved to include four or more targeted and/or cytotoxic agents in combination, inspiring a shift toward development of not only agents with better efficacy but also agents with similar efficacy and lower toxicity.

**Figure 1:**
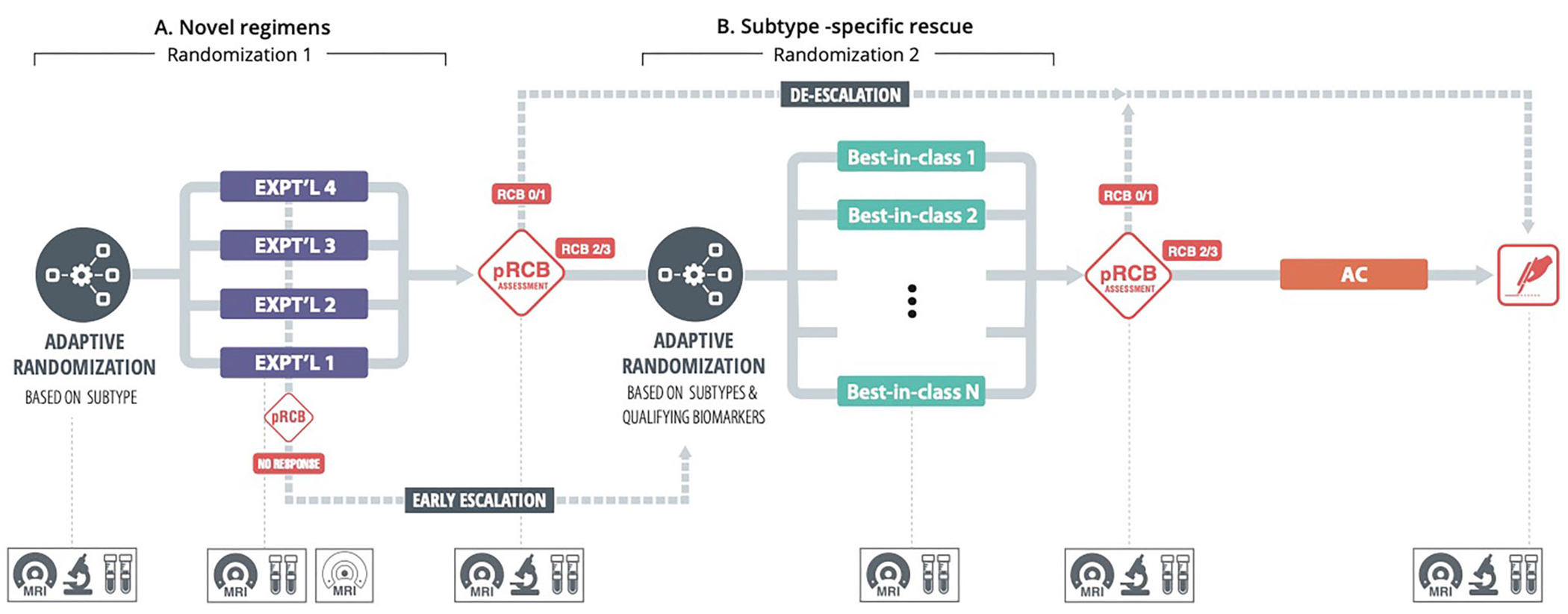
I-SPY2 SMART schema for a given subtype.

This interest in novel targeted agents with similar efficacy to current “best-in-class” agents but offering potential reduction in toxicities inspired a reconfiguration of I-SPY2 as a sequential multiple assignment randomized trial (SMART) (Murphy, 2005). A key goal is evaluation of new agents while giving participants likely to have a poor response to such agents the opportunity to switch to a best-in-class agent for their tumor subtype in a trial design mirroring clinical decision making in the course of patient care (Khoury et al., 2024; Shatsky et al., 2024). Patients classified as belonging to a given subtype follow the schema in Figure 1, involving two randomizations and three “blocks” of neoadjuvant therapy. Within a subtype, patients are initially randomized to subtype-specific novel experimental agents at stage 1 of the SMART, referred to by the investigators as “Block A.” During stage 1/Block A, a patient’s potential response to the assigned agent is evaluated every 3 to 6 weeks using an imaging biomarker, functional tumor volume (FTV), determined by breast MRI. At the completion of therapy (end of stage 1/Block A; about 12 weeks), biopsy of the tumor bed and, if needed, lymph nodes is performed, and an algorithm referred to as “preRCB” incorporating FTV and biopsy information is used to evaluate the patient’s probability of pCR (pCR can be confirmed only via surgical resection). Patients for whom the probability is sufficiently high, to whom we refer as “responders,” are given the option to proceed to surgery without further treatment (so meet the criteria for treatment de-escalation), at which time pCR status is ascertained. Those not meeting the criteria (“nonresponders”) are recommended to continue to stage 2 of the SMART, “Block B,” as are patients deemed earlier in Block A to be on a trajectory for poor response based on FTV (so are eligible for early treatment escalation), and are re-randomized to a subtype-specific best-in-class agent. Potential response is evaluated during stage 2/Block B via FTV, and at the end of stage 2/Block B, following biopsy, responders meeting the preRCB de-escalation criteria are given the option to proceed to surgery, and pCR status is determined. Nonresponders to Block B therapy as indicated by preRCB (or poor trajectory of earlier FTV) are recommended to receive rescue chemotherapy, at a minimum Adriamycin (doxorubicin) and cyclophosphamide, following which pCR status is ascertained. We refer to this further therapy for stage 2/Block B nonresponders as “stage 3,” although it is not a true stage of the SMART, as there is no randomization conducted.

Thus, a patient’s pCR outcome is ascertained at the end of stage 1, 2, or 3, depending on when she proceeds to surgery, and the likelihood patients receive unnecessary therapy and experience associated toxicities is minimized. Although we focus on this current design, our proposed methods can be generalized to more than two randomizations, which would support a true stage 3 with randomization among several rescue options (“Block C”).

As with any SMART, the I-SPY2 SMART design allows evaluation of entire treatment regimes (Lavori and Dawson, 2004; Tsiatis et al., 2020), which can be viewed as “algorithms” that dictate how therapies should be given to a patient sequentially. The randomization structure of a SMART induces a particular set of regimes, referred to as its embedded regimes; here, within a specific subtype, with subtype-specific sets of stage 1/Block A and stage 2/Block B treatment options, an example is “Give experimental therapy 1; if the patient does not proceed to surgery by the end of Block A, give best-in-class therapy 2 followed by rescue chemotherapy if the patient does not proceed to surgery by the end of Block B.” A key objective of the investigators is to contribute to the evolving the precision oncology knowledge base on how to use targeted therapies sequentially based on a patient’s response by identifying highly efficacious subtype-specific embedded regimes that may merit study in future trials, and especially the optimal regime(s) leading to the highest pCR rate.

Given the history of successful use of Bayesian RAR (bRAR) in I-SPY2, the investigators sought to implement such an RAR scheme in the SMART to update randomization probabilities at each stage to give greater weight to treatments that appear more efficacious based on data from previous patients. In conventional randomized controlled trials (RCTs), i.e., trials with a single point of randomization, the use of adaptive randomization has been argued to have the ethical benefit of improving within-trial outcomes (Legocki et al., 2015), and, while there can be a loss of efficiency of post-trial inference (Korn and Freidlin, 2011), this concern is less critical in multi-arm trials (Berry, 2011). However, RAR is less developed for SMARTs, which involve multiple randomizations, with only a few approaches available (Cheung et al., 2015; Norwood et al., 2024; Wang et al., 2022; Yang et al., 2024).

As the statistical team for I-SPY2, we were tasked to identify a bRAR approach that could be introduced straightforwardly when the trial transitioned to a two-stage SMART. Existing SMART RAR methods focus on certain designs, use ad hoc or non-Bayesian criteria for adaptation of randomization probabilities, or are too general to exploit the unique features of the I-SPY2 SMART. Accordingly, we developed a bRAR method tailored to I-SPY2. Aligned with bRAR methods used in conventional RCTs, particularly in oncology (Berry, 2015), our approach updates randomization probabilities at each stage of the SMART based on posterior probabilities, given the accrued data, that, within each subtype, each embedded regime is optimal in the sense of maximizing the probability of achieving pCR. Implementation is computationally straightforward, and the method is in use in the ongoing I-SPY2 SMART.

In this article, we present the I-SPY2 SMART bRAR strategy and demonstrate its performance. In Section 2, we review treatment regimes, SMARTS, and fundamentals of bRAR when used in conventional RCTs. We develop the statistical framework and bRAR scheme in Section 3. Section 4 presents results of simulation studies evaluating the approach.

## 2 Background

### 2.1 Treatment regimes and SMARTs

Formally, a treatment regime is a sequence of K decision rules corresponding to K decision points (stages) at which treatment decisions are to be made, where each rule takes a patient’s history of information to that point as input and outputs a recommended treatment option from among the available options. The SMART is the gold standard study design for evaluation of treatment regimes based on a given outcome. In a SMART, participants are randomized to the possible treatment options at each decision point/stage, and, as noted in Section 1, this structure induces the SMART’s embedded regimes. In the I-SPY2 SMART, for which K=2, for given subtype s,s=1,…,𝒮, stage 1 treatment option $a_{1}$ in the set of subtype-specific possible options ${𝒜}_{1,s}$, and stage 2 option $a_{2}$ in the set of subtype-specific possible options ${𝒜}_{2,s}$, a subtype s-specific embedded regime has stage 1 rule “Give $a_{1}$” and stage 2 rule “If the patient does not proceed to surgery during/after receiving $a_{1}$, give $a_{2}$ followed by rescue if the patient does not proceed to surgery during/after receiving $a_{2}$.”

There is a vast literature on estimating the expected outcome that would be achieved if the patient population were to follow the rules of a regime, referred to as the value of the regime; and on estimating an optimal regime, one that would lead to the most beneficial expected outcome/value if the population were to follow its rules, from suitable data. See Tsiatis et al. (2020) for a general account. In a SMART, interest often focuses on determining the optimal embedded regime. There may be more than one embedded regime achieving the most beneficial expected outcome and thus more than one optimal regime; for simplicity, we refer to “the” optimal embedded regime but recognize this possibility. Taking larger outcomes to be more beneficial, the optimal embedded regime is estimated as the regime among the embedded regimes for which the estimated value is largest. Thus, in the I-SPY2 SMART, with binary outcome pCR status, for each subtype, the optimal embedded regime is that maximizing the probability of pCR, and the estimated optimal embedded regime informs the investigators’ key goal of identifying regimes achieving high efficacy.

See Web Appendix A for a more extensive introduction to treatment regimes and SMARTs.

### 2.2 Bayesian response-adaptive randomization (bRAR)

In conventional RCTs; i.e., trials with a single point of randomization at which patients are to be randomized to treatment options within a set of possible options 𝒜, the standard approach to implementing bRAR is to base the randomization probability for each option a∈𝒜 when a new patient enters the trial on the posterior probability ρ(a∣𝒟) that a optimizes expected outcome among all options in 𝒜, where 𝒟 comprises the data accrued to that point (e.g., Wathen and Thall, 2017). Letting π(a∣𝒟) denote the randomization probability based on the accrued data, π(a∣𝒟) is usually linked to the posterior probability by a monotone function. A popular choice striking a balance between the posterior probability itself and simple, uniform (equal) randomization (Thall and Wathen, 2007) is to take

(1) $$\pi (a\;|\;𝒟)=\frac{\rho {\left(a\;|\;𝒟\right)}^{\psi }}{\sum_{a^{\prime }\in 𝒜}\rho {\left(a^{\prime }\;|\;𝒟\right)}^{\psi }},$$

where 0≤ψ≤1 is a damping contant such that ψ=1 corresponds to π(a∣𝒟)=ρ(a∣𝒟) and ψ=0 yields uniform randomization. As data accumulate, the randomization probability for an efficacious (inefficacious) treatment will increase (decrease) toward 1 (0), so that more (fewer) patients will receive it. Taking ψ equal to or close to 1 can result in aggressive adaptation and randomization probabilities approaching 1 or 0 for efficacious or inefficacious treatments, respectively, which can limit exploration of all options in 𝒜. Thus, an intermediate value for ψ strictly less than 1 may be a more practical choice. One can also take ψ to be time dependent to allow the aggressiveness to change with time.

The bRAR approach we propose in Section 3 for the I-SPY2 SMART is based on an intuitive extension of the foregoing principles. Within subtype s, randomization probabilities to the subtype-specific options in ${𝒜}_{1,s}$ for patients entering at stage 1 or to those in ${𝒜}_{2,s}$ for patients advancing to stage 2 are updated based on the posterior probability that an entire subtype s-specific embedded regime or stage 2 treatment option is optimal. Because randomization takes place within subtypes to subtype-specific treatment options, the I-SPY2 SMART can be viewed as comprising a set of subtype-specific “sub-SMARTS.” Accordingly, for brevity, in the formal framework in Section 3.1 and henceforth, we drop the subscript s with the understanding that the presentation refers to a single subtype. We note that a complementary view of the overall I-SPY2 SMART is as a covariate-adjusted response-adaptive (CARA) design (Rosenberger, 2025) in which randomization probabilities are skewed toward the best subtype-specific treatment options conditional on subtype.

A key concept in evaluation of treatment regimes is what is referred to as delayed effects, an interaction between treatment options across stages, so that an option selected at an earlier stage has implications for the options selected at subsequent stages. For example, the interaction may be antagonistic: a Block A agent may potentiate the effect of a Block B agent given to patients not proceeding to surgery after stage 1, so that the Block A option maximizing the probability of pCR after stage 1 may not be part of the regime leading to the maximum pCR rate overall. Accordingly, a SMART RAR scheme should determine stage 1 randomization probabilities to favor the stage 1 option that is part of the optimal regime. Our proposed bRAR approach, based on identifying optimal subtype-specific embedded regimes, incorporates this feature, in contrast to some approaches that base the stage 1 randomization probabilities on response status at the end of stage 1 (Wang et al., 2022; Yang et al., 2024).

## 3 Bayesian adaptive randomization in the I-SPY2 SMART

### 3.1 Statistical framework

As for conventional RCTs, we assume that patients enter I-SPY2 in a staggered fashion according to a completely random process over a planned accrual period, which we take for definiteness to be 30 months. Focusing on a specific subtype for which ${𝒜}_{1}$ is the set of stage 1 subtype-specific novel experimental therapies and ${𝒜}_{2}$ is the set of stage 2 subtype-specific best-in-class therapies, for a given patient of the subtype, let $A_{1}$ be the option in ${𝒜}_{1}$ to which the patient is randomized at entry. By about 12 weeks, whether or not a patient is a responder is determined. Ideally, all patients deemed responders would proceed to surgery; however, some may refuse, and nonresponders may choose to proceed to surgery even though not recommended to do so. Thus, aligned with an intention-to-treat perspective, we consider the regimes defined previously; namely, for $\left(a_{1},a_{2}\right)\in {𝒜}_{1}\;\times \;{𝒜}_{2}$, denote by $\left\{a_{1},a_{2}\right\}$ the subtype-specific embedded regime “Give $a_{1}$; if the patient does not proceed to surgery during/after receiving $a_{1}$, give $a_{2}$ followed by rescue therapy if the patient does not proceed to surgery during/after $a_{2}$.” Accordingly, let $R_{1}=1$ if the patient proceeds to surgery and 0 if not. If $R_{1}=1$, the patient’s true pCR status at the end of stage $1,Y_{1}$, is ascertained, where $Y_{1}=1(0)$ if the patient did (did not) achieve pCR, and no further data are collected on the patient. If $R_{1}=0$, the patient advances to stage 2 and is randomized to option $A_{2}$ in ${𝒜}_{2}$. By about 12 weeks, the patient’s response status is determined; if the patient proceeds to surgery, $R_{2}=1$, else $R_{2}=0$. If $R_{2}=1$, the patient’s true pCR status at the end of stage $2,Y_{2}\in \{0,1\}$, is ascertained, and no further data are collected. At both stages, there is a roughly 1 week delay between when $R_{1}=1$ and $R_{2}=1$ are observed and surgery/determination of $Y_{1}$ and $Y_{2}$. If $R_{2}=0$, the patient receives rescue therapy (“stage 3”), and after about 12 weeks true pCR status $Y_{3}\in \{0,1\}$ is ascertained. Thus, $Y_{k},k=1,2$, is observed only if $R_{k}=1$, and $Y_{3}$ is observed only if $R_{1}=R_{2}=0$, and the observed data on a patient are thus $𝒪=\left\{A_{1},R_{1},Y_{1}I\left(R_{1}=1\right),A_{2}I\left(R_{1}=0\right),R_{2}I\left(R_{1}=0\right),Y_{2}I\left(R_{1}=0,R_{2}=1\right),Y_{3}I\left(R_{1}=0,R_{2}=0\right)\right\}$. The primary outcome, recording whether or not pCR is achieved during the trial, is $Y=Y_{1}I\left(R_{1}=1\right)+Y_{2}I\left(R_{1}=0,R_{2}=1\right)+Y_{3}I\left(R_{1}=0,R_{2}=0\right)$, the pCR status ascertained when surgery takes place. A summary of all quantities is in Web Appendix B.

For a patient of the subtype of interest, define the following quantities. For $a_{1}\in {𝒜}_{1}$, let

(2) $$\theta_{1}\left(a_{1}\right)=P\left(R_{1}=1\;|\;A_{1}=a_{1}\right),\;\gamma_{1}\left(a_{1}\right)\;=P\left(Y_{1}=1\;|\;A_{1}=a_{1},R_{1}=1\right),$$

the conditional probability that a patient who receives treatment $a_{1}\in {𝒜}_{1}$ proceeds to surgery at stage 1 and the conditional probability that such a patient achieves pCR at stage 1, respectively. Similarly, for $a_{2}\in {𝒜}_{2}$, let

(3) $$\theta_{2}\left(a_{1},a_{2}\right)=P\left(R_{2}=1\;|\;A_{1}=a_{1},R_{1}=0,A_{2}=a_{2}\right),\;\gamma_{2}\left(a_{1},a_{2}\right)=P\left(Y_{2}=1\;|\;A_{1}=a_{1},R_{1}=0,A_{2}=a_{2},R_{2}=1\right),$$

the conditional probability that a patient who received $a_{1}\in {𝒜}_{1}$, did not respond, and received treatment $a_{2}\in {𝒜}_{2}$ proceeds to surgery at stage 2; and the conditional probability that such a patient achieves pCR at stage 2. Finally, the conditional probability that a patient who did not respond at stage 2 achieves pCR at stage 3 is

(4) $$\gamma_{3}\left(a_{1},a_{2}\right)=P\left(Y_{3}=1\;|\;A_{1}=a_{1},R_{1}=0,A_{2}=a_{2},R_{2}=0\right)$$

For regime $\left\{a_{1},a_{2}\right\}$, the probability of achieving pCR if the population of patients of this subtype were to follow regime $\left\{a_{1},a_{2}\right\}$, that is, the value of regime $\left\{a_{1},a_{2}\right\}$, is given by

(5) $$\mu \left(a_{1},a_{2}\right)=\theta_{1}\left(a_{1}\right)\gamma_{1}\left(a_{1}\right)+\left\{1-\theta_{1}\left(a_{1}\right)\right\}\theta_{2}\left(a_{1},a_{2}\right)\gamma_{2}\left(a_{1},a_{2}\right)\;+\left\{1-\theta_{1}\left(a_{1}\right)\right\}\left\{1-\theta_{2}\left(a_{1},a_{2}\right)\right\}\gamma_{3}\left(a_{1},a_{2}\right).$$

The expression (5) is intuitive and can be derived formally via the g-computation algorithm; e.g., see Tsiatis et al. (2020, Section 5.4). For convenience later, denote the entire set of probabilities involved in (5) for all possible options in ${𝒜}_{1}$ and ${𝒜}_{2}$ as $\Theta_{1}=\left\{\gamma_{1}\left(a_{1}\right),\gamma_{2}\left(a_{1},a_{2}\right),\gamma_{3}\left(a_{1},a_{2}\right),\theta_{1}\left(a_{1}\right),\theta_{2}\left(a_{1},a_{2}\right),\left(a_{1},a_{2}\right)\in {𝒜}_{1}\;\times \;{𝒜}_{2}\right.$.

From (5), the optimal subtype-specific embedded regime is $\left\{a_{1}^{\mathrm{opt}},a_{2}^{\mathrm{opt}}\right\}$ where $a_{1}^{\mathrm{opt}}$ and $a_{2}^{\mathrm{opt}}$ jointly maximize *μ*(*a*_1_, *a*_2_) in *a*_1_ and *a*_2_. It is straightforward to determine $a_{1}^{\mathrm{opt}}$ and $a_{2}^{\mathrm{opt}}$ by evaluating (5) at all of the (finite number of) possible combinations of options in ${𝒜}_{1}$ and ${𝒜}_{2}$. Note that $a_{1}^{\mathrm{opt}}$ and $a_{2}^{\mathrm{opt}}$ depend on $\Theta_{1}$. These developments suggest that, for a patient of this subtype entering the SMART at any time during the accrual period and requiring a stage 1 treatment assignment, the optimal strategy would be to assign the patient to $a_{1}^{\mathrm{opt}}$, the stage 1 option associated with regime $\left\{a_{1}^{\mathrm{opt}},a_{2}^{\mathrm{opt}}\right\}$ currently thought to be optimal and thus leading to the highest probability of achieving pCR during the trial.

Consider a patient already in the trial for whom *R*_1_ = 0, so who requires a stage 2 treatment assignment and who received option *a*_1_ at stage 1. Given that *a*_1_ has already been administered, analogous to the above, the optimal strategy at this point is to assign the patient to $a_{2}^{\mathrm{opt}}(a_{1})\;\in \;{𝒜}_{2}$, the option maximizing in *a*_2_ the probability of achieving pCR if the population were to receive this particular *a*_1_ at stage 1 and then *a*_2_ at stage 2, given by

(6) $$\mu_{2}(a_{1},a_{2})=\theta_{2}(a_{1},a_{2})\gamma_{2}(a_{1},a_{2})+\{1-\theta_{2}(a_{1},a_{2})\}\gamma_{3}(a_{1},a_{2})$$

It is easy to determine $a_{2}^{\mathrm{opt}}\left(a_{1}\right)$ by evaluating (6) at each option in ${𝒜}_{2}$. For given $a_{1}$, denote the set of probabilities in (6), which are a subset of those in $\Theta_{1}$, as $\Theta_{2}\left(a_{1}\right)=\left\{\gamma_{2}\left(a_{1},a_{2}\right),\gamma_{3}\left(a_{1},a_{2}\right),\theta_{2}\left(a_{1},a_{2}\right),a_{2}\in {𝒜}_{2}\right\}$. As above, $a_{2}^{\mathrm{opt}}\left(a_{1}\right)$ depends on $\Theta_{2}\left(a_{1}\right)$.

### 3.2 Adaptive randomization scheme

In principle, randomization probabilities can be updated each time a patient enters the trial at stage 1 or requires randomization to a stage 2 treatment. Logistically, it is practical to perform updates according to a schedule; for definiteness, assume that updates are done weekly, and let *t* index weeks since the start of enrollment. Then for patients who enter the SMART at stage 1 in the interval [*t*, *t* + 1) or who are already enrolled and reach stage 2 with *R*_1_ = 0 during [*t*, *t* + 1), the randomization probabilities for assigning stage 1 and stage 2 treatments updated at week *t* would be used to assign treatments for these patients.

Denote by ${𝒟}_{t}$ the data available at week t from previously-enrolled patients that can be used to update the randomization probabilities at week t. The following summary measures can be calculated based on ${𝒟}_{t}$ and used next to form relevant posterior probabilities. Because patients enter the trial in a staggered fashion, the data for a previously-enrolled patient in ${𝒟}_{t}$ will comprise only those variables already observed on the patient by week t, so that the patient will contribute only to the calculation of measures that involve only these variables.

Let *n*_1,*t*_(*a*_1_) denote the number of previously-enrolled patients of this subtype with *A*_1_ = *a*_1_ who have *R*_1_ observed before week *t*(so have completed stage 1 before *t*). Among these patients, let $R_{1,t}^{+}$ be the number with *R*_1_ = 1 before week *t*. Let $n_{1,t}^{*}(a_{1})$ denote the number of previously-enrolled patients of this subtype with *A*_1_ = *a*_1_ and *R*_1_ = 1 who have *Y*_1_ observed before week *t*, and let $Y_{1,t}^{+}$ be the number of these with *Y*_1_ = 1 before week *t*.

Let $n_{2,t}\left(a_{1},a_{2}\right)$ be the number of previously-enrolled patients of this subtype with $A_{1}=a_{1},R_{1}=0,A_{2}=a_{2}$ who have $R_{2}$ observed before week t (so have completed stage 2 before *t*), and among these let $R_{2,t}^{+}$ be the number with $R_{2}=1$. Let $n_{2,t}^{*}\left(a_{1},a_{2}\right)$ denote the number of previously-enrolled patients of this subtype with $A_{1}=a_{1},R_{1}=0,A_{2}=a_{2},R_{2}=1$ who have $Y_{2}$ observed before week t, and let $Y_{2,t}^{+}$ be the number of these with $Y_{2}=1$ before t.

Lastly, let $n_{3,t}\left(a_{1},a_{2}\right)$ be the number of previously-enrolled patients of this subtype with $A_{1}=a_{1},R_{1}=0,A_{2}=a_{2},R_{2}=0$ who have $Y_{3}$ observed (i.e., have completed “stage 3”) before week t, and let $Y_{3,t}^{+}\left(a_{1},a_{2}\right)$ the number among these for whom $Y_{3}=1$. For convenience, a summary of the foregoing definitions is given in Web Appendix B.

We now describe the proposed bRAR approach. For a new patient of this subtype entering the SMART during the interval [*t*, *t* + 1), the posterior probability that option $\ell \in {𝒜}_{1}$ is optimal in that it is associated with the optimal subtype-specific embedded regime is

(7) $$\rho_{1,t}\left(\ell \;|\;{𝒟}_{t}\right)=P\left\{a_{1}^{opt}\left({\text{Θ}}_{1}\right)=\ell \;|\;{𝒟}_{t}\right\},$$

where we make explicit the dependence of $a_{1}^{\mathrm{opt}}$ on $\Theta_{1}$. For a patient of the subtype who enrolled in the trial during week u<t and received stage 1 option $a_{1}\in {𝒜}_{1}$, reaches stage 2 during [t,t+1), and has $R_{1}=0$, given that the patient has already received $a_{1}\in {𝒜}_{1}$ at stage 1, the posterior probability at week t that option $k\in {𝒜}_{2}$ is optimal is

(8) $$\rho_{2,t}\left(k\;|\;a_{1},{𝒟}_{t}\right)=P\left[a_{2}^{opt}\left\{a_{1};\Theta_{2}\left(a_{1}\right)\right\}=k\;|\;{𝒟}_{t}\right],$$

which takes into account the additional data that have accrued since week *u*, where we make explicit the dependence of $a_{2}^{\mathrm{opt}}\left(a_{1}\right)$ on $\Theta_{2}\left(a_{1}\right)$.

From (7) and (8), for patients of this subtype, the posterior probabilities of the options in ${𝒜}_{1}$ and ${𝒜}_{2}$ being optimal at week t in the above sense depend on the posterior distribution at week t of $\Theta_{1}$, of which $\Theta_{2}\left(a_{1}\right)$ is a subset. To obtain this posterior distribution, we must specify a prior distribution for $\Theta_{1}$. A natural choice reflecting little knowledge of the probabilities in $\Theta_{1}$ is to take the prior for each component of $\Theta_{1}$ to be the Beta(1,1) distribution at all t, which is equivalent to a uniform distribution with support [0,1].

Under this specification, using the accrued data at week *t*, ${𝒟}_{t}$, and independent uniform priors for each component of $\Theta_{1}$, in obvious notation, the updated posterior distributions at week *t* for patients of this subtype can be obtained as

(9) $$\theta_{1}\left(a_{1}\right)\;|\;{𝒟}_{t}∼Beta\left\{1+R_{1,t}^{+}\left(a_{1}\right),1+n_{1,t}\left(a_{1}\right)-R_{1,t}^{+}\left(a_{1}\right)\right\}\;\gamma_{1}\left(a_{1}\right)\;|\;{𝒟}_{t}∼Beta\left\{1+Y_{1,t}^{+}\left(a_{1}\right),1+n_{1,t}^{*}\left(a_{1}\right)-Y_{1,t}^{+}\left(a_{1}\right)\right\}\;\theta_{2}\left(a_{1},a_{2}\right)\;|\;{𝒟}_{t}∼Beta\left\{1+R_{2,t}^{+}\left(a_{1},a_{2}\right),1+n_{2,t}\left(a_{1},a_{2}\right)-R_{2,t}^{+}\left(a_{1},a_{2}\right)\right\}\;\gamma_{2}\left(a_{1},a_{2}\right)\;|\;{𝒟}_{t}∼Beta\left\{1+Y_{2,t}^{+}\left(a_{1},a_{2}\right),1+n_{2,t}^{*}\left(a_{1},a_{2}\right)-Y_{2,t}^{+}\left(a_{1},a_{2}\right)\right\}\;\gamma_{3}\left(a_{1},a_{2}\right)\;|\;{𝒟}_{t}∼Beta\left\{1+Y_{3,t}^{+}\left(a_{1},a_{2}\right),1+n_{3,t}^{*}\left(a_{1},a_{2}\right)-Y_{3,t}^{+}\left(a_{1},a_{2}\right)\right\}.$$

Given that it is straightforward to draw random samples from the Beta distribution, we propose to approximate the posterior distributions (7) and (8) at week t by drawing a sample of size M, where M is large, from each posterior in (9) to obtain M random draws from the joint posterior of $\Theta_{1}$. Denoting the sample as $\left\{\Theta_{1}^{\left(m\right)},m=1,\ldots ,M\right\}$, approximate the posterior distribution $\rho_{1,t}\left(\ell \;|\;{𝒟}_{t}\right)$ in (7) that $\ell \in {𝒜}_{1}$ is optimal by

(10) $${\hat{\rho }}_{1,t}\left(\ell \;|\;{𝒟}_{t}\right)=M^{-1}\sum_{m=1}^{M}I\left\{a_{1}^{opt}\left(\Theta_{1}^{\left(m\right)}\right)=\ell \right\},$$

where I(⋅) is the indicator function. Note that obtaining (10) entails obtaining for each $m=1,\ldots ,Ma_{1}^{\mathrm{opt}}\left(\Theta_{1}^{\left(m\right)}\right)$ and $a_{2}^{\mathrm{opt}}\left(\Theta_{1}^{\left(m\right)}\right)$ jointly maximizing (5) in $a_{1}$ and $a_{2}$ with the components of $\Theta_{1}^{\left(m\right)}$ substituted. Given (10) for each $\ell \in {𝒜}_{1}$, the randomization probabilities to be used during [t,t+1) for each $a_{1}\in {𝒜}_{1}$ to assign stage 1 treatments to patients of this subtype entering the SMART during [t,t+1) are calculated, analogous to (1), as

(11) $$\pi_{1,t}\left(a_{1}\;|\;{𝒟}_{t};\psi_{t}\right)=\frac{{\hat{\rho }}_{1,t}{\left(a_{1}\;|\;{𝒟}_{t}\right)}^{\psi_{t}}}{\sum_{\ell \in {𝒜}_{1}}{\hat{\rho }}_{1,t}{\left(\ell \;|\;{𝒟}_{t}\right)}^{\psi_{t}}},$$

where we allow the damping constant $\psi_{t}$ to be week dependent.

Similarly, for each fixed $a_{1}\in {𝒜}_{1}$ and letting $\Theta_{2}^{\left(m\right)}\left(a_{1}\right)$ be the relevant subset of $\Theta_{1}^{\left(m\right)}$, the posterior distribution $\rho_{2,t}\left(k\;|\;{𝒟}_{t}\right)$ in (8) that $k\in {𝒜}_{2}$ is optimal can be approximated by

(12) $${\hat{\rho }}_{2,t}\left(k\;|\;a_{1},{𝒟}_{t}\right)=M^{-1}\sum_{m=1}^{M}I\left[a_{2}^{opt}\left\{a_{1},\Theta_{2}^{\left(m\right)}\left(a_{1}\right)\right\}=k\right].$$

As for (10), obtaining (12) requires obtaining for $m=1,\ldots ,Ma_{2}^{\mathrm{opt}}\left\{a_{1},\Theta_{2}^{\left(m\right)}\left(a_{1}\right)\right\}$ by maximizing (6) in $a_{2}$ with $a_{1}$ held fixed and the components of $\Theta_{2}^{\left(m\right)}\left(a_{1}\right)$ substituted. Given (12) for each $k\in {𝒜}_{2}$, the randomization probabilities to be used during [t,t+1) for each $a_{2}\in {𝒜}_{2}$ to assign stage 2 treatments to patients of this subtype already enrolled in the SMART of who received treatment $a_{1}$ in stage 1 and are nonresponders to $a_{1}$ (do not proceed to surgery) during [t,t+1) can be obtained for each $a_{1}\in {𝒜}_{1}$ as

(13) $$\pi_{2,t}\left(a_{2}\;|\;a_{1},{𝒟}_{t};\psi_{t}\right)=\frac{{\hat{\rho }}_{2,t}{\left(a_{2}\;|\;a_{1},{𝒟}_{t}\right)}^{\psi_{t}}}{\sum_{k\in {𝒜}_{2}}{\hat{\rho }}_{2,t}{\left(k\;|\;a_{1},{𝒟}_{t}\right)}^{\psi_{t}}}.$$

Cheung et al. (2015) and Norwood et al. (2024) propose RAR schemes for SMARTs based on the method of Q-learning for estimating an optimal regime (Tsiatis et al., 2020, Section 5.7.1), which here involves fitting a series of logistic regression models. That of Norwood et al. (2024) updates randomization probabilities based on a frequentist analog to the posterior distribution of the model parameters; that of Cheung et al. (2015) adapts the probabilities to favor treatments with large estimated models but does not account for uncertainty in model-fitting and requires specification of several tuning parameters. By comparison, our bRAR approach is ideally suited to the I-SPY2 SMART; e.g., if few patients reach stage 3 within a subtype, fitting frequentist logistic models for *γ*_3_ (*a*_1_, *a*_2_) could be problematic, a concern circumvented by sampling from the corresponding posterior in (9).

A concern with RAR is that an unusual configuration of early data could lead to adaptive randomization probabilities becoming “stuck” toward favoring suboptimal options (Thall et al., 2015). Thus, a “burn-in” period of uniform randomization is prudent so that sufficient patients can progress through all stages and provide data before adaptation is initiated. To ensure adequate exploration of all treatment options, it is also common to impose clipping constants, i.e., lower and upper bounds on the adaptive probabilities. A burn-in period and clipping are used in the I-SPY2 SMART. Choice of *M* should be based on preliminary investigation of stability of results.

### 3.3 Post-trial inference

As noted in Section 1, a goal in the I-SPY2 SMART is to identify highly efficacious subtype-specific embedded regimes. We thus require a suitable estimator for the value of each subtype-specific embedded regime $\left\{a_{1},a_{2}\right\}$, i.e., the pCR rate if all patients in the subtype population were to follow that regime, based on the final trial data ${𝒟}_{\mathrm{final}}$, for which pCR status is available for all patients. A natural approach is to draw a sample of size M from each posterior distribution in (9) to obtain draws $\Theta_{1}^{\left(m\right)},m=1,\ldots ,M$, from the joint posterior of $\Theta_{1}$ given ${𝒟}_{\mathrm{final}}$ and, for each regime $\left\{a_{1},a_{2}\right\}$, substitute these in (5) to obtain $\mu^{\left(m\right)}\left(a_{1},a_{2}\right),m=1,\ldots ,M$, which can be viewed as a sample from the posterior distribution of $\mu \left(a_{1},a_{2}\right)$. The Bayesian estimator for $\mu \left(a_{1},a_{2}\right),{\hat{\mu }}_{\mathrm{Bayes}}\left(a_{1},a_{2}\right)$, say, can then be obtained as the mode or mean of the sample, with the standard deviation of the sample as a measure of uncertainty. In the simulations of Section 4, because in our experience the distribution of the M samples is approximately symmetric and unimodal, we focus on the Bayesian estimator given by the mean of the M posterior draws, ${\hat{\mu }}_{\mathrm{Bayes}}\left(a_{1},a_{2}\right)=M^{-1}\sum_{m=1}^{M}\mu^{\left(m\right)}\left(a_{1},a_{2}\right)$.

Frequentist-type estimators are also possible. An intuitive plug-in estimator for $\mu \left(a_{1},a_{2}\right)$ can be obtained by estimating the quantities in (2)–(4) by functions of the obvious sample proportions in ${𝒟}_{\mathrm{final}}$, with approximate sampling distribution obtained via standard asymptotic normal theory; denote this estimator by ${\hat{\mu }}_{\mathrm{samp}}\left(a_{1},a_{2}\right)$. The theory assumes that the data in ${𝒟}_{\mathrm{final}}$ are independent and identically distributed (i.i.d.) across patients, which is not the case under RAR because the randomization probabilities are functions of previous data. Thus, the properties of ${\hat{\mu }}_{\mathrm{samp}}\left(a_{1},a_{2}\right)$ may not be well approximated by standard asymptotic theory, and inference on $\mu \left(a_{1},a_{2}\right)$ could be flawed (Zhang et al., 2021). Following Zhang et al. (2021), we construct an alternative estimator, denoted ${\hat{\mu }}_{\mathrm{wtsamp}}\left(a_{1},a_{2}\right)$, in which weighted sample proportions are used to estimate the quantities in (2)–(4), with the weights chosen so that asymptotic normality of ${\hat{\mu }}_{\mathrm{wtsamp}}\left(a_{1},a_{2}\right)$ can be established using the martingale central limit theorem, taking into account the dependence in ${𝒟}_{\mathrm{final}}$ induced by RAR. Formulations of ${\hat{\mu }}_{\mathrm{samp}}\left(a_{1},a_{2}\right)$ and ${\hat{\mu }}_{\mathrm{wtsamp}}\left(a_{1},a_{2}\right)$ are given in Web Appendix C.

To identify regimes with high efficacy as above, two post-trial analyses based on estimation of the values of subtype-specific embedded regimes are of interest in the I-SPY2 SMART. A key goal is to identify the optimal subtype-specific embedded regime, $\left\{a_{1}^{\mathrm{opt}},a_{2}^{\mathrm{opt}}\right\}$, with value $\mu \left(a_{1}^{\mathrm{opt}},a_{2}^{\mathrm{opt}}\right)$. The usual estimator is the regime with the largest estimated value (pCR rate) based on the chosen estimator for $\mu \left(a_{1},a_{2}\right)$, with $\mu \left(a_{1}^{\mathrm{opt}},a_{2}^{\mathrm{opt}}\right)$ estimated by the corresponding estimated pCR rate; see Web Appendix A. A second analysis seeks to identify regimes meriting further study by evaluating the probability that a regime’s value exceeds that implied by a predetermined, subtype-specific pCR rate distribution established based on previous (non-SMART) I-SPY2 data; regimes for which this probability is high are said to “graduate” from the trial. For brevity, we focus primarily on identifying the optimal regime in Section 4 and discuss graduation of regimes in Web Appendix E.

## 4 Simulation studies

We conducted a suite of simulation studies under a range of scenarios based on the I-SPY2 investigators’ expectations for experimental stage 1/Block A agents and past data on best-in-class stage 2/Block B agents and rescue therapy from I-SPY2. All scenarios involve a subtype for which there are two stage 1 options, ${𝒜}_{1}=\{0, 1\}$, and three stage 2 options, ${𝒜}_{2}=\{0, 1,2\}$. For each, we evaluate both in-trial performance and post-trial inference for SMARTs using simple, uniform randomization at each of stages 1 and 2, denoted as SR; and using the proposed bRAR strategy with $\psi_{t}=0.25, 0.50, 0.75, 1.00, 0.50\left(t/T_{end}\right),\left(t/T_{end}\right)$, denoted $BR\left(\psi_{t}\right)$, where *T_end_* is the last week randomization is required. The last two choices allow adaptation to become more aggressive over time. In all scenarios, clipping constants of 0.05 and 0.95 were imposed on the adaptive probabilities, and a burn-in period was implemented such that patients enrolling up until the week the 20th patient enrolled were randomized using SR at stages 1 and 2. All scenarios involved 5000 Monte Carlo trials under each randomization scheme, with *n* patients enrolled at weeks sampled uniformly from the integers in [1, *T_enroll_* = 130]. Here, *n* = 200, aligned with the sample size for the most prevalent subtype in I-SPY2. Full details of data generation are given in Web Appendix E.

A given scenario involves specification of the true pCR rates $p_{1}\left(a_{1}\right)=P\left(Y_{1}=1\;|\;A_{1}=a_{1}\right),\;a_{1}=0, 1$, following stage 1 treatment, and, given that pCR has not yet been achieved following stage 1 or stage 1 and 2 treatment, $p_{2}\left(a_{1},a_{2}\right)=P\left(Y_{2}=1\;|\;A_{1}=a_{1},Y_{1}=0,A_{2}=a_{2}\right)$ and $p_{3}\left(a_{1},a_{2}\right)=P\left(Y_{3}=1\;|\;A_{1}=a_{1},Y_{1}=0,A_{2}=a_{2},Y_{2}=0\right)$, $\left(a_{1},a_{2}\right)\in {𝒜}_{1}\;\times \;{𝒜}_{2}$; with sensitivity and specificity of the preRCB algorithm $\lambda_{\mathrm{sens}}=0.53\lambda_{\mathrm{spec}}=0.90$. It is shown in Web Appendix D that the foregoing quantities yield an expression for the true value $\mu \left(a_{1},a_{2}\right)$ of regime $\left\{a_{1},a_{2}\right\}$. Table 1 summarizes the true pCR rates used in each generative scenario and the implied values of the six embedded regimes.

We characterize in-trial performance under each scenario in Table 2. bRAR results in increased proportions of patients having in-trial treatment experience consistent with the optimal regime(s) relative to SR, with higher proportions associated with more aggressive adaptation; see Web Appendix A for discussion of the notion of a patient having experience consistent with a regime. The proportions consistent with the worst regime, with smallest true value, decrease relative to SR. These features are reflected in increases in the overall intrial pCR rate relative to that for SR. Because this pCR rate is averaged over results for all six embedded regimes and because bRAR with clipping constants implements exploration of all of them, higher probabilities of experience consistent with more efficacious regimes can translate at best to modest increases in overall pCR rate. Under bRAR, final randomization probabilities for the treatment options associated with the optimal regime(s) are larger than the nominal equal probabilities under SR, with larger probabilities resulting from more aggressive adaptation. Under Scenario 5, with two optimal regimes, proportions of patients with experience consistent with at least one of the optimal regimes are similar. Under null Scenario 0, with all regimes achieving the same value, there is no effect of bRAR, as expected.

**Figure 2:**
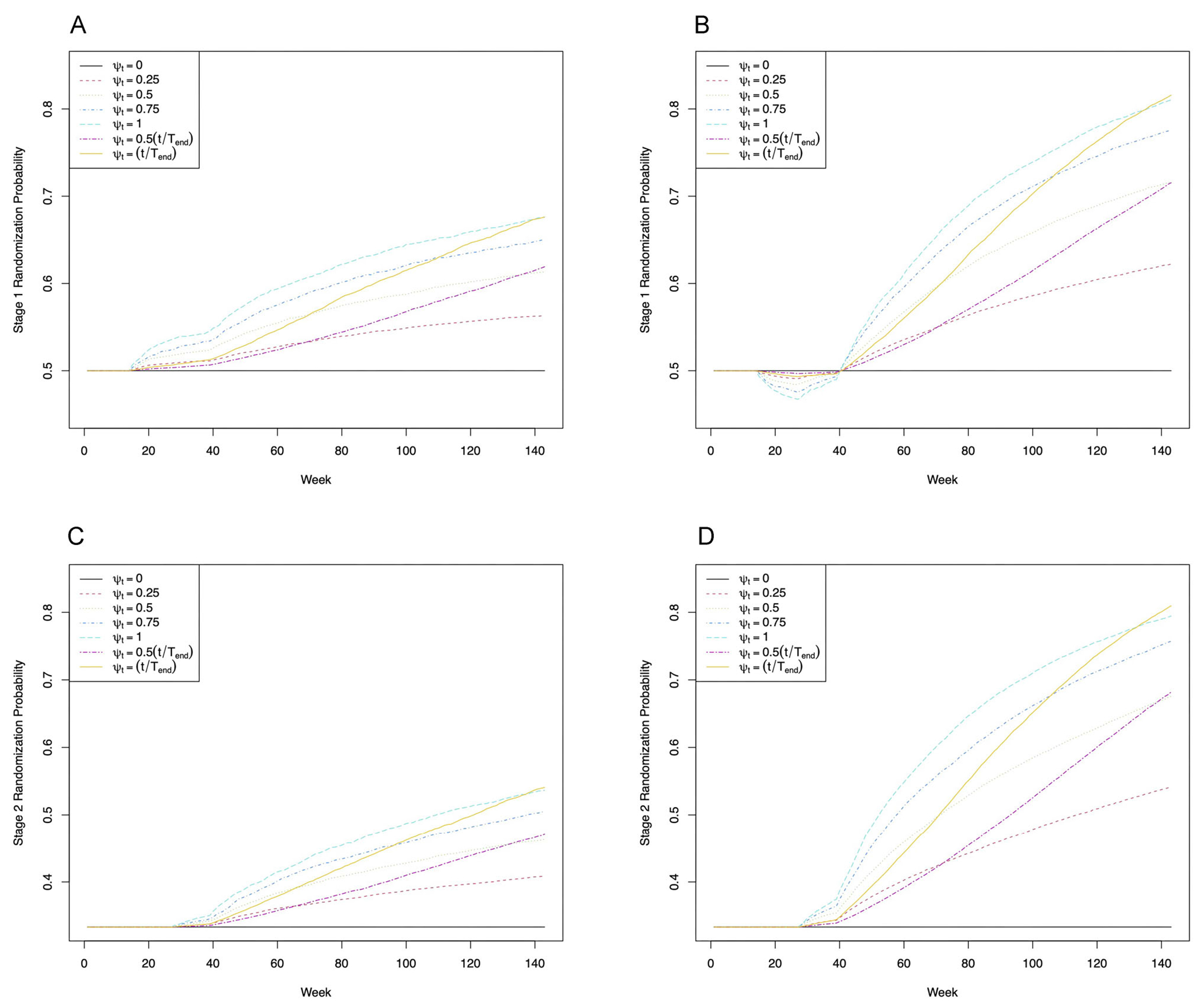
First row: Monte Carlo average randomization probabilities over the course of the trial to the stage 1 option associated with the optimal regime for (a) Scenario 1 and (b) Scenario 3 under each randomization scheme. Second row: Monte Carlo average randomization probabilities over the course of the trial to the stage 2 option associated with the optimal regime for patients randomized to the stage 1 option associated with the optimal regime under (c) Scenario 1 and (d) Scenario 4 for each randomization scheme.

The first row of Figure 2 presents Monte Carlo average randomization probabilities to the stage 1 option associated with the optimal regime. Because in antagonistic Scenario 3 option 0, that associated with the optimal regime, has lower pCR rate after stage 1 than option 1, following the burn-in, the probability of assignment to option 0 dips below 0.5 until sufficient information accrues at later stages to reflect its role in the optimal regime. Probabilities then increase, more dramatically so with more aggressive adaptive randomization. Under no delayed effects Scenario 1, option 1 has a higher pCR rate after stage 1 than option 0 and is associated with the optimal regime; thus, following the burn-in, randomization probabilities rise immediately to favor this option. The second row shows randomization probabilities to the stage 2 option associated with the optimal regime when preceded by the stage 1 option associated with the optimal regime, which rise as expected after the burn-in.

Table 3 summarizes post-trial inference results for Scenarios 1, 3, and 5, which address ability to identify the optimal regime(s) and quality of estimation of the value of a chosen, specific regime, focusing on that for the true optimal regime for definiteness. Results for Scenarios 2, 4, and 0 are presented in Web Appendix E. In all cases, the Bayesian estimator ${\hat{\mu }}_{\mathrm{Bayes}}\left(a_{1},a_{2}\right)$ was calculated using M=1000. Regardless of estimator, the proportion of trials for which the true optimal regime is identified increases under increasingly aggressive RAR relative to SR. All estimators exhibit some downward bias in estimating the value of the optimal regime(s) under all randomization schemes; the exception is ${\hat{\mu }}_{\mathrm{samp}}\left(a_{1},a_{2}\right)$ under SR, where standard asymptotic theory holds. The weighted estimator ${\hat{\mu }}_{\mathrm{wtsamp}}\left(a_{1},a_{2}\right)$ shows the smallest bias overall. In Web Appendix E, we present visual evidence of the nature of the bias of the first two estimators and of the effect of the weighting used in ${\hat{\mu }}_{\mathrm{wtsamp}}\left(a_{1},a_{2}\right)$ to reduce it. Monte Carlo coverage probabilities of confidence intervals in most cases are close to the nominal 0.95 level, with lower coverage in many cases associated with more aggressive adaptive randomization. Overall, length of confidence intervals based on ${\hat{\mu }}_{\mathrm{Bayes}}\left(a_{1},a_{2}\right)$ is shorter than that of those based on the frequentist estimators (and was shorter 80-95% of the time across scenarios). Under Scenarios 2-5, involving delayed effects, the weighted estimator is most efficient and achieves only slightly less precision than ${\hat{\mu }}_{\mathrm{Bayes}}\left(a_{1},a_{2}\right)$ under Scenario 1, with no delayed effects. Under all scenarios, with SR, not surprisingly, the Bayesian estimator yields improved precision over the sample proportion estimator. For Scenario 5, with two regimes achieving the optimal value, both ${\hat{\mu }}_{\mathrm{Bayes}}\left(a_{1},a_{2}\right)$ and ${\hat{\mu }}_{\mathrm{wtsamp}}\left(a_{1},a_{2}\right)$ show satisfactory performance; results for Scenario 0 in Web Appendix E support the use of ${\hat{\mu }}_{\mathrm{Bayes}}\left(a_{1},a_{2}\right)$ for post-trial inference when several regimes are expected to be optimal or close to optimal. See Web Appendix E for further discussion.

As noted in Section 1, a concern in conventional RCTs is loss of efficiency of post-trial inference on treatment effects under RAR relative to SR. In Web Appendix E, we present for each estimator Monte Carlo efficiency of the estimator under each bRAR scheme relative to under SR. Overall, the results suggest concern over efficiency loss for estimators of the value of a regime is not great and justified mainly for the most aggressive RAR schemes.

Although n=200 was chosen to reflect the conditions for I-SPY2, to gauge the effect of sample size on performance, we also present in Web Appendix E additional simulation results for Scenario 3 under n=120 and 1000. With n=1000, the downward bias exhibited by ${\hat{\mu }}_{\mathrm{Bayes}}\left(a_{1},a_{2}\right),{\hat{\mu }}_{\mathrm{samp}}\left(a_{1},a_{2}\right)$, and, to a lesser extent, ${\hat{\mu }}_{\mathrm{wtsamp}}\left(a_{1},a_{2}\right)$ decreases, with that of the latter negligible, suggesting that this feature is in part a finite sample issue.

The impact of possible temporal trends is an issue in any clinical trial and is well known to induce bias in inference on treatment effects from adaptively randomized trials. In Web Appendix E we present simulation results for a modification of Scenario 3 under which the pCR rates in Table 1 change over time. Temporal trends have no effect on the ability to identify the optimal regime under bRAR relative to under simple randomization. Not surprisingly, however, the results show that the quality of inference on the value of the optimal regime is degraded relative to that in scenarios with no temporal trends in that coverage of 95% confidence intervals is considerably lower than the nominal level, analogous to experience with inference on treatment effects conventional RCTs with RAR.

In Web Appendix E, we present results of simulations of the performance of two criteria for graduation of regimes suggested by Khoury et al. (2024) and Shatsky et al. (2024) that are aligned with the definition at the end of Section 3.3. Both criteria exhibit low probabilities of mistakenly concluding a regime that offers no improvement in pCR rate over previous treatments should graduate and high probabilities of concluding that a regime achieving high efficacy should graduate. When there are temporal trends in pCR rates, probabilities of mistakenly concluding a non-efficacious regime should graduate are inflated,

Overall, our results suggest that, under the conditions expected in the I-SPY2 SMART, the proposed bRAR approach results in higher proportions of trial participants being exposed to more efficacious treatment strategies and modest improvements in overall pCR rate. bRAR also results in improved ability to identify the optimal regime using any of the estimators post trial, with the Bayesian and weighted estimators achieving reliable performance.

## 5 Discussion

We have reported on the Bayesian RAR strategy we developed for use in the I-SPY2 SMART, which is implemented in the ongoing trial. In the trial, the randomization probability updates are on a fixed time schedule as in Section 3.2, providing a standardized process for updating and review, an important aspect of which is to ensure that higher randomization probabilities are aligned with more efficacious regimes. Probabilities and associated data are reviewed by the unblinded data safety and monitoring board monthly as an additional safe-guard.

A goal of transitioning I-SPY2 to a SMART was to provide participants multiple opportunities to achieve pCR while receiving the minimum amount of therapy needed to do so. Our simulations show that, under bRAR, more patients will have experience consistent with more efficacious regimes, aligning with the investigators’ objective of giving trial participants the greatest chance of achieving pCR while not compromising identification of efficacious regimes.

As noted in Section 3.1, we consider regimes with an intention-to-treat interpretation owing to possible noncompliance of patients with the preRCB recommendation on whether or not to proceed to surgery. Because noncompliance is not substantial in the I-SPY2 SMART, to mitigate simulation complexity, our generative scenarios do not incorporate a noncompliance mechanism. Accordingly, our results may be optimistic if noncompliance is considerable.

Our results show that, analogous to experience in conventional RCTs, temporal trends in pCR rates can lead to optimistic post-trial inference. It is possible to extend the methodology to incorporate discrete baseline covariates that could possibly be used in final analyses to account for temporal effects without significantly increasing the complexity of the methods. Such methodology will be fully developed and deployed for post-trial analyses if there is strong evidence of temporal trends.

Although we developed the proposed bRAR methods for I-SPY2, our approach can be translated straightforwardly to other SMART settings, as can the associated value estimators for the embedded regimes, and we expect that similar benefits would result.

## Supplementary Material

Web Appendices Web Appendices A–E referenced in Sections 2, 3, and 4 and code to implement the simulations are available with this paper at the Biometrics website on Oxford Academic.

Code for Simulations

Supplementary material is available at *Biometrics* online.

## Figures and Tables

**Table 1:** True pCR probabilities, stages 1 and 2, and true values of regimes, Scenarios 0–5. Scenario 1 involves no delayed effects of stage 1 treatments. Scenario 2 involves purely delayed effects of stage 1 treatments, which have identical probabilities of pCR. Scenarios 3 and 4 involve delayed effects: Scenario 3 involves antagonism, with the stage 1 option maximizing probability of pCR at stage 1 not associated with the optimal regime, and Scenario 4 involves synergy, with the stage 1 option with lower probability of pCR at stage 1 potentiating stage 2 option 0 to have a higher probability of pCR. Scenario 5 is similar to Scenario 3 but with two optimal regimes. Scenario 0 is an extreme “null” situation where all regimes have the same pCR rate. In all scenarios, $p_{3}\left(a_{1},a_{2}\right)=0.15$ for all $\left(a_{1},a_{2}\right)\in {𝒜}_{1}\;\times \;{𝒜}_{2}$. Optimal regime for each scenario is in boldface.

			Scenario					
Probability of pCR	*a*_1_	*a*_2_	1	2	3	4	5	0 (Null)
*p*_1_(*a*_1_)	0	–	0.30	0.30	0.30	0.30	0.30	0.30
	1	–	0.40	0.30	0.40	0.40	0.40	0.30
*p*_2_(*a*_1_, *a*_2_)	0	0	0.40	0.40	0.60	0.60	0.50	0.25
	0	1	0.30	0.30	0.50	0.25	0.50	0.25
	0	2	0.15	0.15	0.30	0.20	0.30	0.25
	1	0	0.40	0.40	0.18	0.30	0.18	0.25
	1	1	0.30	0.60	0.15	0.25	0.15	0.25
	1	2	0.15	0.25	0.10	0.20	0.10	0.25
*μ*(*a*_1_, *a*_2_)	0	0	0.603	0.603	**0.712**	**0.712**	**0.658**	0.521
	0	1	0.549	0.549	0.658	0.521	**0.658**	0.521
	0	2	0.467	0.467	0.549	0.494	0.549	0.521
	1	0	**0.660**	0.603	0.557	0.613	0.557	0.521
	1	1	0.613	**0.712**	0.543	0.590	0.543	0.521
	1	2	0.541	0.521	0.520	0.566	0.520	0.521

**Table 2:** In-trial results, Scenarios 0-5, *n* = 200. Overall pCR Rate is the Monte Carlo average pCR rate over all patients in the trial; Consist w/Opt is Monte Carlo average proportion of patients with experience consistent with the optimal regime(s); Consist w/Worst is Monte Carlo average proportion of patients with experience consistent with the worst regime; Rand Prob *a*_1_ Opt is the Monte Carlo average final updated randomization probability for the stage 1 option *a*_1_ associated with the optimal regime; Rand Prob *a*_2_ Opt is the Monte Carlo average final updated randomization probability for the stage 2 option *a*_2_ associated with the optimal regime(s) for patients randomized to the optimal stage 1 option. BR(·) is defined in the text. For Scenario 5, Consist w/Opt and Rand Prob *a*_2_ is shown for both optimal regimes {0, 0} and {0, 1}; for Scenario 0, Consist w/Opt and Rand Prob *a*_2_ is shown only for regime {0, 0} for brevity and was similar for all other regimes. Maximum Monte Carlo standard error of entries across scenarios and randomization schemes followed by the Monte Carlo standard error for SR (for comparison) is given in parentheses for each measure.

Measure	Scenario	SR	BR(0.25)	BR(0.5)	BR(0.75)	BR(1)	BR(0.5*t*/*T_end_*)	BR(*t*/*T_end_*)
Overall pCR Rate (0.0006,0.0005)	1	0.572	0.580	0.585	0.590	0.592	0.582	0.587
	2	0.575	0.588	0.600	0.606	0.610	0.593	0.604
	3	0.590	0.600	0.609	0.613	0.618	0.604	0.613
	4	0.582	0.594	0.603	0.609	0.614	0.598	0.607
	5	0.580	0.587	0.591	0.595	0.596	0.587	0.593
	0	0.520	0.522	0.522	0.521	0.522	0.521	0.521
Consist w/Opt (0.0020,0.0004)	1	0.258	0.288	0.314	0.335	0.354	0.299	0.329
	2	0.243	0.291	0.338	0.374	0.396	0.310	0.360
	3	0.244	0.286	0.330	0.359	0.377	0.305	0.351
	4	0.244	0.286	0.321	0.350	0.365	0.304	0.343
	5	0.243	0.269	0.292	0.308	0.319	0.279	0.303
		0.243	0.268	0.291	0.309	0.322	0.280	0.306
	0	0.243	0.243	0.244	0.244	0.242	0.244	0.243
Consist w/Worst (0.0011,0.0004)	1	0.243	0.212	0.191	0.176	0.167	0.205	0.184
	2	0.243	0.210	0.186	0.172	0.163	0.201	0.179
	3	0.257	0.230	0.208	0.193	0.185	0.222	0.197
	4	0.244	0.223	0.208	0.200	0.194	0.216	0.203
	5	0.257	0.237	0.218	0.206	0.196	0.228	0.209
	0	0.243	0.243	0.244	0.244	0.242	0.244	0.243
Rand Prob *a*_1_ Opt (0.0035, –)	1	0.500	0.563	0.613	0.650	0.676	0.619	0.676
	2	0.500	0.589	0.667	0.720	0.750	0.661	0.746
	3	0.500	0.622	0.716	0.776	0.810	0.715	0.816
	4	0.500	0.539	0.575	0.614	0.631	0.570	0.627
	5	0.500	0.591	0.666	0.716	0.631	0.664	0.752
	0	0.500	0.500	0.500	0.505	0.497	0.501	0.499
Rand Prob *a*_2_ Opt (0.0040, –)	1	0.333	0.409	0.463	0.505	0.536	0.471	0.541
	2	0.333	0.485	0.598	0.675	0.751	0.593	0.715
	3	0.333	0.437	0.519	0.568	0.598	0.513	0.597
	4	0.333	0.542	0.676	0.757	0.794	0.682	0.810
	5	0.333	0.380	0.410	0.425	0.431	0.405	0.436
		0.333	0.378	0.404	0.423	0.444	0.412	0.441
	0	0.333	0.333	0.333	0.335	0.334	0.333	0.335

**Table 3:** Post-trial results, n=200, Scenarios 1, 3, and 5 (Scenarios 2, 4, and 0 are in Web Appendix E). For each estimator, Prop Est Correct is the Monte Carlo proportion of trials for which the true optimal regime was correctly identified as optimal based on the given estimator; Est pCR Rate Opt Regime is the Monte Carlo average of estimates of the value of the true optimal regime; Coverage is the Monte Carlo coverage of a nominal 95% Wald confidence interval for $\mu \left(a_{1}^{\mathrm{opt}},a_{2}^{\mathrm{opt}}\right)$; Length is Monte Carlo average length of confidence intervals; and Rel Efficiency is the Monte Carlo relative efficiency of the indicated estimator to ${\hat{\mu }}_{\mathrm{Bayes}}\left(a_{1},a_{2}\right)$. For Scenario 5, results are shown for regime {0,0}; those for {0,1} are similar. Maximum Monte Carlo standard errors of Est pCR Rate Opt Regime and Length across scenarios 1-5 and 0 and randomization schemes are 0.0011 and 0.0010, respectively, for ${\hat{\mu }}_{\mathrm{Bayes}}\left(a_{1},a_{2}\right)$ (0.0010 and 0.0004 for SR); 0.0013 and 0.0012 for ${\hat{\mu }}_{\mathrm{samp}}\left(a_{1},a_{2}\right)$ (0.0010 and 0.0004 for SR); 0.0013 anSR); and 0.0013 and 0.0014 for ${\hat{\mu }}_{wtsamp}\left(a_{1},a_{2}\right)$ (0.0012 and-for SR). Maximum Monte Carlo standard errors for Coverage and Prop Est Correct across all entries are 0.0038 and 0.0071, respectively.

Estimator	Measure	SR	BR(0.25)	BR(0.5)	BR(0.75)	BR(1)	BR(0.5*t/T_end_*)	BR(*t/T_end_*)
		Scenario 1, $\mu (a_{1}^{\mathrm{opt}},a_{2}^{\mathrm{opt}})=0.660$						
${\hat{\mu }}_{\mathrm{Bayes}}\left(a_{1},a_{2}\right)$	Prop Est Correct	0.487	0.501	0.507	0.514	0.524	0.517	0.522
	Est pCR Rate Opt Regime	0.640	0.636	0.631	0.628	0.626	0.635	0.629
	Coverage	0.953	0.949	0.942	0.937	0.923	0.936	0.935
	Length	0.274	0.260	0.252	0.249	0.247	0.255	0.249
${\hat{\mu }}_{\mathrm{samp}}\left(a_{1},a_{2}\right)$	Prop Est Correct	0.483	0.502	0.506	0.511	0.518	0.519	0.519
	Est pCR Rate Opt Regime	0.659	0.651	0.644	0.639	0.635	0.649	0.640
	Coverage	0.939	0.939	0.937	0.929	0.927	0.935	0.933
	Length	0.292	0.277	0.269	0.266	0.265	0.272	0.265
	Rel Efficiency	0.825	0.898	0.906	0.884	0.861	0.902	0.891
${\hat{\mu }}_{\mathrm{wtsamp}}\left(a_{1},a_{2}\right)$	Prop Est Correct	–	0.502	0.506	0.511	0.518	0.519	0.519
	Est pCR Rate Opt Regime	–	0.656	0.651	0.648	0.646	0.655	0.649
	Coverage	–	0.940	0.939	0.934	0.931	0.934	0.935
	Length	–	0.277	0.270	0.270	0.270	0.272	0.267
	Rel Efficiency	–	0.921	0.969	0.960	0.949	0.933	0.968
		Scenario 3, $\mu (a_{1}^{\mathrm{opt}},a_{2}^{\mathrm{opt}})=0.712$						
${\hat{\mu }}_{\mathrm{Bayes}}\left(a_{1},a_{2}\right)$	Prop Est Correct	0.635	0.662	0.692	0.684	0.684	0.670	0.684
	Est pCR Rate Opt Regime	0.684	0.681	0.682	0.676	0.675	0.681	0.679
	Coverage	0.949	0.939	0.946	0.926	0.923	0.941	0.929
	Length	0.266	0.247	0.233	0.229	0.229	0.240	0.229
${\hat{\mu }}_{\mathrm{samp}}\left(a_{1},a_{2}\right)$	Prop Est Correct	0.648	0.668	0.694	0.688	0.684	0.678	0.694
	Est pCR Rate Opt Regime	0.712	0.704	0.701	0.692	0.690	0.702	0.696
	Coverage	0.930	0.937	0.945	0.935	0.931	0.943	0.933
	Length	0.279	0.258	0.243	0.240	0.241	0.251	0.240
	Rel Efficiency	0.913	1.028	1.069	1.013	1.006	1.059	1.047
${\hat{\mu }}_{\mathrm{wtsamp}}\left(a_{1},a_{2}\right)$	Prop Est Correct	–	0.668	0.694	0.688	0.684	0.678	0.694
	Est pCR Rate Opt Regime	–	0.707	0.707	0.701	0.700	0.707	0.704
	Coverage	–	0.936	0.943	0.941	0.937	0.939	0.935
	Length	–	0.257	0.244	0.243	0.245	0.251	0.242
	Rel Efficiency	–	1.059	1.144	1.135	1.136	1.112	1.146
		Scenario 5, $\mu (a_{1}^{\mathrm{opt}},a_{2}^{\mathrm{opt}})=0.658$						
${\hat{\mu }}_{\mathrm{Bayes}}\left(a_{1},a_{2}\right)$	Prop Est Correct	0.416	0.415	0.423	0.424	0.428	0.399	0.428
	Est pCR Rate Opt Regime	0.637	0.632	0.627	0.622	0.617	0.627	0.623
	Coverage	0.951	0.934	0.937	0.930	0.923	0.943	0.936
	Length	0.277	0.266	0.261	0.262	0.264	0.264	0.261
${\hat{\mu }}_{\mathrm{samp}}\left(a_{1},a_{2}\right)$	Prop Est Correct	0.416	0.423	0.432	0.432	0.429	0.406	0.432
	Est pCR Rate Opt Regime	0.658	0.649	0.642	0.635	0.628	0.643	0.637
	Coverage	0.934	0.941	0.935	0.931	0.927	0.941	0.937
	Length	0.295	0.283	0.279	0.282	0.285	0.281	0.280
	Rel Efficiency	0.837	0.914	0.918	0.900	0.860	0.946	0.918
${\hat{\mu }}_{\mathrm{wtsamp}}\left(a_{1},a_{2}\right)$	Prop Est Correct	–	0.423	0.432	0.432	0.429	0.406	0.432
	Est pCR Rate Opt Regime	–	0.654	0.650	0.645	0.640	0.649	0.646
	Coverage	–	0.938	0.937	0.931	0.931	0.942	0.939
	Length	–	0.282	0.280	0.285	0.291	0.281	0.282
	Rel Efficiency	–	0.939	0.980	0.988	0.943	0.983	0.999

## Data Availability

Data sharing is not applicable to this article, as no datasets are generated or analyzed.
