# Supplementary material for: Bayesian adaptive randomization in the I-SPY2 sequential multiple assignment randomized trial
Source: Biometrics. Author manuscript; Available in PMC 2026 May 12. (PMC13126647; doi:10.1093/biomtc/ujag063)
Supplement: Web Appendices — A–E referenced in Sections 2, 3, and 4 and code to implement the simulations are available with this paper at the Biometrics website on Oxford Academic. [file NIHMS2171016-supplement-Web_Appendices.pdf]

BIOMETRICS 000, 000–000 DOI: 000
000 0000

Supplementary Materials for "Bayesian adaptive randomization in the I-SPY2 sequential multiple assignment randomized trial"

Peter Norwood[1,*], Christina Yau[2], Denise Wolf[2], Philip Beineke[1], Andrew Chapple[1], Anastasios Tsiatis[3], and Marie Davidian[3]**

[1]Quantum Leap Healthcare Collaborative, San Francisco, CA 94158, United States

[2]Department of Surgery, University of California, San Francisco, San Francisco, CA 94143, United States

[3]Department of Statistics, North Carolina State University, Raleigh, NC 27695-8203, United States

*email: p.norwood@quantumleaphealth.org

**email: davidian@ncsu.edu

This paper has been submitted for consideration for publication in *Biometrics*

Web Appendix A. Fundamentals of treatment regimes and SMARTs

In this section, we provide a brief overview of fundamental definitions and concepts relevant to treatment regimes and sequential multiple assignment randomized trials (SMARTs).

Treatment regimes. As in the main paper, a treatment regime is a sequence of K decision rules corresponding to K decision points (stages) at which treatment decisions are to be made. Each rule takes a patient's history of information to that point as input and outputs a recommended treatment option from among the available options.

For example, generically, with $K = 2$, if there are two treatment options labeled 0 and 1 that could be given at the first decision point, and two treatment options that could be given at the second decision point labeled 2 and 3, an example of a treatment regime is "Give option 0 at the first decision point; if the patient meets the criteria for being a responder to that treatment at the second decision point, continue giving 0, otherwise, if the patient does not meet the criteria for being a responder at the second decision point, switch to option 2." Here, the decision rule at the first decision point (stage) is "Given option 0," and the decision rule at the second decision point (stage) is "If the patient is a responder, continue giving 0; otherwise, if the patient is not a responder, switch the patient to option 2." Note that this second decision rule takes into account the patient's history; namely, whether or not the patient responded to the treatment option s/he received at the first decision point. It is of course possible to define rules at the first decision point that take into account baseline patient information; for example, "Give option 0 if the patient is less than 50 years old; otherwise give option 1." The rules in the subtype-specific regimes of interest in the I-SPY2 SMART do not take account of additional such baseline information; thus, for simplicity in the following discussion, we focus on stage 1 rules that do not incorporate such information.

In the above example, we use different labels to indicate the treatment options at each stage for definiteness; however, one can use the same labels at different stages (keeping track

of what the labels mean at each stage). For example, in the simulations in Section 4 of the main paper, we use 0 and 1 to indicate two different stage 1/Block A experimental agents and 0, 1, and 2 to indicate three different stage2/Block B best-in-class agents (so that 0 and 1 at stage 1 represent entirely different treatment options from 0 and 1 at stage 2).

A regime can be thought of as an entire "algorithm" for treating an individual patient over K decision points. An individual patient can "follow" the entire algorithm defined by a regime to receive multiple treatments over a defined time frame in the course of his/her disease or disorder. The regime dictates not only the first treatment option the patient receives at the start of the time frame (first decision point/stage) but also subsequent treatments at later times/decision points/stages, where the algorithm takes account of the patient's past in dictating these treatments.

Given a set of decision points and the possible treatment options that could be given at each, one can identify several competing treatment regimes. In the simple example above, it is clear that there are four possible regimes the form "Give option a at the first decision point; if the patient meets the criteria for being a responder to that treatment at the second decision point, continue giving a, otherwise, if the patient does not meet the criteria for being a responder at the second decision point, switch to option b," where taking $(a, b) = (0, 2), (0, 3), (1, 2), (1, 3)$ yields the four regimes. We can summarize the four regimes as

1. Give option 0 at stage 1; if response, continue 0, otherwise if nonresponse, give option 2 at stage 2
2. Give option 0 at stage 1; if response, continue 0, otherwise if nonresponse, give option 3 at stage 2
3. Give option 1 at stage 1; if response, continue 1, otherwise if nonresponse, give option 2 at stage 2
4. Give option 1 at stage 1; if response, continue 1, otherwise if nonresponse, give option 3 at stage 2

Intuitively, given a health outcome of interest, just as we can envision the mean outcome if the entire patient population were to be given a particular treatment option at a particular (single) time point (e.g., at baseline in a usual clinical trial), we can envision the mean

outcome that would result if the entire patient population were to follow the algorithm dictated by a particular regime over the K decision points. The mean outcome if the entire population were to follow a given regime is referred to in the literature on treatment regimes as the *value* of the regime. Accordingly, just as it is standard to evaluate and compare different treatment options that could be given at a particular time point on the basis of the corresponding mean outcomes, different treatment regimes can be evaluated and compared based on the corresponding values.

Sequential multiple assignment randomized trials (SMARTs). In the main paper, we use the term "conventional randomized controlled trials (RCT)" to refer to "usual" clinical trials that focus on evaluating treatment options that could be given at a particular time point in a patient's disease/disorder; i.e., a single decision point. Such trials, with a single decision point at which participants are randomized to the treatment options under study, are a mainstay of clinical and biopharmaceutical research. Conventional RCTs base this evaluation on the mean outcomes as defined above or other measure, e.g., survival distributions, associated with each option studied; we focus on mean outcomes here given the focus of I-SPY2 on probability of pCR (mean of the binary variable "pCR status"). Randomization to the options at this single decision point allows us to view the samples of subjects assigned to receive each option as samples from the entire patient population if the population were given each of the options at this particular point in their disease or disorder.

It is natural to study entire regimes in an analogous way. The most common design for this purpose is the sequential multiple assignment randomized trial (SMART). In a SMART, instead of being randomized "up front" (at baseline) to follow each of the regimes, subjects are randomized at each decision point. For example, in the simple setting with $K = 2$ and $k = 4$ possible regimes above, the obvious SMART design would randomize subjects to treatment options 0 and 1 at decision point 1. At decision point 2, each subject's response

status would be ascertained. Subjects who are responders would continue on the option assigned at the first decision point; subjects who are not responders would undergo a second randomization to treatment options 2 or 3. The outcome would be ascertained on each at the specified follow up time. The following is a schematic of this design, where points of randomization are indicated by "R."

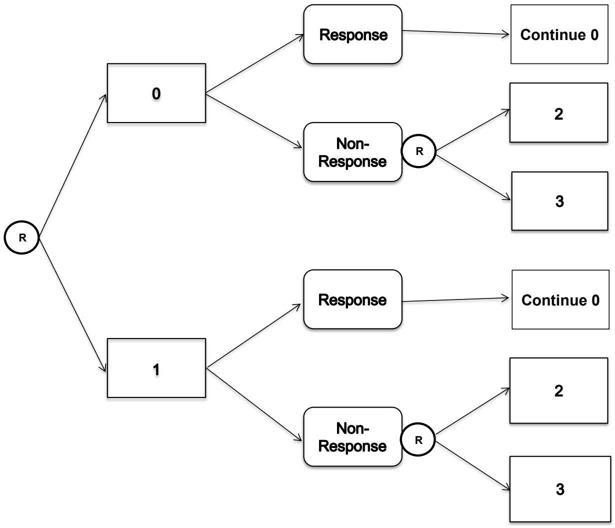

Embedded regimes and treatment experience consistent with following an embedded regime. From the above, in a SMART, subjects are not randomized to follow entire regimes. However, as noted in Section 1 of the main paper, the structure of the design of a SMART induces a set of treatment regimes, referred to as the embedded regimes. We now demonstrate this feature. For a given SMART, the design, involving sequential randomization, will lead to each participant having a "treatment experience" (also referred to in the literature as a "path" through the SMART). Consider the above example. We can identify all of the possible

treatment experiences (paths) that could a subject could have when participating in the SMART as follows:

a. Randomized at stage 1 to option 0, respond, continue option 0
b. Randomized at stage 1 to option 0, do not respond, randomized at stage 2 to option 2
c. Randomized at stage 1 to option 0, do not respond, randomized at stage 2 to option 3
d. Randomized at stage 1 to option 1, respond, continue option 1
e. Randomized at stage 1 to option 1, do not respond, randomized at stage 2 to option 2
f. Randomized at stage 1 to option 1, do not respond, randomized at stage 2 to option 3

It is critical to recognize that these treatment experiences (paths) are *not themselves regimes*, which is a common misconception. Rather, the possible treatment experiences that a subject could have in the trial are *consistent with* having followed one or more regimes in the sense that the treatment experience could have resulted from following the algorithm dictated by the rules of the regime.

To appreciate this concept, consider the four regimes 1–4 above and treatment experiences a–f. Note that experience a is consistent with having followed *either of* regimes 1 or 2; both regimes give treatment option 0 at stage 1 and dictate that in the event of response option 0 should continue. Likewise, experience d is consistent with having followed *either of* regimes 3 or 4; both regimes give treatment option 1 at stage 1 and dictate that in the event of response option 1 should continue. Experience b is consistent with having followed regime 1, which dictates that a patient who receives option 0 at stage 1 and does not respond should receive option 2 at stage 2; similarly, experience c is consistent with having followed regime 2, which dictates that a patient who receives option 0 at stage 1 and does not respond should receive option 3 at stage 2. Analogously, experience e is consistent with having followed regime 3, and f is consistent with having followed regime 4.

The result is that, in this SMART, there will be subjects whose realized treatment experi-

ence is consistent with having followed each of the four regimes above. Thus, it follows that it should be possible to evaluate all four regimes (e.g., estimate their values) based on data from the trial. The set of regimes for which there will be subjects with experience consistent with at least one of the regimes is identified as the set of *embedded regimes*.

Note that this demonstration implies that it is not possible to identify to which unique regime a subject was "randomized" in a SMART. Subjects who end up having experiences like a or d contribute information on *more than one regime*. Accordingly, the data from such subjects provides information on more than one regime. Standard methods for estimating the values of the embedded regimes in a SMART exploit this feature, so that such a subject contributes to the estimation of the values for both regimes.

Comparison of SMARTs and k-arm trials with "up-front" randomization to regimes. An alternative design to a SMART is a k-arm trial in which subjects are randomized "up front" to follow each of the regimes, with the outcome being ascertained on each at the specified follow up time. One might think at first glance that this design neatly categorizes subjects into mutually exclusive randomized "regime arms," where each arm dictates following a given regime. However, this view leads to inefficient use of the resulting data, owing to the considerations above. Namely, in the k-arm trial, as subjects follow their assigned regimes, some subjects will have treatment experience in the trial that is consistent with more than one of the regimes.

To see this, consider the example above. A subject who is randomized "up front" to follow regime 1 would receive option 0 at stage 1; if the subject responded, s/he would continue option 0 at stage 2, thus having experience a in the trial. As above, *despite the randomization*, this subject's experience is consistent with *both* regimes 1 and 2, and thus his/her data provides information on both of these regimes. Consequently, in practice, it is customary *not* to analyze the data from such a trial according to the randomization, so that

a subject randomized to regime 1 contributes data only to estimation of the value of regime 1, because this approach makes inefficient use of the data. Rather, the analysis is carried out using the same methods that are used to estimate the values of the embedded regimes in a SMART so as to gain efficiency by using all relevant data to estimate the value for each regime.

In fact, it is possible to design a RCT in which subjects are randomized once, "up front," to k regimes and a SMART embedding those k regimes, each with the same total number of subjects N, such that the designs are equivalent in the sense that the expected numbers of subjects receiving each treatment option at each decision point is the same. We illustrate with a simple example with $K = 2$ decision points. Suppose that there are two treatment options that could be given at the first decision point, labeled as 0 and 1, where 0 represents a "standard of care" option that a patient would continue receiving without modification until the outcome is ascertained. Suppose that there are two options that could be given at the second decision point, labeled as 2 and 3. Interest focuses on three treatment regimes:

1. Give option 0 at the first decision point and continue option 0 (so regardless of response status at the second decision point).
2. Give option 1 at the first decision point; if the patient responds, give option 2 at the second decision point, otherwise if the patient does not respond, give option 2 (thus, give option 2 regardless of response status)
3. Give option 1 at the first decision point; if the patient responds, give option 2 at the second decision point, otherwise if the patient does not respond, give option 3

The following is a schematic of a SMART embedding these three regimes:

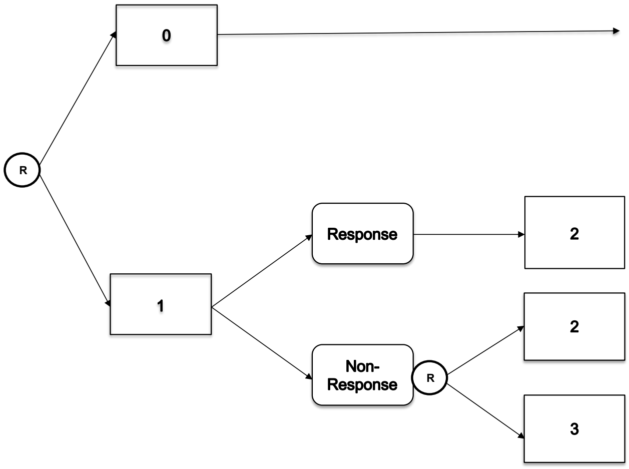

Suppose that $N = 300$ and that, in the patient population, 30% of patients will respond to stage 1 treatment 1 (and thus 70% will not respond). First consider a trial in which subjects are randomized "up front" to the 3 treatment regimes with equal probability (1/3). It is straightforward to deduce the following expected numbers of subjects:

- 100 subjects receive option 0 at stage 1 (randomized to regime 1)
- 200 subjects receive option 1 at stage 1 (randomized to regimes 2 or 3)
- $100 \times 0.3 = 30$ subjects randomized to regime 2 will respond (to stage 1 option 1)
- $100 \times 0.3 = 30$ subjects randomized to regime 3 will respond (to stage 1 option 1)
- $30 + 30 = 60$ subjects who received option 1 at stage 1 will respond and receive option 2 at stage 2
- $100 \times 0.7 = 70$ subjects randomized to regime 2 will not respond and receive option 2 at stage 2
- $100 \times 0.7 = 70$ subjects randomized to regime 3 will not respond and receive option 3 at stage 2

Summarizing, in stage 2, $60 + 70 = 130$ of the 200 subjects receiving option 1 at stage 1 will receive option 2 at stage 2, and 70 of the 200 subjects receiving option 1 at stage 1 will receive option 2 at stage 2.

Now consider the SMART in the schematic above. Suppose the $N = 300$ subjects are randomized at the first stage to option 0 with probability 1/3 and option 1 with probability 2/3. At stage 2, nonresponders to stage 1 option 1 are randomized with equal probability (1/2) to stage 2 options 2 and 3. We can deduce the following expected numbers of subjects:

- 100 subjects receive option 0 at stage 1 (randomized to option 0)
- 200 subjects receive option 1 at stage 1 (randomized to option 1)
- 200 x 0.3 = 60 subjects who received option 1 at stage 1 will respond and receive option 2 at stage 2
- 200 – 60 = 140 subjects who received option 1 at stage 1 will not respond
- 140/2 = 70 subjects who did not respond to option 1 receive option 2 at stage 2 (randomized to receive option 2)
- 140/2 = 70 subjects who did not respond to option 1 receive option 3 at stage 2 randomized to receive option 3)

Summarizing, in stage 2, 60 + 70 = 130 of the 200 subjects receiving option 1 at stage 1 will receive 2 at stage 2, and 70 of the 200 subjects receiving option at stage 1 will receive option 3 at stage 2. Comparing to the expected numbers of subjects in each condition to those for the 3-arm RCT where subjects are randomized "up front" to regimes shows that these numbers are identical. Accordingly, the two designs can be viewed as equivalent in this sense. It is possible to demonstrate this type of equivalence between any SMART embedding k regimes and a corresponding k-arm trial randomizing subjects to those regimes "up front."

An advantage of conducting a SMART rather than a k-arm trial randomizing subjects "up front" to regimes has to do with the ability to stratify randomization on key patient characteristics. In the k-arm trial, one can stratify randomization only at baseline (stage 1 treatment options); in a SMART, stratification can be conducted at both baseline (stage 1) and at stage 2. Namely, at stage 2 the stratification variable(s) can be both baseline charac-

teristics *and* characteristics that were ascertained during the intervening period between the first and second decision points.

Applying the above considerations to the I-SPY2 SMART, first note that in our simulation scenarios in Section 4 of the main paper and in Web Appendix E, there are two stage 1/Block A treatments labeled 0 and 1, and three stage2/Block B treatments labeled 0, 1, and 2. A participant who is assigned to treatment 0 at stage 1, is a responder, and proceeds to surgery at the end of stage 1 (at which point her pCR status is ascertained and she exits the trial) has treatment experience in the trial that is consistent with having followed *any* of regimes $\{0,0\}$, $\{0,1\}$, or $\{0,2\}$; each of these regimes starts a patient on treatment 0 and recommends that responders to treatment 0 proceed to surgery. Thus, as above, it is not possible to identify to which unique "regime" such a participant was "randomized." The three methods that we propose in the main paper for post-trial estimation the value of a regime all exploit the fact that a given subject's treatment experience may be consistent with having followed more than one regime.

There are numerous excellent articles on SMARTs that elaborate on all of the above points; e.g., Almirall et al. (2014).

Analysis. In practice, a common goal is to identify the optimal regime among those embedded in a SMART. In the I-SPY2 SMART, a key objective for each subtype is to identify the optimal subtype-specific embedded regime, which, as above, is that among all of the subtype-specific embedded regimes achieving the greatest pCR rate if the entire subtype population were to receive treatments according to the regime. As noted in the main paper, there may be more than one embedded regime achieving the same, most beneficial true expected outcome and thus more than one optimal regime; it is common for simplicity to refer to "the" optimal regime, recognizing that it may not be unique. Given the data from a SMART, and assuming that larger outcomes are more beneficial, the standard approach to estimating the optimal

embedded regime is to estimate the values for each of the embedded regimes, which can be accomplished using standard methods (Tsiatis et al., 2020, Chapters 5 and 6), and identify the optimal regime as that with the largest estimated value. In fact, this is the estimated optimal embedded regime one obtains by applying the method of Q-learning to estimation of the optimal embedded regime, a standard method for estimating an optimal regime (Tsiatis et al., 2020, Section 5.7.1). See below for further discussion of estimation of the values of each of the embedded regimes.

Most often in practice, identification of high-performing regimes in practice is generally regarded as an informal effort aimed at knowledge and hypothesis generation rather than as a confirmatory exercise. Accordingly, estimation of the optimal regime by inspecting the value estimates for the embedded regimes in a SMART as above, and more generally flagging certain regimes as highly efficacious on this basis and reporting the corresponding value estimates is a common practice.

In the main paper, for the I-SPY2 SMART, we have developed three estimators for the value of an embedded regime. As noted in the main paper, a key post-trial goal of the investigators is to estimate for each subtype the optimal subtype-specific embedded regime. In the trial, the optimal subtype-specific embedded regime is indeed estimated in the manner above, namely by estimating the values for each regime using one of these estimators and identifying the optimal regime as that with the largest estimated value. In summarizing post-trial performance in the simulations in Section 4 of the main paper and in Web Appendix E, for a given simulation scenario with 5000 Monte Carlo trials, under each of the randomization schemes considered, we present "the Monte Carlo proportion of trials for which the true optimal regime was correctly identified as optimal" based on each of the three estimators. The results suggest that basing this task on any of the three estimators leads to similar performance.

It is possible using standard methods for estimation of the values of the embedded regimes in a SMART and the associated asymptotic theory to carry out formal hypothesis tests comparing their true values; for example, a global test of the null hypothesis that all have the same value and tests focused on the null hypothesis that a specific pair or subset has the same value. In principle, one can carry out all possible pairwise comparisons among the values of the embedded regimes, controlling the family-wise error rate, in a more formal effort to identify the optimal regime, but this effort is typically so inefficient to be of little utility. Some methods have been developed to identify a set of the regimes that includes the optimal regime with at least some specified probability (e.g., Ertefaie et al., 2015). However, inference on the value of an optimal regime; that is, identification of which regime achieves the maximum value and construction of, say, a confidence interval for the value of the optimal regime so identified or a test for whether or not its value exceeds some threshold, is a challenging statistical inference problem. Namely, inference on the maximum is a nonstandard, or nonregular, statistical problem because the maximum of a set of means is a nonsmooth functional of the distribution of the data, so that standard asymptotic theory does not apply. See Chapter 10 of Tsiatis et al. (2020) for an introduction to this challenge and in particular the example in Section 10.2 on inference on the component-wise maximum of a mean vector, which illustrates the issues involved. Methods that do exist, for example, for constructing valid confidence sets, are typically so conservative as to be of little utility in actual practice.

Of course, estimation of the value of each embedded regime in a SMART is of interest independent of identification of the optimal regime. Estimation the value of a chosen, specific embedded regime, so of a fixed population parameter (the mean outcome if the population followed this specific regime), is a standard, or regular, statistical problem. Thus, methods for estimation of the value of a specific regime, such as those discussed by Tsiatis et al.

(2020, Chapters 5 and 6) cited above and those we have proposed, and associated confidence intervals for and tests regarding this fixed parameter, are valid approaches to inference on this parameter. In the simulations in Section 4 and Web Appendix E, we evaluate the quality of post-trial inference on the value of a chosen, specific regime by taking this regime for definiteness to be the true optimal regime determined by each simulation scenario. Of course, we could have presented summaries of the quality of post-trial inference on each of all six of the embedded regimes in each simulation scenario in the same way but for brevity limited to this one regime as representative of the performance overall. See Web Appendix E for discussion of simulation results for regimes other than the true optimal regime.

We conclude with a discussion of two of the measures we use to assess in-trial performance of our methodology. Specifically, in summarizing in-trial performance in Section 4 of the main paper and in Web Appendix E, for a given simulation scenario with 5000 Monte Carlo trials, under each of the randomization schemes considered, we present "the Monte Carlo average proportion of patients whose in-trial experience is consistent with having followed the optimal regime, with largest true pCR rate, and the worst regime, with the smallest." To obtain the first measure (for the optimal regime) for a given randomization scheme, we computed the proportion of patients (out of the n total) with experience consistent with the true optimal regime for each of the 5000 simulated trials. We then averaged these proportions over the 5000 trials; thus, this average reflects the true proportion of patients who would have experience consistent with the optimal regime under the given randomization scheme that would be expected in a trial like this involving n participants. To obtain the second measure (for the worst regime), we computed the proportion of patients (out of the n total) with experience consistent with the true worst regime for each of the 5000 simulated trials. We then averaged these proportions over the 5000 trials. Analogously, this average reflects the true proportion of patients who would have experience consistent with the worst regime

under the given randomization scheme that would be expected in a trial like this with n participants.

Web Appendix B. Summary of notation

For convenience, we summarize notation defined in the main paper here. As in the main paper, all notation refers to a single subtype of interest. Recall that $\mathcal{A}_1$ is the set of subtype-specific novel experimental therapy options to which patients of the subtype of interest can be randomized at stage 1, and elements of $\mathcal{A}_1$ are denoted as a_1. Likewise, $\mathcal{A}_2$ is the set of subtype-specific best-in-class therapy options to which patients of this subtype who do not proceed to surgery can be randomized at stage 2, and elements of $\mathcal{A}_2$ are denoted as a_2. With these definitions, the subtype-specific treatment regime

"Give a_1; if the patient does not proceed to surgery during/after receiving a_1, give a_2 followed by rescue therapy if the patient does not proceed to surgery during/after a_2"

is denoted for brevity as $\{a_1, a_2\}$ for all $(a_1, a_2) \in \mathcal{A}_1 \times \mathcal{A}_2$. As in Web Appendix A, the regimes $\{a_1, a_2\}$ for all $(a_1, a_2) \in \mathcal{A}_1 \times \mathcal{A}_2$ for patients of this subtype are referred to as the subtype-specific regimes embedded in the SMART.

Observed data on a given patient in the I-SPY2 SMART for an individual of the subtype of interest. The following table summarizes the variables that can be observed on a given patient, defined in Section 3.1 of the main paper.

Observed data on a given patient.

Symbol	Definition
Stage 1	
A_1	Option to which the patient is randomized at stage 1
R_1	Stage 1 response status, = 1 if the patient proceeds to surgery by the end of stage 1 and = 0 if not
Y_1	Indicator of pCR at the end of stage 1, = 1 if pCR achieved, = 1 if not; defined only if $R_1 = 1$
Stage 2: defined only for $R_1 = 0$	
A_2	Option to which the patient is randomized at stage 2
R_2	Stage 2 response status, = 1 if the patient proceeds to surgery by the end of stage 2 and = 0 if not
Y_2	Indicator of pCR at the end of stage 2, = 1 if pCR achieved, = 1 if not; defined only if $R_2 = 1$
Stage 3: defined only for $R_2 = 0$	
Y_3	Indicator of pCR at the end of stage 3, = 1 if pCR achieved, = 1 if not

Given these definitions, the observed data on a given patient, who may have her pCR status ascertained at any of stages 1, 2, or 3, can be expressed succinctly as

$$\mathcal{O} = \{A_1, R_1, Y_1 I(R_1 = 1), A_2 I(R_1 = 0), R_2 I(R_1 = 0), Y_2 I(R_1 = 0, R_2 = 1), Y_3 I(R_1 = 0, R_2 = 0)\},$$

and the outcome of interest, recording whether or not pCR is achieved during the trial, is determined by $\mathcal{O}$ as

$$Y = Y_1 I(R_1 = 1) + Y_2 I(R_1 = 0, R_2 = 1) + Y_3 I(R_1 = 0, R_2 = 0),$$

the pCR status ascertained when surgery takes place. Note that only one of Y_1, Y_2, Y_3 is observed on a given patient.

Probabilities characterizing the value of subtype-specific regime $\{a_1, a_2\}$. The following table summarizes the probabilities that characterize the value of regime $\{a_1, a_2\}$, denoted as $\mu(a_1, a_2)$, defined in Section 3.1 of the main paper; that is, the pCR rate that would be achieved if the population of patients of this subtype were to receive treatments according to the "algorithm" of regime $\{a_1, a_2\}$.

Probabilities characterizing the value $\mu(a_1, a_2)$ of subtype-specific regime $\{a_1, a_2\}$.

Symbol	Definition
Stage 1 (given in (2) of the main paper)	
$\theta_1(a_1)$	$P(R_1 = 1 \mid A_1 = a_1)$
$\gamma_1(a_1)$	$P(Y_1 = 1 \mid A_1 = a_1, R_1 = 1)$
Stage 2 (given in (3) of the main paper)	
$\theta_2(a_1, a_2)$	$P(R_2 = 1 \mid A_1 = a_1, R_1 = 0, A_2 = a_2)$
$\gamma_2(a_1, a_2)$	$P(Y_2 = 1 \mid A_1 = a_1, R_1 = 0, A_2 = a_2, R_2 = 1)$
Stage 3 (given in (4) of the main paper)	
$\gamma_3(a_1, a_2)$	$P(Y_3 = 1 \mid A_1 = a_1, R_1 = 0, A_2 = a_2, R_2 = 0)$

With these definitions, the value of regime $\{a_1, a_2\}$ is defined in (5) of the main paper and

is given by

$$\mu(a_1, a_2) = \theta_1(a_1)\gamma_1(a_1) + \{1 - \theta_1(a_1)\}\theta_2(a_1, a_2)\gamma_2(a_1, a_2) \\ + \{1 - \theta_1(a_1)\}\{1 - \theta_2(a_1, a_2)\}\gamma_3(a_1, a_2).$$

The (an) optimal regime is denoted as $\{a_1^{opt}, a_2^{opt}\}$, where a_1^{opt} and a_2^{opt} are the options in $\mathcal{A}_1$ and $\mathcal{A}_2$ that jointly maximize $\mu(a_1, a_2)$ over all $(a_1, a_2) \in \mathcal{A}_1 \times \mathcal{A}_2\}$.

The set of all probabilities in the table that determine the values of all embedded regimes, i.e., for all possible options (a_1, a_2) in $\mathcal{A}_1$ and $\mathcal{A}_2$ is denoted as

$$\Theta_1 = \{\gamma_1(a_1), \gamma_2(a_1, a_2), \gamma_3(a_1, a_2), \theta_1(a_1), \theta_2(a_1, a_2), \quad (a_1, a_2) \in \mathcal{A}_1 \times \mathcal{A}_2\}.$$

For a given, fixed $a_1 \in \mathcal{A}_1$, define the subset of probabilities in Θ_1

$$\Theta_2(a_1) = \{\gamma_2(a_1, a_2), \gamma_3(a_1, a_2), \theta_2(a_1, a_2), \quad a_2 \in \mathcal{A}_2\}.$$

Choice of prior distributions for components of Θ_1. As noted in the main paper, we take the prior for each of $\theta_1(a_1)$, $\gamma_1(a_1)$, $\theta_2(a_1, a_2)$, $\gamma_2(a_1, a_2)$, and $\gamma_3(a_1, a_2)$ for all a_1, a_2 at any time t to be the Beta$(1, 1)$ distribution at all t, which is equivalent to a Uniform(0,1) distribution. This choice reflects little knowledge of the values of the probabilities in Θ_1. We also considered a Beta$(0.5, 0.5)$ distribution, which relative to the Beta$(1, 1)$ modestly emphasizes smaller values, and found in simulations that qualitative results were entirely similar. As a frequentist alternative, we considered using so-called confidence distributions in place of posterior distributions (so eliminating the need to specify priors at all), in the same spirit as the approach taken by Norwood et al. (2024). Again, qualitative results were similar, with the frequentist approach having the downside of requiring fitting of logistic models based on very limited data early in simulated trials, leading to instability and in some cases failure of the adaptive randomization scheme. Accordingly, we have adopted

the Beta(1, 1) distribution as stated in the main paper for definiteness in evaluation of the proposed I-SPY2 SMART bRAR approach.

Quantities needed to characterize the posterior distributions of probabilities characterizing the value of subtype-specific regime $\{a_1, a_2\}$. In Section 3.2 of the main paper, $\mathcal{D}_t$ is defined as the data available at week t from patients previously enrolled in the SMART that can be used to update the randomization probabilities at week t. As noted there, because patients enter the trial in a staggered fashion, depending how far an already-enrolled patient has advanced by week t, only a subset of the information in $\mathcal{O}$ will be available on the patient.

The following table summarizes the definitions given in Section 3.2 of the main paper of the summary statistics that determine the updated posterior distributions of $\theta_1(a_1)$, $\gamma_1(a_1)$, $\theta_2(a_1, a_2)$, $\gamma_2(a_1, a_2)$, and $\gamma_3(a_1, a_2)$ at week t, given the accrued data $\mathcal{D}_t$ at week t, presented in (9) of the main paper.

Summary of notation, patients of the subtype of interest previously enrolled by week t.

Symbol	Definition
Stage 1	
$n_{1,t}(a_1)$	Number of patients from this subtype with $A_1 = a_1$ who have R_1 observed before week t
$R^+_{1,t}(a_1)$	Number of patients from this subtype with $A_1 = a_1$ who have $R_1 = 1$ before week t
$n^*_{1,t}(a_1)$	Number of patients from this subtype with $A_1 = a_1$, $R_1 = 1$ who have Y_1 observed before week t
$Y^+_{1,t}(a_1)$	Number of patients from this subtype with $A_1 = a_1$, $R_1 = 1$ who have $Y_1 = 1$ before week t
Stage 2 ($R_1 = 0$)	
$n_{2,t}(a_1, a_2)$	Number of patients from this subtype with $(A_1, A_2) = (a_1, a_2)$ who have R_2 observed before week t.
$R^+_{2,t}(a_1, a_2)$	Number of patients from this subtype with $(A_1, A_2) = (a_1, a_2)$ who have $R_2 = 1$ before week t
$n^*_{2,t}(a_1, a_2)$	Number of patients from this subtype with $(A_1, A_2) = (a_1, a_2)$, $R_2 = 1$ who have Y_2 observed before week t
$Y^+_{2,t}(a_1, a_2)$	Number of patients from this subtype with $(A_1, A_2) = (a_1, a_2)$, $R_2 = 1$ who have $Y_2 = 1$ before week t
Stage 3 ($R_1 = 0, R_2 = 0$)	
$n^*_{3,t}(a_1, a_2)$	Number of patients from this subtype with $(A_1, A_2) = (a_1, a_2)$ who have Y_3 observed before week t
$Y^+_{3,t}(a_1, a_2)$	Number of patients from this subtype with $(A_1, A_2) = (a_1, a_2)$ who have $Y_3 = 1$ before week t

Web Appendix C. Formulation of value estimators

Preliminaries. For convenience, we repeat the following definitions. For the subtype of interst and $a_1 \in \mathcal{A}_{1s}$ and $a_2 \in \mathcal{A}_{2s}$,

$$\theta_1(a_1) = P(R_1 = 1 \mid A_1 = a_1), \quad \gamma_1(a_1) = P(Y_1 = 1 \mid A_1 = a_1, R_1 = 1), \tag{C1}$$

$$\begin{aligned}\theta_2(a_1, a_2) &= P(R_2 = 1 \mid A_1 = a_1, R_1 = 0, A_2 = a_2),\\ \gamma_2(a_1,a_2) &= P(Y_2 = 1 \mid A_1 = a_1, R_1 = 0, A_2 = a_2, R_2 = 1).\end{aligned} \tag{C2}$$

and

$$\gamma_3(a_1, a_2) = P(Y_3 = 1 \mid A_1 = a_1, R_1 = 0, A_2 = a_2, R_2 = 0). \tag{C3}$$

Then the value of the subtype-specific regime $\{a_1, a_2\}$, i.e., the probability of achieving pCR if the population of patients of this subtype were to follow regime $\{a_1, a_2\}$, is

$$\begin{aligned}\mu(a_1, a_2) = \theta_1(a_1)\gamma_1(a_1) + \{1 - \theta_1(a_1)\}\theta_2(a_1, a_2)\gamma_2(a_1, a_2)\\ + \{1 - \theta_1(a_1)\}\{1 - \theta_2(a_1, a_2)\}\gamma_3(a_1, a_2).\end{aligned} \tag{C4}$$

Suppose that there are N patients across all subtypes, n of whom are from of the subtype of interest. Note that at any week t, as seen in Table 1 in the main paper, the part of the available data $\mathcal{D}_t$ on previously enrolled patients that are relevant to updating randomization probabilities for patients of this subtype at week t are the subsets of $\mathcal{O}$ that have already been observed before t on among the n patients who enrolled prior to t. Indexing these n patients by i, at the conclusion of the trial, the part of the final data $\mathcal{D}_{final}$ relevant to estimating $\mu(a_1, a_2)$ for this subtype are $\mathcal{O}_i$, $i = 1, \ldots, n$.

Bayesian estimator. As discussed in the main paper, the proposed Bayesian estimator $\widehat{\mu}_{Bayes}(a_1, a_2)$ is calculated based on these data. Namely, with

$$\Theta_1 = \{\gamma_1(a_1), \gamma_2(a_1, a_2), \gamma_3(a_1, a_2), \theta_1(a_1), \theta_2(a_1, a_2);\;\; (a_1, a_2) \in \mathcal{A}_1 \times \mathcal{A}_2\},$$

to estimate the value of the subtype-specific embedded regime $\{a_1, a_2\}$, draw a sample of size M from each of the posterior distributions given $\mathcal{D}_{final}$ of the components of Θ_1 to obtain draws $\Theta_1^{(m)}$, $m = 1, \ldots, M$, from the joint posterior of Θ_1 given $\mathcal{D}_{final}$. Then substitute each of these draws in (C4) to obtain $\mu^{(m)}(a_1, a_2)$, $m = 1, \ldots, M$, which can be viewed as a sample from the posterior distribution of $\mu(a_1, a_2)$ given $\mathcal{D}_{final}$. The Bayesian estimator

$\widehat{\mu}_{Bayes}(a_1, a_2)$ is obtained as the mean or mode of the sample, with the standard deviation of the sample as a measure of uncertainty.

Sample proportions estimator. We now present the formulation of the alternative estimators $\widehat{\mu}_{samp}(a_1, a_2)$ and $\widehat{\mu}_{wtsamp}(a_1, a_2)$ based on the final data $\mathcal{O}_i$, $i = 1, \ldots, n$ on the patients of the subtype of interest. These estimators may have appeal in that they can be considered as frequentist alternatives to $\widehat{\mu}_{Bayes}(a_1, a_2)$. Here, we consider $\widehat{\mu}_{samp}(a_1, a_2)$.

First, note from (C1)-(C3) that

$$\theta_1(a_1)\gamma_1(a_1) = \frac{\alpha_1(a_1)}{\alpha_2(a_1)} = \frac{P(A_1 = a_1, R_1 = 1, Y_1 = 1)}{P(A_1 = a_1)},$$

$$\theta_2(a_1, a_2)\gamma_2(a_1, a_2) = \frac{\alpha_3(a_1, a_2)}{\alpha_4(a_1, a_2)} = \frac{P(A_1 = a_1, R_1 = 0, A_2 = a_2, R_2 = 1, Y_2 = 1)}{P(A_1 = a_1, R_1 = 0, A_2 = a_2)},$$

$$\{1 - \theta_2(a_1, a_2)\}\gamma_2(a_1, a_2) = \frac{\alpha_5(a_1, a_2)}{\alpha_4(a_1, a_2)} = \frac{P(A_1 = a_1, R_1 = 0, A_2 = a_2, R_2 = 0, Y_3 = 1)}{P(A_1 = a_1, R_1 = 0, A_2 = a_2)},$$

$$\{1 - \theta_1(a_1)\} = \frac{\alpha_6(a_1)}{\alpha_2(a_1)} = \frac{P(A_1 = a_1, R_1 = 0)}{P(A_1 = a_1)}.$$

Thus, $\mu(a_1, a_2)$ also can be written as

$$\mu(a_1, a_2) = \frac{\alpha_1(a_1)}{\alpha_2(a_1)} + \frac{\alpha_6(a_1)}{\alpha_2(a_1)} \left\{ \frac{\alpha_3(a_1, a_2) + \alpha_5(a_1, a_2)}{\alpha_4(a_1, a_2)} \right\}. \tag{C5}$$

Given the representation in (C5), a natural plug-in estimator is based on substituting sample proportions for the probabilities $\alpha_1(a_1), \alpha_2(a_1), \alpha_3(a_1, a_2), \alpha_4(a_1, a_2), \alpha_5(a_1, a_2), \alpha_6(a_1)$, namely,

$$\widehat{\mu}_{samp}(a_1, a_2) = \frac{\widehat{\alpha}_1(a_1)}{\widehat{\alpha}_2(a_1)} + \frac{\widehat{\alpha}_6(a_1)}{\widehat{\alpha}_2(a_1)} \left\{ \frac{\widehat{\alpha}_3(a_1, a_2) + \widehat{\alpha}_5(a_1, a_2)}{\widehat{\alpha}_4(a_1, a_2)} \right\}, \tag{C6}$$

where

$$\widehat{\alpha}_1(a_1) = n^{-1} \sum_{i=1}^{n} I(A_{1i} = a_1, R_{1i} = 1, Y_{1i} = 1), \quad \widehat{\alpha}_2(a_1) = n^{-1} \sum_{i=1}^{n} I(A_{1i} = a_1),$$

$$\widehat{\alpha}_3(a_1, a_2) = n^{-1} \sum_{i=1}^{n} I(A_{1i} = a_1, R_{1i} = 0, A_{2i} = a_2, R_{2i} = 1, Y_{2i} = 1),$$

$$\widehat{\alpha}_4(a_1, a_2) = n^{-1} \sum_{i=1}^{n} I(A_{1i} = a_1, R_{1i} = 0, A_{2i} = a_2),$$

$$\widehat{\alpha}_5(a_1,a_2) = n^{-1}\sum_{i=1}^n I(A_{1i}=a_1, R_{1i}=0, A_{2i}=a_2, R_{2i}=0, Y_{3i}=1),$$

$$\widehat{\alpha}_6(a_1) = n^{-1}\sum_{i=1}^n I(A_{1i}=a_1, R_{1i}=0).$$

As discussed in Section 3.3 of the main paper, because of the use of adaptive randomization, the final data from patients of this subtype, $\mathcal{O}_i$, $i=1,\ldots,n$, are not independent and identically distributed (i.i.d.) over i, so that standard asymptotic theory may not apply to derive the properties of (C6). Thus, it is not even clear that the estimators above are consistent estimators for the corresponding probabilities. Nonetheless, proceeding naively as if the data are i.i.d. and applying standard theory, one can derive an approximate standard error for $\widehat{\mu}_{samp}(a_1,a_2)$ by finding the associated influence function. We provide a heuristic argument. Denote by $\alpha_1^*(a_1), \alpha_2^*(a_1), \alpha_3^*(a_1,a_2), \alpha_4^*(a_1,a_2), \alpha_5^*(a_1,a_2), \alpha_6^*(a_1)$ the limits in probability of $\widehat{\alpha}_1(a_1), \widehat{\alpha}_2(a_1), \widehat{\alpha}_3(a_1,a_2), \widehat{\alpha}_4(a_1,a_2), \widehat{\alpha}_5(a_1,a_2), \widehat{\alpha}_6(a_1)$, which would be the corresponding true probabilities if the data were i.i.d. Let

$$\mu^*(a_1,a_2) = \frac{\alpha_1^*(a_1)}{\alpha_2^*(a_1)} + \frac{\alpha_6^*(a_1)}{\alpha_2^*(a_1)}\left\{\frac{\alpha_3^*(a_1,a_2)+\alpha_5^*(a_1,a_2)}{\alpha_4^*(a_1,a_2)}\right\}.$$

We find the mean-zero quantity $\mathcal{I}_i$ such that

$$n^{1/2}\left\{\widehat{\mu}(a_1,a_2) - \mu^*(a_1,a_2)\right\} = n^{-1/2}\sum_{i=1}^n \mathcal{I}_i + o_p(1). \tag{C7}$$

As is well known, with i.i.d. data, it follows that the left hand side of (C7) converges in distribution to a normal random variable with mean zero and variance that can be approximated by $n^{-1}\sum_{i=1}^n \mathcal{I}_i^2$. Assuming that $n^{1/2}\{\widehat{\alpha}_j(a_1) - \alpha_j^*(a_1)\} = O_p(1)$, $j=1,2,6$, and $n^{1/2}\{\widehat{\alpha}_j(a_1,a_2) - \alpha_j^*(a_1,a_2)\} = O_p(1)$, $j=3,4,5$, so are bounded in probability, it is

straightforward by tedious algebra to show that

$$
\begin{aligned}
& n^{1/2}\left\{\widehat{\mu}_{samp}(a_1,a_2) - \mu^*(a_1,a_2)\right\} \\
&= \left\{\frac{1}{\alpha_2^*(a_1)}\right\}\Bigg(n^{1/2}\{\widehat{\alpha}_1(a_1) - \alpha_1^*(a_1)\} - \mu^*(a_1,a_2)\, n^{1/2}\{\widehat{\alpha}_2(a_1) - \alpha_2^*(a_1)\} \\
&\quad + \left\{\frac{\alpha_3^*(a_1,a_2) + \alpha_5^*(a_1,a_2)}{\alpha_4^*(a_1,a_2)}\right\} \\
&\qquad \times \left[-\left\{\frac{\alpha_6^*(a_1)}{\alpha_4^*(a_1,a_2)}\right\} n^{1/2}\{\widehat{\alpha}_4(a_1,a_2) - \alpha_4^*(a_1,a_2)\} + n^{1/2}\{\widehat{\alpha}_6(a_1) - \alpha_6^*(a_1)\}\right] \\
&\quad + \left\{\frac{\alpha_6^*(a_1)}{\alpha_4^*(a_1,a_2)}\right\} n^{1/2}\left[\{\widehat{\alpha}_3(a_1,a_2) + \widehat{\alpha}_5(a_1,a_2)\} - \{\alpha_3^*(a_1,a_2) + \alpha_5^*(a_1,a_2)\}\right] \Bigg) + o_p(1).
\end{aligned} \tag{C8}
$$

Substituting the expressions for $\widehat{\alpha}_1(a_1), \widehat{\alpha}_2(a_1), \widehat{\alpha}_3(a_1,a_2), \widehat{\alpha}_4(a_1,a_2), \widehat{\alpha}_5(a_1,a_2), \widehat{\alpha}_6(a_1)$ in (C8) and simplifying leads to

$$
\begin{aligned}
\mathcal{I}_i &= \left\{\frac{1}{\alpha_2^*(a_1)}\right\}\Bigg(I(A_{1i}=a_1, R_{1i}=1, Y_{1i}=1) - \alpha_1^*(a_1) - \mu^*(a_1,a_2)\{I(A_{1i}=a_1) - \alpha_2^*(a_1)\} \\
&\quad + \left\{\frac{\alpha_3^*(a_1,a_2) + \alpha_5^*(a_1,a_2)}{\alpha_4^*(a_1,a_2)}\right\}\Bigg[-\left\{\frac{\alpha_6^*(a_1)}{\alpha_4^*(a_1,a_2)}\right\} \\
&\quad \times \{I(A_{1i}=a_1, R_{1i}=0, A_{2i}=a_2) - \alpha_4^*(a_1,a_2)\} + \{I(A_{1i}=a_1, R_{1i}=0) - \alpha_6^*(a_1)\}\Bigg] \\
&\quad + \left\{\frac{\alpha_6^*(a_1)}{\alpha_4^*(a_1,a_2)}\right\}[I(A_{1i}=a_1, R_{1i}=0, A_{2i}=a_2, R_{2i}=1, Y_{2i}=1) \\
&\quad + I(A_{1i}=a_1, R_{1i}=0, A_{2i}=a_2, R_{2i}=0, Y_{3i}=1) \\
&\qquad\qquad - \{\alpha_3^*(a_1,a_2) + \alpha_5^*(a_1,a_2\}] \Bigg).
\end{aligned} \tag{C9}
$$

Assuming that $n^{-1}\sum_{i=1}^n \mathcal{I}_i^2$ converges in probability to $\Sigma > 0$, we can conclude that

$$
n^{1/2}\left\{\widehat{\mu}_{samp}(a_1,a_2) - \mu^*(a_1,a_2)\right\}
$$

converges in distribution to a mean-zero normal random variable with variance Σ.

For practical use, letting $\widehat{\mathcal{I}}_i$ denote (C9) with $\widehat{\alpha}_1(a_1), \widehat{\alpha}_2(a_1), \widehat{\alpha}_3(a_1,a_2), \widehat{\alpha}_4(a_1,a_2), \widehat{\alpha}_5(a_1,a_2)$, $\widehat{\alpha}_6(a_1)$ and $\widehat{\mu}_{samp}(a_1,a_2)$ substituted for $\alpha_1^*(a_1), \alpha_2^*(a_1), \alpha_3^*(a_1,a_2), \alpha_4^*(a_1,a_2), \alpha_5^*(a_1,a_2), \alpha_6^*(a_1)$ and $\mu^*(a_1,a_2)$, estimate Σ by $n^{-1}\sum_{i=1}^n \widehat{\mathcal{I}}_i^2$.

Weighted sample proportions estimator. The foregoing result was derived by taking $\mathcal{O}_i$, $i=1,\dots,n$, to be i.i.d. and applying standard theory. Because these data are not i.i.d.

owing to the adaptive randomization, inferences based on this result are likely to be flawed. Accordingly, we now consider derivation of the estimator $\widehat{\mu}_{wtsamp}(a_1, a_2)$, which, as discussed in Section 3.3 of the main paper, involves weighted versions of estimators for each of the probabilities in (C5). The derivation is heuristic and follows the spirit of arguments in Norwood et al. (2024), which adapts the approach of Zhang et al. (2021b) on theory for M-estimators based on adaptively collected data.

Define the set of all potential outcomes for a randomly chosen patient in the subtype population as

$$\mathcal{W}^* = \{R_1^*(a_1), Y_1^*(a_1), R_2^*(a_1, a_2), Y_2^*(a_1, a_2), Y_3^*(a_1, a_2), \text{ for all } (a_1, a_2) \in \mathcal{A}_1 \times \mathcal{A}_2\}.$$

Here, $R_1^*(a_1)$ is the response status and $Y_1^*(a_1)$ is the pCR status a patient would achieve if given stage 1 treatment a_1; $R_2^*(a_1, a_2)$ is the response status a patient would achieve if given stage 1 treatment a_1 followed by stage 2 treatment a_2; and $Y_2^*(a_1, a_2)$ is the pCR status a patient would achieve if given stage 1 treatment a_1 followed by stage 2 treatment a_2 at the end of stage 2, and $Y_3^*(a_1, a_2)$ is the pCR status at the end of stage 3. Although the observed data $\mathcal{O}_i$, $i = 1, \ldots, n$, are not i.i.d., the potential outcomes $\mathcal{W}_i^*$, $i = 1, \ldots, n$, are i.i.d. We make standard identifiability assumptions, which are discussed extensively elsewhere (Tsiatis et al., 2020): (i) consistency, which implies that observed variables are equal to their potential counterparts under the treatments actually received; (ii) positivity, which implies that the probabilities of being assigned any treatment in $\mathcal{A}_1$ and $\mathcal{A}_2$ are strictly greater than zero, which holds in ISPY2 through the use of "clipping constants" that prevent adaptive probabilities from dropping below a set threshold; and (iii) sequential randomization, which holds by design in a nonadaptively randomized SMART and is discussed further below in the case the adaptively randomized ISPY2 SMART.

From (C5), we can write

$$\mu(a_1, a_2) = \phi_1(a_1) + \phi_2(a_1)\{\phi_3(a_1, a_2) + \phi_4(a_1, a_2)\}, \tag{C10}$$

where

$$\phi_1(a_1) = \frac{\alpha_1(a_1)}{\alpha_2(a_1} = P(R_1 = 1, Y_1 = 1 \mid A_1 = a_1) \tag{C11}$$

$$\phi_2(a_1) = \frac{\alpha_6(a_1)}{\alpha_2(a_1)} = P(R_1 = 0 \mid A_1 = a_1) \tag{C12}$$

$$\phi_3(a_1, a_2) = \frac{\alpha_3(a_1, a_2)}{\alpha_4(a_1, a_2)} = P(R_2 = 1, Y_2 = 1 \mid A_1 = a_1, R_1 = 0, A_2 = a_2) \tag{C13}$$

$$\phi_4(a_1, a_2) = \frac{\alpha_5(a_1, a_2)}{\alpha_4(a_1, a_2)} = P(R_2 = 0, Y_3 = 1 \mid A_1 = a_1, R_1 = 0, A_2 = a_2) \tag{C14}$$

For some set of weights $w_i^{(1)}(a_1)$, $i = 1, \ldots, n$, where we reiterate that i indexes only patients with $S = s$, define

$$\widehat{\alpha}_1^w(a_1) = n^{-1} \sum_{i=1}^n w_i^{(1)}(a_1) I(A_{1i} = a_1, R_{1i} = 1, Y_{1i} = 1), \quad \widehat{\alpha}_2^w(a_1) = n^{-1} \sum_{i=1}^n w_i^{(1)}(a_1) I(A_{1i} = a_1).$$

Then note that the obvious estimator for $\phi_1(a_1)$ in (C11) is $\widehat{\phi}_1(a_1) = \widehat{\alpha}_1^w(a_1)/\widehat{\alpha}_2^w(a_1)$, which solves in $\phi_1(a_1)$ an M-estimating equation of the form

$$\sum_{i=1}^n w_i^{(1)}(a_1) I(A_{1i} = a_1)\{I(R_{1i} = 1, Y_{1i} = 1) - \phi_1(a_1)\} = \sum_{i=1}^n M\{A_{1i}, R_{1i}, Y_{1i}, a_1; \phi_1(a_1)\} = 0. \tag{C15}$$

Following Zhang et al. (2021b) and Norwood et al. (2024), we choose the $w_i^{(1)}(a_1)$ so that the estimator $\widehat{\phi}_1(a_1)$ is consistent for $\phi_1(a_1)$ with asymptotic normal theory that can be established via the martingale central limit theorem. Assume for definiteness that patient i enrolled during the interval $[t, t + 1)$, so was assigned to stage 1 treatment according to randomization probabilities $\pi_{1,t}(a_1 \mid \mathcal{D}_t)$, $a_1 \in \mathcal{A}_1$, where as in the main paper $\mathcal{D}_t$ comprises the data from patients enrolling prior to week t, and we suppress the damping constant for brevity. Application of the martingale central limit theorem requires that the $w_i^{(1)}(a_1)$, which depend only on $\mathcal{D}_t$ and not on data on patient i, are chosen so that (i) the estimating equation (C15) remains conditionally (on $\mathcal{D}_t$) unbiased, $E[M\{A_{1i}, R_{1i}, Y_{1i}, a_1; \phi_1(a_1)\} \mid \mathcal{D}_t] = 0$; and (ii) the variance is stabilized,

$$E[M^2\{A_{1i}, R_{1i}, Y_{1i}, a_1; \phi_1(a_1)\} \mid \mathcal{D}_t] = \sigma^2 > 0,$$

a constant not depending on t. With $w_i^{(1)}(a_1)$ so chosen, it then follows that the large sample distribution of $\widehat{\phi}_1(a_1)$ can approximated as described below.

To show (i), note by the consistency assumption that a summand in (C15) can be written equivalently as

$$w_i^{(1)}(a_1)I(A_{1i}=a_1)[I\{R_{1i}^*(a_1)=1, Y_{1i}^*(a_1)=1\}-\phi_1(a_1)].$$

Note then that

$$\begin{aligned}
&E\left(w_i^{(1)}(a_1)I(A_{1i}=a_1)[I\{R_{1i}^*(a_1)=1, Y_{1i}^*(a_1)=1\}-\phi_1(a_1)] \,\Big|\, \mathcal{D}_t\right)\\
&\quad = E\left\{E\left(w_i^{(1)}(a_1)I(A_{1i}=a_1)[I\{R_{1i}^*(a_1)=1, Y_{1i}^*(a_1)=1\}-\phi_1(a_1)] \,\Big|\, \mathcal{D}_t, \mathcal{W}_i^*\right) \,\Big|\, \mathcal{D}_t\right\}\\
&\quad = E\left(w_i^{(1)}(a_1)[I\{R_{1i}^*(a_1)=1, Y_{1i}^*(a_1)=1\}-\phi_1(a_1)]\, E\{I(A_{1i}=a_1) \mid \mathcal{D}_t, \mathcal{W}_i^*\} \,\Big|\, \mathcal{D}_t\right)\\
&\quad = w_i^{(1)}(a_1)\pi_{1,t}(a_1 \mid \mathcal{D}_t)\, E\left([I\{R_{1i}^*(a_1)=1, Y_{1i}^*(a_1)=1\}-\phi_1(a_1)] \mid \mathcal{D}_t\right) \qquad \text{(C16)}\\
&\quad = w_i^{(1)}(a_1)\pi_{1,t}(a_1 \mid \mathcal{D}_t)\, E([I\{R_{1i}^*(a_1)=1, Y_{1i}^*(a_1)=1\}-\phi_1(a_1)]=0. \qquad \text{(C17)}
\end{aligned}$$

Here, (C16) follows by showing that $E\{I(A_{1i}=a_1) \mid \mathcal{D}_t, \mathcal{W}_i^*\}=\pi_{1,t}(a_1 \mid \mathcal{D}_t)$ by an argument conditional on $\mathcal{D}_t$ similar to that in of Tsiatis et al. (2020, Section 6.4.3), noting that i is of the subtype of interest. The first equality in (C17) follows because $\mathcal{D}_t \perp\!\!\!\perp \mathcal{W}_i^*$, where "$\perp\!\!\!\perp$" denotes "independent of" (i.e., the data from past patients are independent of the potential outcomes for patient i); and the second equality follows because in the ISPY2 SMART randomization at the first stage guarantees that $A_{1i} \perp\!\!\!\perp \mathcal{W}_i^*$ given that i is of this subtype (as in the sequential randomization assumption). Thus, using the consistency assumption,

$$\begin{aligned}
P\{R_{1i}^*(a_1)=1, &Y_{1i}^*(a_1)=1\} = P\{R_{1i}^*(a_1)=1, Y_{1i}^*(a_1)=1 \mid A_{1i}=a_1\}\\
&= P(R_{1i}=1, Y_{1i}=1 \mid A_{1i}=1) = P(R_1=1, Y_1=1 \mid A_1=a_1)=\phi_1(a_1),
\end{aligned}$$

given that i is from this subtype. Thus (i) holds for any $w_i^{(1)}(a_1)$ depending only on $\mathcal{D}_t$. To determine $w_i^{(1)}(a_1)$ depending on $\mathcal{D}_t$ such that (ii) is satisfied, note that, similarly to the

above, and using $\{I(A_{1i}=a_1)\}^2 = I(A_{1i}=a_1)$,

$$\begin{aligned}
&E\left(\{w_i^{(1)}(a_1)\}^2 I(A_{1i}=a_1)[I\{R_{1i}^*(a_1)=1, Y_{1i}^*(a_1)=1\} - \phi_1(a_1)]^2 \,\Big|\, \mathcal{D}_t\right) \\
&\quad = E\left(\{w_i^{(1)}(a_1)\}^2 [I\{R_{1i}^*(a_1)=1, Y_{1i}^*(a_1)=\} - \phi_1(a_1)]^2 \, E\{I(A_{1i}=a_1) \mid \mathcal{D}_t, \mathcal{W}_i^*\} \,\Big|\, \mathcal{D}_t\right) \\
&\quad = \{w_i^{(1)}(a_1)\}^2 \pi_{1,t}(a_1 \mid \mathcal{D}_t)\, E\left([I\{(R_{1i}^*(a_1)=1, Y_{1i}^*(a_1)=1\} - \phi_1(a_1)]^2 \mid \mathcal{D}_t\right) \\
&\quad = \{w_i^{(1)}(a_1)\}^2 \pi_{1,t}(a_1 \mid \mathcal{D}_t)\, E\left([I\{R_{1i}^*(a_1)=1, Y_{1i}^*(a_1)1\} - \phi_1(a_1)]^2\right), \qquad \text{(C18)}
\end{aligned}$$

where (C18) follows because $\mathcal{D}_t \perp\!\!\!\perp \mathcal{W}_i^*$. Clearly, because the $\mathcal{W}_i^*$ are i.i.d.,

$$E\left([I\{R_{1i}^*(a_1)=1, Y_{1i}^*(a_1)=1\} - \phi_1(a_1)]^2\right)$$

is the same constant for any i (and thus t). Thus, the entire expression in (C18) will be a constant not depending on t if we take

$$w_i^{(1)}(a_1) = 1/\sqrt{\pi_{1,t}(a_1 \mid \mathcal{D}_t)}. \qquad \text{(C19)}$$

An entirely similar argument can be made for estimation of $\phi_2(a_1)$. Namely, letting

$$\widehat{\alpha}_6^w(a_1) = n^{-1}\sum_{i=1}^n w_i^{(1)}(a_1) I(A_{1i}=a_1, R_{1i}=0),$$

the estimator $\widehat{\phi}_2(a_1) = \widehat{\alpha}_6^w(a_1)/\widehat{\alpha}_2^w(a_1)$ for $\phi_2(a_1)$ in (C12) solves in $\phi_2(a_1)$ an M-estimating equation of the form

$$\sum_{i=1}^n w_i^{(1)}(a_1) I(A_{1i}=a_1)\{I(R_{1i}=0-\phi_2(a_1)\} = \sum_{i=1}^n M\{A_{1i}, R_{1i}, a_1; \phi_2(a_1)\} = 0. \qquad \text{(C20)}$$

Arguments analogous to those above show that $w_i^{(1)}(a_1)$ should be chosen as in (C19).

Similar arguments are possible for estimation of $\phi_3(a_1,a_2)$ and $\phi_4(a_1,a_2)$ in (C13) and (C14). For a set of weights $w_i^{(2)}(a_1,a_2)$, $i=1,\ldots,n$, define

$$\widehat{\alpha}_3^w(a_1,a_2) = n^{-1}\sum_{i=1}^n w_i^{(2)}(a_1,a_2) I(A_{1i}=a_1, R_{1i}=0, A_{2i}=a_2, R_{2i}=1, Y_{2i}=1),$$

$$\widehat{\alpha}_4^w(a_1,a_2) = n^{-1}\sum_{i=1}^n w_i^{(2)}(a_1,a_2) I(A_{1i}=a_1, R_{1i}=0, A_{2i}=a_2)$$

$$\widehat{\alpha}_5^w(a_1,a_2) = n^{-1}\sum_{i=1}^n w_i^{(2)}(a_1,a_2) I(A_{1i}=a_1, R_{1i}=0, A_{2i}=a_2, R_{2i}=0, Y_{3i}=1).$$

Then as above, estimators $\widehat{\phi}_3(a_1,a_2) = \widehat{\alpha}_3^w(a_1,a_2)/\widehat{\alpha}_4^w(a_1,a_2)$, $\widehat{\phi}_4(a_1,a_2) = \widehat{\alpha}_5^w(a_1,a_2)/\widehat{\alpha}_4^w(a_1,a_2)$

solve M-estimating equations

$$\sum_{i=1}^{n} w_i^{(2)}(a_1,a_2) I(A_{1i}=a_1, R_{1i}=0, A_{2i}=a_2)\{I(R_{2i}=1, Y_{2i}=1) - \phi_3(a_1,a_2)\} = 0. \quad \text{(C21)}$$

$$\sum_{i=1}^{n} w_i^{(2)}(a_1,a_2) I(A_{1i}=a_1, R_{1i}=0, A_{2i}=a_2)\{I(R_{2i}=0, Y_{3i}=1) - \phi_4(a_1,a_2)\} = 0. \quad \text{(C22)}$$

As above, assume that patient i enrolled during the interval $[u, u+1)$ and then reached stage 2 during the interval $[t, t+1)$, where $u < t$, so was assigned stage 1 treatment according to randomization probabilities $\pi_{1,u}(a_1 \mid \mathcal{D}_u)$, $a_1 \in \mathcal{A}_1$, and stage 2 treatment according to randomization probabilities $\pi_{2,t}(a_2 \mid a_1, \mathcal{D}_t)$, $a_2 \in \mathcal{A}_2$. Note that $\mathcal{D}_u \subset \mathcal{D}_t$. Application of the martingale central limit theorem now requires that the $w_i^{(2)}(a_1,a_2)$, which depend only on $\mathcal{D}_t$ and not data on patient i, are chosen so that (i) the estimating equations (C21) and (C22) are conditionally (on $\mathcal{D}_t$) unbiased, and (ii) the variance is stabilized. It then follows that with $w_i^{(2)}(a_1,a_2)$ so chosen, the estimators $\widehat{\phi}_3(a_1,a_2)$ and $\widehat{\phi}_4(a_1,a_2)$ are consistent for $\phi_3(a_1,a_2)$ and $\phi_4(a_1,a_2)$ with the large sample distributions that can be established via the martingale central limit theorem.

We demonstrate (i) and determine $w_i^{(2)}(a_1,a_2)$ depending on $\mathcal{D}_t$ such that (ii) is satisfied for (C21); the argument for (C22) is entirely similar and leads to the same choice of $w_i^{(2)}(a_1,a_2)$. First, using the consistency assumption, a summand in (C21) can be written as

$$w_i^{(2)}(a_1,a_2) I(A_{1i}=a_1, A_{2i}=a_2) I\{R_{1i}^*(a_1)=0\}[I\{R_{2i}^*(a_1,a_2)=1, Y_{2i}^*(a_1,a_2)=1\} - \phi_3(a_1,a_2)].$$

Then (i) follows because

$$
\begin{aligned}
&E\left(w_i^{(2)}(a_1,a_2)I(A_{1i}=a_1,A_{2i}=a_2)I\{R_{1i}^*(a_1)=0\}[I\{R_{2i}^*(a_1,a_2)=1,Y_{2i}^*(a_1,a_2)=1\}-\phi_3(a_1,a_2)]\,\Big|\,\mathcal{D}_t\right)\\
&\quad=E\Big\{E\Big(w_i^{(2)}(a_1,a_2)I(A_{1i}=a_1,A_{2i}=a_2)I\{R_{1i}^*(a_1)=0\}\\
&\qquad\times\ [I\{R_{2i}^*(a_1,a_2)=1,Y_{2i}^*(a_1,a_2)=1\}-\phi_3(a_1,a_2)]\,\Big|\,\mathcal{D}_t,\mathcal{W}_i^*\Big)\,\Big|\,\mathcal{D}_t\Big\}\\
&\quad=E\Big(w_i^{(2)}(a_1,a_2)I\{R_{1i}^*(a_1)=0\}[I\{R_{2i}^*(a_1,a_2)=1,Y_{2i}^*(a_1,a_2)=1]-\phi_3(a_1,a_2)]\\
&\qquad\times\ E\{I(A_{1i}=a_1,A_{2i}=a_2)\mid\mathcal{D}_t,\mathcal{W}_i^*\}\,\Big|\,\mathcal{D}_t\Big)\\
&\quad=E\Big(w_i^{(2)}(a_1,a_2)\pi_{1,u}(a_1\mid\mathcal{D}_u)\pi_{2,t}(a_2\mid a_1,\mathcal{D}_t)I\{R_{1i}^*(a_1)=0\}\\
&\qquad\times\ [I\{R_{2i}^*(a_1,a_2)=1,Y_{2i}^*(a_1,a_2)=1\}-\phi_3(a_1,a_2)]\,\Big|\,\mathcal{D}_t\Big) &&\text{(C23)}\\
&\quad=w_i^{(2)}(a_1,a_2)\pi_{1,u}(a_1\mid\mathcal{D}_u)\pi_{2,t}(a_2\mid a_1,\mathcal{D}_t)E\big(I\{R_{1i}^*(a_1)=0\}\\
&\qquad\times[I\{R_{2i}^*(a_1,a_2)=1,Y_{2i}^*(a_1,a_2)=1\}-\phi_3(a_1,a_2)]\big)=0. &&\text{(C24)}
\end{aligned}
$$

Here, as above the equality in (C23) follows because $E\{I(A_{1i}=a_1,A_{2i}=a_2)\mid\mathcal{D}_t,\mathcal{W}_i^*\}=\pi_{1,u}(a_1\mid\mathcal{D}_u)\pi_{2,t}(a_2\mid a_1,\mathcal{D}_t)$ by an argument conditional on $\mathcal{D}_t$ similar to that in of Tsiatis et al. (2020, Section 6.4.3). The first equality in (C24) holds because $\mathcal{D}_t \perp\!\!\!\perp \mathcal{W}_i^*$ as above. The second equality in (C24) holds because, using $E[I\{R_{1i}^*(a_1)=0\}]=P\{R_{1i}^*(a_1)=0\}$, the consistency assumption, and the fact that randomization guarantees $A_{1i}\perp\!\!\!\perp\mathcal{W}_i^*$ and $A_{2i}\perp\!\!\!\perp\mathcal{W}_i^*\mid R_{1i}=0,A_{1i}$ given i is also of this subtype (as in the sequential randomization

assumption)

$$
\begin{aligned}
&P\{R^*_{1i}(a_1)=0, R^*_{2i}(a_1,a_2)=1, Y^*_{2i}(a_1,a_2)=1\}\\
&\quad = P\{R^*_{1i}(a_1)=0, R^*_{2i}(a_1,a_2)=1, Y^*_{2i}(a_1,a_2)=1 \mid A_{1i}=a_1\}\\
&\quad = P\{R^*_{2i}(a_1,a_2)=1, Y^*_{2i}(a_1,a_2)=1 \mid A_{1i}=a_1, R^*_{1i}(a_1)=0\}\, P\{R^*_{1i}(a_1)=0 \mid A_{1i}=a_1\}\\
&\quad = P\{R^*_{2i}(a_1,a_2)=1, Y^*_{2i}(a_1,a_2)=1 \mid A_{1i}=a_1, R^*_{1i}(a_1)=0\}\ P\{R^*_{1i}(a_1)=0\}\\
&\quad = P\{R^*_{2i}(a_1,a_2)=1, Y^*_{2i}(a_1,a_2)=1 \mid A_{1i}=a_1, R_{1i}=0\}\ P\{R^*_{1i}(a_1)=0\}\\
&\quad = P\{R^*_{2i}(a_1,a_2)=1, Y^*_{2i}(a_1,a_2)=1 \mid A_{1i}=a_1, R_{1i}=0, A_{2i}=a_2\}\ P\{R^*_{1i}(a_1)=0\}\\
&\quad = P(R_{2i}=1, Y_{2i}=1 \mid A_{1i}=a_1, R_{1i}=0, A_{2i}=a_2) P\{R^*_{1i}(a_1)=0\}\\
&\quad = P(R_2=1, Y_2=1 \mid A_1=a_1, R_1=0, A_2=a_2)\, P\{R^*_{1i}(a_1)=0\} = \phi_3(a_1,a_2) P\{R^*_{1i}(a_1)=0\}.
\end{aligned}
$$

Finally, to determine $w_i^{(2)}(a_1,a_2)$ depending on $\mathcal{D}_t$ such that (ii) is satisfied, by similar arguments,

$$
\begin{aligned}
&E\Big(\{w_i^{(2)}(a_1,a_2)\}^2 I(A_{1i}=a_1, A_{2i}=a_2) I\{R^*_{1i}(a_1)=0\}\\
&\qquad \times [I\{R^*_{2i}(a_1,a_2)=1, Y^*_{2i}(a_1,a_2)=1\} - \phi_3(a_1,a_2)]^2 \,\Big|\, \mathcal{D}_t\Big)\\
&\quad = E\Big\{E\Big(\{w_i^{(2)}(a_1,a_2)\}^2 I(A_{1i}=a_1, A_{2i}=a_2) I\{R^*_{1i}(a_1)=0\}\\
&\qquad \times [I\{R^*_{2i}(a_1,a_2)=1, Y^*_{2i}(a_1,a_2)=1\} - \phi_3(a_1,a_2)]^2 \,\Big|\, \mathcal{D}_t, \mathcal{W}^*\Big) \,\Big|\, \mathcal{D}_t\Big\}\\
&\quad = E\Big(\{w_i^{(2)}(a_1,a_2)\}^2 I\{R^*_{1i}(a_1)=0\}[I\{R^*_{2i}(a_1,a_2)=1, Y^*_{2i}(a_1,a_2)=1\} - \phi_3(a_1,a_2)]^2\\
&\qquad \times E\{I(A_{1i}=a_1, A_{2i}=a_2) \mid \mathcal{D}_t, \mathcal{W}^*_i\} \,\Big|\, \mathcal{D}_t\Big)\\
&\quad = \{w_i^{(2)}(a_1,a_2)\}^2 \pi_{1,u}(a_1 \mid \mathcal{D}_u) \pi_{2,t}(a_2 \mid a_1, \mathcal{D}_t)\\
&\qquad \times E\Big(I\{R^*_{1i}(a_1)=0\}[I\{R^*_{2i}(a_1,a_2)=1, Y^*_{2i}(a_1,a_2)=1\} - \phi_3(a_1,a_2)]^2 \,\Big|\, \mathcal{D}_t\Big)\\
&\quad = \{w_i^{(2)}(a_1,a_2)\}^2 \pi_{1,u}(a_1 \mid \mathcal{D}_u) \pi_{2,t}(a_2 \mid a_1, \mathcal{D}_t)\\
&\qquad \times E\left(I\{R^*_{1i}(a_1)=0\}[I\{R^*_{2i}(a_1,a_2)=1, Y^*_{2i}(a_1,a_2)=1\} - \phi_3(a_1,a_2)]^2\right). \qquad \text{(C25)}
\end{aligned}
$$

Because $\mathcal{W}_i$, $i=1,\dots,n$, are i.i.d., the expectation (C25) is a constant not depending on t.

Thus, the entire expression will be constant and not depending on t if we take

$$w_i^{(2)}(a_1) = 1/\sqrt{\pi_{1,u}(a_1 \mid \mathcal{D}_u)\pi_{2,t}(a_2 \mid a_1, \mathcal{D}_t)}. \tag{C26}$$

Based on these results, the proposed weighted estimator for $\mu(a_1, a_2)$ is given by

$$\begin{aligned}\widehat{\mu}_{wtsamp}(a_1, a_2) &= \widehat{\phi}_1(a_1) + \widehat{\phi}_2(a_1)\{\widehat{\phi}_3(a_1, a_2) + \widehat{\phi}_4(a_1, a_2)\} \\ &= \frac{\widehat{\alpha}_1^w(a_1)}{\widehat{\alpha}_2^w(a_1)} + \frac{\widehat{\alpha}_6^w(a_1)}{\widehat{\alpha}_2^w(a_1)}\left\{\frac{\widehat{\alpha}_3^w(a_1, a_2) + \widehat{\alpha}_5^w(a_1, a_2)}{\widehat{\alpha}_4^w(a_1, a_2)}\right\}.\end{aligned} \tag{C27}$$

Similar to Zhang et al. (2021b) and Norwood et al. (2024), from the above, under regularity conditions and by the martingale central limit theorem, suitably scaled versions of $n^{1/2}\{\widehat{\phi}_1(a_1)-\phi_{1.s}(a_1)\}$, $n^{1/2}\{\widehat{\phi}_2(a_1)-\phi_{3.s}(a_1)\}$ and $n^{1/2}[\{\widehat{\phi}_3(a_1, a_2)+\widehat{\phi}_4(a_1, a_2)\}-\{\phi_3(a_1, a_2)+\phi_4(a_1, a_2)\}$ converge in distribution to standard normal random variables. We can write

$$\begin{aligned}n^{1/2}\{\widehat{\mu}_{wtsamp}(a_1, a_2) - \mu(a_1, a_2)\} &= n^{1/2}\{\widehat{\phi}_1(a_1) - \phi_{1.s}(a_1)\} + \{\phi_3(a_1, a_2) \\ &\quad + \phi_4(a_1, a_2)\}n^{1/2}\{\widehat{\phi}_2(a_1) - \phi_2(a_1)\} \\ &\quad + \phi_2(a_1)n^{1/2}[\{\widehat{\phi}_3(a_1, a_2) + \widehat{\phi}_4(a_1, a_2)\} - \{\phi_3(a_1, a_2) + \phi_4(a_1, a_2)\}] + o_p(1).\end{aligned} \tag{C28}$$

Assuming that the scaling factors converge in probability and are well-behaved, $n^{1/2}\{\widehat{\phi}_1(a_1)-\phi_{1.s}(a_1)\}$, $n^{1/2}\{\widehat{\phi}_2(a_1)-\phi_{3.s}(a_1)\}$ and $n^{1/2}[\{\widehat{\phi}_3(a_1, a_2)+\widehat{\phi}_4(a_1, a_2)\}-\{\phi_3(a_1, a_2)+\phi_4(a_1, a_2)\}]$ each themselves converge in distribution to normal random variables, and thus so does (C28). Via tedious arguments, it can be shown that $n^{1/2}\{\widehat{\mu}_{wtsamp}(a_1, a_2) - \mu(a_1, a_2)\}$ converges in distribution to a mean-zero normal random variable with variance $\Sigma^w > 0$, where Σ^w is the

limit in probability of $n^{-1}\sum_{i=1}^n \mathcal{I}_i^{w\,2}$, and

$$\mathcal{I}_i^w = \left\{\frac{1}{\alpha_2(a_1)}\right\}\Bigg(I(A_{1i}=a_1,R_{1i}=1,Y_{1i}=1)-\alpha_1(a_1)-\mu(a_1,a_2)\{I(A_{1i}=a_1)-\alpha_2(a_1)\}$$
$$+\left\{\frac{\alpha_3(a_1,a_2)+\alpha_5(a_1,a_2)}{\alpha_4(a_1,a_2)}\right\}\Bigg[-\left\{\frac{\alpha_6(a_1)}{\alpha_4(a_1,a_2)}\right\}\{I(A_{1i}=a_1,R_{1i}=0,A_{2i}=a_2)-\alpha_4(a_1,a_2)\}$$
$$+\{I(A_{1i}=a_1,R_{1i}=0)-\alpha_6(a_1)\}\Bigg]$$
$$+\left\{\frac{\alpha_6(a_1)}{\alpha_4(a_1,a_2)}\right\}[I(A_{1i}=a_1,R_{1i}=0,A_{2i}=a_2,R_{2i}=1,Y_{2i}=1)$$
$$+I(A_{1i}=a_1,R_{1i}=0,A_{2i}=a_2,R_{2i}=0,Y_{3i}=1)$$
$$-\{\alpha_3(a_1,a_2)+\alpha_5(a_1,a_2\}]\Bigg). \qquad \text{(C29)}$$

For practical use, letting $\widehat{\mathcal{I}}_i^w$ denote (C29) with $\widehat{\alpha}_1^w(a_1), \widehat{\alpha}_2^w(a_1), \widehat{\alpha}_3^w(a_1,a_2), \widehat{\alpha}_4^w(a_1,a_2), \widehat{\alpha}_5^w(a_1,a_2), \widehat{\alpha}_6^w(a_1)$ and $\widehat{\mu}_{wtsamp}(a_1,a_2)$ substituted for $\alpha_1(a_1), \alpha_2(a_1), \alpha_3(a_1,a_2), \alpha_4(a_1,a_2), \alpha_5(a_1,a_2), \alpha_6(a_1)$ and $\mu(a_1,a_2)$, estimate Σ^w by $n^{-1}\sum_{i=1}^n \widehat{\mathcal{I}}_i^{w\,2}$.

Web Appendix D. Derivation of $\mu(a_1,a_2)$

We present the derivation of the expression in (E1) in Web Appendix E for the true value of regime $\{a_1,a_2\}$, namely,

$$\mu(a_1,a_2)=p_1(a_1)+p_2(a_1,a_2)\{1-p_1(a_1)\}\lambda_{spec}+p_3(a_1,a_2)\{1-p_2(a_1,a_2)\}\{1-p_1(a_1)\}\lambda_{spec}^2. \qquad \text{(D1)}$$

under several assumptions we now state.

As defined in the main paper, the durability assumption states that, if a patient achieves pCR after stage 1, $Y_1=1$, but $R_1=0$, so that the patient proceeds to stage 2, then $Y_2=1$; similarly, if a patient achieves pCR after stage 1 or 2, so that $Y_1=1, Y_2=1$ or $Y_1=0, Y_2=1$, but $R_1=R_2=0$, so that the patient proceeds to stage 3, then $Y_3=1$.

Formally, the durability assumption thus implies that

$$P(Y_2 = 1 \mid A_1 = a_1, Y_1 = 1, R_1 = 0, A_2 = a_2) = 1$$

$$P(Y_3 = 1 \mid A_1 = a_1, Y_1 = 0, R_1 = 0, A_2 = a_2, Y_2 = 1, R_2 = 0) = 1 \tag{D2}$$

$$PY_3 = 1 \mid A_1 = a_1, Y_1 = 1, R_1 = 0, A_2 = a_2, Y_2 = 1, R_2 = 0) = 1,$$

and that the event $(Y_3 = 1, Y_2 = 0, Y_1 = 1)$ and similar events occur with probability zero. The sensitivity and sensitivity of the preRCB algorithm as in the main paper are assumed to be independent of treatment and prior pCR status; that is,

$$\begin{aligned} \lambda_{sens} &= P(R_1 = 1 \mid A_1 = a_1, Y_1 = 1) \\ &= P(R_2 = 1 \mid A_1 = a_1, Y_1 = 0, R_1 = 0, A_2 = a_2, Y_2 = 1) \\ &= P(R_2 = 1 \mid A_1 = a_1, Y_1 = 1, R_1 = 0, A_2 = a_2, Y_2 = 1) = 0.53 \end{aligned} \tag{D3}$$

$$\begin{aligned} \lambda_{spec} &= P(R_1 = 0 \mid A_1 = a_1, Y_1 = 0) \\ &= P(R_2 = 0 \mid A_1 = a_1, Y_1 = 0, R_1 = 0, A_2 = a_2, Y_2 = 0) \\ &= P(R_2 = 0 \mid A_1 = a_1, Y_1 = 1, R_1 = 0, A_2 = a_2, Y_2 = 0) = 0.90 \end{aligned} \tag{D4}$$

for all $(a_1, a_2) \in \mathcal{A}_1 \times \mathcal{A}_2$. Regarding the true pCR rates $p_1(a_1)$, $p_2(a_1, a_2)$, $p_3(a_1, a_2)$ defined in the main paper, we make the following surrogacy assumptions, which are implicit in the generative data process used for the simulations; namely,

$$\begin{aligned} p_2(a_1, a_2) &= P(Y_2 = 1 \mid A_1 = a_1, Y_1 = 0, A_2 = a_2) = P(Y_2 = 1 \mid A_1 = a_1, Y_1 = 0, R_1 = 0, A_2 = a_2) \\ p_3(a_1, a_2) &= P(Y_3 = 1 \mid A_1 = a_1, Y_1 = 0, A_2 = a_2, Y_2 = 0) \\ &= P(Y_3 = 1 \mid A_1 = a_1, Y_1 = 0, R_1 = 0, A_2 = a_2 Y_2 = 0, R_2 = 0), \end{aligned} \tag{D5}$$

for $(a_1, a_2) \in \mathcal{A}_1 \times \mathcal{A}_2$; that is, for any stage 1 and 2 treatments and given that a patient has not yet achieved pCR, the result of preRCB testing has no bearing on a patient's current true pCR status. Finally, because simple and adaptive randomization to stage 2 treatment for a patient for whom $R_1 = 0$ depends only on a patient's stage 1 treatment and, in the

latter case, data from previous participants, and not on the patient's true pCR status Y_1,

$$P(A_2 = a_2 \mid A_1 = a_1, Y_1 = y_1, R_1 = 0) = P(A_2 = a_2 \mid A_1 = a_1, R_1 = 0), \quad y_1 = 0, 1. \quad \text{(D6)}$$

With the definitions in (C1)-(C3), from (C4), as in (6) of the main paper, the true value of regime $\{a_1, a_2\}$ is given by

$$\begin{aligned} \mu(a_1, a_2) &= \theta_1(a_1)\gamma_1(a_1) + \{1 - \theta_1(a_1)\}\theta_2(a_1, a_2)\gamma_2(a_1, a_2) \\ &+ \{1 - \theta_1(a_1)\}\{1 - \theta_2(a_1, a_2)\}\gamma_3(a_1, a_2). \end{aligned} \quad \text{(D7)}$$

To demonstrate (D1) under the foregoing assumptions, we reexpress each term in (D7) in terms of the above quantities.

First, it is straightforward, using (D3) and the definition of $p_1(a_1)$ that the first term on the right hand side of (D7) can be written as

$$\begin{aligned} \theta_1(a_1)\gamma_1(a_1) &= P(Y_1 = 1, R_1 = 1 \mid A_1 = a_1) \\ &= P(R_1 = 1 \mid A_1 = a_1, Y_1 = 1)P((Y_1 = 1 \mid A_1 = a_1) = \lambda_{sens}\, p_1(a_1), \end{aligned} \quad \text{(D8)}$$

and $\{1 - \theta_1(a_1)\} = P(R_1 = 0 \mid A_a = a_1)$. To reexpress the second term in (D7), note that

$$\begin{aligned} &\theta_2(a_1, a_2)\gamma_2(a_1, a_2)\} = P(Y_2 = 1, R_2 = 1 \mid A_1 = a_1, R_1 = 0, A_2 = a_2) \\ &= P(Y_2 = 1, R_2 = 1, Y_1 = 0 \mid A_1 = a_1, R_1 = 0, A_2 = a_2) \\ &+ P(Y_2 = 1, R_2 = 1, Y_1 = 1 \mid A_1 = a_1, R_1 = 0, A_2 = a_2) \\ &= P(R_2 = 1 \mid A_1 = a_1, Y_1 = 0, R_1 = 0, A_2 = a_2, Y_2 = 1)\, P(Y_2 = 1 \mid A_1 = a_1, Y_1 = 0, R_1 = 0, A_2 = a_2) \\ &\quad \times P(Y_1 = 0 \mid A_1 = a_1, R_1 = 0, A_2 = a_2) \\ &\quad + P(R_2 = 1 \mid A_1 = a_1, Y_1 = 1, R_1 = 0, A_2 = a_2, Y_2 = 1)\, P(Y_2 = 1 \mid A_1 = a_1, Y_1 = 1, R_1 = 0, A_2 = a_2) \\ &\quad \times P(Y_1 = 1 \mid A_1 = a_1, R_1 = 0, A_2 = a_2) \\ &= \lambda_{sens}\, p_2(a_1, a_2)P(Y_1 = 0 \mid A_1 = a_1, R_1 = 0, A_2 = a_2) + \lambda_{sens}\, P(Y_1 = 1 \mid A_1 = a_1, R_1 = 0, A_2 = a_2), \end{aligned} \quad \text{(D9)}$$

where the final equality follows from (D2) and (D3). Now it is straightforward that

$$
\begin{aligned}
&P(Y_1 = 0 \mid A_1 = a_1, R_1 = 0, A_2 = a_2) \\
&\quad = \left\{ \frac{P(A_2 = a_2 \mid A_1 = a_1, Y_1 = 0, R_1 = 0)}{P(A_2 = a_2 \mid A_1 = a_1, R_1 = 0)} \right\} \left\{ \frac{P(R_1 = 0 \mid A_1 = a_1, Y_1 = 0) P(Y_1 = 0 \mid A_1 = a_1)}{P(R_1 = 0 \mid A_1 = a_1)} \right\} \\
&\quad = \frac{\lambda_{spec}\{1 - p_1(a_1)\}}{1 - \theta_1(a_1)}
\end{aligned} \tag{D10}
$$

using (D4) and (D6). By an entirely similar argument,

$$
P(Y_1 = 1 \mid A_1 = a_1, R_1 = 0, A_2 = a_2) = \frac{(1 - \lambda_{sens}) p_1(a_1)}{1 - \theta_1(a_1)}. \tag{D11}
$$

Combining these results with (D9) then yields

$$
\{1 - \theta_1(a_1)\}\theta_2(a_1, a_2)\gamma_2(a_1, a_2) = p_2(a_1, a_2)\{1 - p_1(a_1)\}\lambda_{sens}\lambda_{spec} + p_1(a_1)\lambda_{sens}(1 - \lambda_{sens}). \tag{D12}
$$

To reexpress the third term in (D7), note that

$$
\begin{aligned}
\{1-\theta_2(a_1, a_2)\}\gamma_3(a_1, a_2) &= P(Y_3 = 1, R_2 = 0 \mid A_1 = a_1, R_1 = 0, A_2 = a_2) \\
&= P(Y_3 = 1, R_2 = 0, Y_1 = 0, Y_2 = 0 \mid A_1 = a_1, R_1 = 0, A_2 = a_2) \\
&\quad + P(Y_3 = 1, R_2 = 0, Y_1 = 0, Y_2 = 1 \mid A_1 = a_1, R_1 = 0, A_2 = a_2) \\
&\quad + P(Y_3 = 1, R_2 = 0, Y_1 = 1, Y_2 = 0 \mid A_1 = a_1, R_1 = 0, A_2 = a_2) \\
&\quad + P(Y_3 = 1, R_2 = 0, Y_1 = 1, Y_2 = 1 \mid A_1 = a_1, R_1 = 0, A_2 = a_2).
\end{aligned} \tag{D13}
$$

Because the event in the third term on the right hand side of (D13) can never occur, this term is equal to zero. We thus consider each of the remaining terms. Using (D4), (D5), and

(D10), the first term on the right hand side of (D13) can be written as

$$\begin{aligned}
&P(Y_3 = 1, R_2 = 0, Y_1 = 0, Y_2 = 0 \mid A_1 = a_1, R_1 = 0, A_2 = a_2) \\
&\quad = P(Y_3 = 1 \mid A_1 = a_1, Y_1 = 0, R_1 = 0, A_2 = a_2 Y_2 = 0) \\
&\quad\quad \times P(R_2 = 0 \mid A_1 = a_1, Y_1 = 0, R_1 = 0, A_2 = a_2, Y_2 = 0) \\
&\quad\quad \times P(Y_2 = 0 \mid A_1 = a_1, Y_1 = 0, R_1 = 0, A_2 = a_2) P(Y_1 = 0 \mid A_1 = a_1, R_1 = 0, A_2 = a_2) \\
&\quad = p_3(a_1, a_2)\, \lambda_{spec} \{1 - p_2(a_1, a_2)\} \frac{\lambda_{spec}\{1 - p_1(a_1)\}}{1 - \theta_1(a_1)}.
\end{aligned} \tag{D14}$$

Similarly, and also using (D2) and (D3), the second and fourth terms can be written as

$$\begin{aligned}
&P(Y_3 = 1, R_2 = 0, Y_1 = 0, Y_2 = 1 \mid A_1 = a_1, R_1 = 0, A_2 = a_2) \\
&\quad = P(Y_3 = 1 \mid A_1 = a_1, Y_1 = 0, R_1 = 0, A_2 = a_2 Y_2 = 1) \\
&\quad\quad \times P(R_2 = 0 \mid A_1 = a_1, Y_1 = 0, R_1 = 0, A_2 = a_2, Y_2 = 1) \\
&\quad\quad \times P(Y_2 = 1 \mid A_1 = a_1, Y_1 = 0, R_1 = 0, A_2 = a_2)\, P(Y_1 = 0 \mid A_1 = a_1, R_1 = 0, A_2 = a_2) \\
&\quad = \ (1 - \lambda_{sens}) p_2(a_1, a_2) \frac{\lambda_{spec}\{1 - p_1(a_1)\}}{1 - \theta_1(a_1)}
\end{aligned} \tag{D15}$$

and

$$\begin{aligned}
&P(Y_3 = 1, R_2 = 0, Y_1 = 1, Y_2 = 1 \mid A_1 = a_1, R_1 = 0, A_2 = a_2) \\
&\quad = P(Y_3 = 1 \mid A_1 = a_1, Y_1 = 1, R_1 = 0, A_2 = a_2 Y_2 = 1) \\
&\quad\quad \times P(R_2 = 0 \mid A_1 = a_1, Y_1 = 1, R_1 = 0, A_2 = a_2, Y_2 = 1) \\
&\quad\quad \times P(Y_2 = 1 \mid A_1 = a_1, Y_1 = 1, R_1 = 0, A_2 = a_2) P(Y_1 = 1 \mid A_1 = a_1, R_1 = 0, A_2 = a_2) \\
&\quad = \ (1 - \lambda_{sens}) \frac{(1 - \lambda_{sens}) p_1(a_1)}{1 - \theta_1(a_1)}.
\end{aligned} \tag{D16}$$

Substituting (D14) - (D16) in (D13), the third term in (D7) is given by

$$\begin{aligned} &\{1-\theta_1(a_1)\}\{1-\theta_2(a_1,a_2)\}\gamma_3(a_1,a_2) \\ &= p_3(a_1,a_2)\{1-p_2(a_1,a_2)\}\{1-p_1(a_1)\}\lambda_{spec}^2 p_2(a_1,a_2)\{1-p_1(a_1)\}(1-\lambda_{sens})\lambda_{spec} \\ &\quad + p_1(a_1)(1-\lambda_{sens})^2. \end{aligned} \tag{D17}$$

Substituting (D8), (D12), and (D17) in (D7) and noting that

$$p_2(a_1,a_2)\{1-p_1(a_1)\}\lambda_{sens}\lambda_{spec}+p_2(a_1,a_2)\{1-p_1(a_1)\}(1-\lambda_{sens})\lambda_{spec} = p_2(a_1,a_2)\{1-p_1(a_1)\}\lambda_{spec}$$

and $p_1(a_1)\{\lambda_{sens}+\lambda_{sens}(1-\lambda_{sens})+(1-\lambda_{sens})^2\} = p_1(a_1)$ yields the expression for the true value in (D1), as desired.

Web Appendix E. Simulation details and additional results

E.1 Simulation details

We provide full details on the simulation scenarios. As in Section 4 of the main paper, we carried out a suite of simulation studies under a range of scenarios based on the I-SPY2 investigators' expectations for experimental stage 1 agents and past data on best-in-class stage 2 agents and rescue therapy from I-SPY2. For each scenario, we evaluate both in-trial performance and the quality of post-trial inference for SMARTs conducted using simple, uniform randomization at each of stages 1 and 2, denoted as SR; and using several versions of the proposed bRAR strategy with different damping constants ψ_t, denoted as BR(ψ_t), where ψ_t is both time independent and dependent as discussed below. In all scenarios, we imposed clipping constants of 0.05 and 0.95 on the adaptive probabilities.

All generative scenarios involve a subtype of interest for which there are two stage 1/Block A experimental treatment options, $\mathcal{A}_1 = \{0,1\}$, and three stage 2/Block B best-in-class options, $\mathcal{A}_2 = \{0,1,2\}$. For all scenarios, aligned with the investigators' beliefs, achievement of pCR was assumed to be durable over the maximum duration of a patient's participation in the trial. That is, if a patient achieves pCR after stage 1, $Y_1 = 1$, but $R_1 = 0$, so that the

patient proceeds to stage 2, then $Y_2 = 1$. Similarly, if a patient achieves pCR after stage 1 or 2, so that $Y_1 = 1, Y_2 = 1$ or $Y_1 = 0, Y_2 = 1$, but $R_1 = R_2 = 0$, so that the patient proceeds to stage 3, then $Y_3 = 1$.

Based on the investigators' extensive evaluation, the sensitivity and specificity of the preRCB algorithm were taken to be independent of treatment and prior pCR status, with sensitivity $\lambda_{sens} = 0.53$ and specificity $\lambda_{spec} = 0.90$. Taking sensitivity and specificity of the preRCB algorithm to be independent of treatment and prior pCR status is characterized as

$$\lambda_{sens} = P(R_1 = 1 \mid A_1 = a_1, Y_1 = 1) = P(R_2 = 1 \mid A_1 = a_1, Y_1 = 0, R_1 = 0, A_2 = a_2, Y_2 = 1)$$
$$= P(R_2 = 1 \mid A_1 = a_1, Y_1 = 1, R_1 = 0, A_2 = a_2, Y_2 = 1) = 0.53$$

(sensitivity) and

$$\lambda_{spec} = P(R_1 = 0 \mid A_1 = a_1, Y_1 = 0) = P(R_2 = 0 \mid A_1 = a_1, Y_1 = 0, R_1 = 0, A_2 = a_2, Y_2 = 0)$$
$$= P(R_2 = 0 \mid A_1 = a_1, Y_1 = 1, R_1 = 0, A_2 = a_2, Y_2 = 0) = 0.90$$

(specificity) for all $(a_1, a_2) \in \mathcal{A}_1 \times \mathcal{A}_2$, suppressing conditioning on $S = s$ for brevity. These relationships were enforced in all generative scenarios.

As noted in the main paper, patients may refuse to follow the preRCB recommendation (i.e., refuse to proceed to surgery when recommended to do so and insist on surgery when not recommended). Because such noncompliance is not substantial in the I-SPY2 SMART and because there are many ways to specify a noncompliance mechanism that could potentially have different impacts and complicate interpretation of results, it was assumed for simplicity that all patients would follow the preRCB recommendation. As noted in the main paper, if in truth noncompliance were substantial, results could be optimistic. Thus, in all scenarios, response status according to preRCB and proceeding to surgery are aligned. A given scenario involves specification of the true pCR rates $p_1(a_1) = P(Y_1 = 1 \mid A_1 = a_1)$, $a_1 = 0, 1$, following stage 1 treatment, and, given that pCR has not yet been achieved following stage 1 or stage 1 and 2 treatment, $p_2(a_1, a_2) = P(Y_2 = 1 \mid A_1 = a_1, Y_1 = 0, A_2 = a_2)$ and

$p_3(a_1,a_2) = P(Y_3 = 1 \mid A_1 = a_1, Y_1 = 0, A_2 = a_2 Y_2 = 0)$, $(a_1,a_2) \in \mathcal{A}_1 \times \mathcal{A}_2$. Under these conditions, it is shown in Web Appendix D that the true value of regime $\{a_1,a_2\}$ is

$$\begin{aligned}\mu(a_1,a_2) =& p_1(a_1) + p_2(a_1,a_2)\{1-p_1(a_1)\}\lambda_{spec} \\ &+ p_3(a_1,a_2)\{1-p_2(a_1,a_2)\}\{1-p_1(a_1)\}\lambda_{spec}^2,\end{aligned} \tag{E1}$$

which depends on the specificity but not on the sensitivity of the preRCB assessment.

Under these specifications, the simulation study for each scenario involved 5000 Monte Carlo trials conducted under each randomization scheme. For each trial, n patients of the subtype were enrolled during an enrollment period of $T_{enroll} = 130$ weeks (2.5 years), where, for simplicity, the enrollment dates for the n patients were sampled uniformly from the integers in $[1, T_{enroll}]$. For trials using SR, all patients were randomized to the options in $\mathcal{A}_1$ and $\mathcal{A}_2$ using equal probabilities of 1/2 and 1/3, respectively. For trials using RAR, a burn-in period was implemented such that patients enrolling through week t_{burn}, where t_{burn} is the week at which the 20th patient enrolled, were randomized using SR at stages 1 and 2. Randomization probabilities were then updated at each week $t = t_{burn}+1, \ldots, T_{end}$, where T_{end} is the last week at which randomization was required, and used to randomize patients enrolling during $[t, t+1)$ for $t \geqslant t_{burn}+1$. RAR was implemented with $\psi_t = 0.25, 0.50, 0.75, 1.00, 0.50(t/T_{end})$, and (t/T_{end}); the last two choices of ψ_t allow adaptation to become more aggressive over time (Thall and Wathen, 2007). For all adaptive randomization schemes, we took $M = 1000$ after preliminary investigation suggesting that this choice is sufficient to achieve stable results that are qualitatively similar to those using larger M while leading to manageable run times.

Under all randomization schemes, for each patient, at enrollment, $A_1 \in \mathcal{A}_1$ was generated as Bernoulli using the current stage 1 randomization probabilities. At week 12 post enrollment, Y_1 was generated as Bernoulli$\{p_1(a_1)\}$ for $A_1 = a_1$, and R_1 was generated as Bernoulli$\{p_R(Y_1)\}$, where $p_R(y) = \lambda_{sens} I(y=1) + (1-\lambda_{spec}) I(y=0)$. If $R_1 = 1$, Y_1 was recorded at week 13 post enrollment, and no further data were generated for the patient.

If $R_1 = 0$, $A_2 \in \mathcal{A}_2$ was generated as trinomial using the current stage 2 randomization probabilities, and at week 25 post enrollment, Y_2 was either generated as Bernoulli$\{p_2(a_1, a_2)\}$ for $A_1 = a_1, A_2 = a_2$ if $Y_1 = 0$ or set equal to Y_1 if $Y_1 = 1$, and R_2 was generated as Bernoulli$\{p_R(Y_2)\}$. If $R_2 = 1$, Y_2 was recorded at week 26 post enrollment, and no further data were generated for the patient. If $R_2 = 0$, at week 38 post enrollment, Y_3 was either generated as Bernoulli$\{p_3(a_1, a_2)\}$ for $A_1 = a_1, A_2 = a_2$ if $Y_2 = 0$ or set equal to Y_2 if $Y_2 = 1$. Thus, $T_{end} = 143$ weeks.

E.2 Additional simulation results

Post-trial inference results, $n = 200$. Table 1 presents the same simulation results as in Table 3 of the main paper for Scenarios 2 and 4 with $n = 200$. In both tables, for each scenario, the Monte Carlo averages of estimates of $\mu(a_1^{opt}, a_2^{opt})$ obtained using $\widehat{\mu}_{Bayes}(a_1, a_2)$, $\widehat{\mu}_{samp}(a_1, a_2)$, and $\widehat{\mu}_{wtsamp}(a_1, a_2)$ are presented; in all cases, the Bayesian estimator $\widehat{\mu}_{Bayes}(a_1, a_2)$ was calculated using $M = 1000$. Also shown for each estimator are Monte Carlo coverage of a 95% Wald confidence interval for $\mu(a_1^{opt}, a_2^{opt})$, average interval length, and the proportion of trials in which the estimator correctly identifies the optimal regime. For $\widehat{\mu}_{samp}(a_1, a_2)$ and $\widehat{\mu}_{wtsamp}(a_1, a_2)$, Monte Carlo efficiency relative to $\widehat{\mu}_{Bayes}(a_1, a_2)$, defined as the Monte Carlo mean square error for $\widehat{\mu}_{Bayes}(a_1, a_2)$ divided by that for the given estimator, is presented.

From the table, the results are all qualitatively similar to those in the main paper.

[Table 1 about here.]

As noted in the main paper and Web Appendix A, for definiteness and brevity, we have limited presentation of evaluation of the quality of estimation of a chosen, specific regime to that for the true optimal regime(s). We have carried out the same evaluation for each of the other embedded regimes under each scenario, and the results are qualitatively similar and are not tabulated in detail here. Qualitative conclusions are that the bias of estimators for the other (suboptimal) embedded regimes is worse that for those for the true optimal

regime reported here and in the main paper. This feature may not be unexpected, as fewer patients have treatment experience consistent with these regimes under bRAR, which is a consequence of the fact the randomization probabilities over time at each stage favor the treatment options that are associated with the optimal regime. Thus, there is less information available on such regimes, and those data are not i.i.d.

Under bRAR, as noted in Section 3.3 of the main paper, estimators for the value of a specific regime may be biased even for large sample sizes because, under RAR, the data from different patients are no longer independent and identically distributed, which is an assumption underlying the estimator $\widehat{\mu}_{samp}(a_1, a_2)$ and a concern for the Bayesian estimator $\widehat{\mu}_{Bayes}(a_1, a_2)$. This phenomenon has been discussed in the literature on bandits and more generally adaptively collected data (e.g., Zhang et al., 2021a,b). Our estimator $\widehat{\mu}_{wtsamp}(a_1, a_2)$ was motivated by the approach of Zhang et al. (2021b) to eliminate this bias by introducing judiciously chosen weights, as shown in detail in Web Appendix C. Figure 1 presents histograms of the 5000 estimates of $\mu(a_1^{opt}, a_2^{opt})$ in Scenario 3 with BR(1) RAR using all three estimators, centered by the true value and scaled by standard error; ideally, these quantities should be approximately standard normal. Consistent with the findings of Zhang et al. (2021b) and Norwood et al. (2024) and with the bias noted above, the distributions of $\widehat{\mu}_{Bayes}(a_1, a_2)$ and $\widehat{\mu}_{samp}(a_1, a_2)$ are centered below zero, the latter reflecting the failure of standard asymptotic theory under RAR. The histogram for $\widehat{\mu}_{wtsamp}(a_1, a_2)$ is centered at zero, confirming that asymptotic normality is achieved through the weighting. Fortunately, the effects of this behavior on confidence interval performance for the three estimators are unremarkable.

[Figure 1 about here.]

Relative efficiency of estimators under bRAR to SR. Table 2 shows Monte Carlo relative efficiency of the indicated estimator under each RAR scheme to the estimator under SR;

because the natural frequentist estimator under SR is $\widehat{\mu}_{samp}(a_1, a_2)$, the efficiency shown for $\widehat{\mu}_{wtsamp}(a_1, a_2)$ under adaptive randomization is relative to $\widehat{\mu}_{samp}(a_1, a_2)$ under SR. Notably, the weighted estimator under adaptive randomization is relatively more efficient in most cases, with mild to moderate efficiency loss only under the most aggressive schemes. Efficiency of $\widehat{\mu}_{samp}(a_1, a_2)$ under adaptive randomization relative to that under SR shows a similar but less dramatic pattern. The Bayesian estimator $\widehat{\mu}_{Bayes}(a_1, a_2)$ shows only modest gains or losses under less aggressive adaptation and greater losses than the other two estimators under more aggressive adaptation, possibly a consequence of its larger bias. Overall, the results suggest that all estimators perform well under less aggressive schemes, with $\widehat{\mu}_{wtsamp}(a_1, a_2)$ gaining efficiency relative to estimation under SR under these conditions, with no or only modest losses under scenarios involving delayed effects.

[Table 2 about here.]

"Null" Scenario 0 post-trial results, $n = 200$. Because it is well known (Norwood et al., 2024; Zhang et al., 2021a,b) that post-trial inference after adaptive randomization using frequentist techniques is particularly challenging when (in the present context) there is no unique optimal embedded regime, we consider a scenario reflecting the most extreme case where all regimes achieve the same pCR rate, to which we refer as the null scenario, Scenario 0; see Table 1 of the main paper. In-trial results for Scenario 0 are presented in Table 2 of the main paper. Post-trial inference results for Scenario 0 are presented in Table 1; for definiteness, we arbitrarily take regime $\{0, 0\}$ to be the optimal regime in calculation of all performance measures.

All estimators exhibit downward bias under all randomization schemes, as for the scenarios reported in the main paper, with the smallest bias occurring for $\widehat{\mu}_{samp}(a_1, a_2)$ under SR, where standard asymptotic theory holds, and $\widehat{\mu}_{wtsamp}(a_1, a_2)$ under adaptive randomization, as for the scenarios in the main paper. Confidence intervals based on the frequentist estimators

$\widehat{\mu}_{samp}(a_1, a_2)$ and $\widehat{\mu}_{wtsamp}(a_1, a_2)$ exhibit coverage below the nominal level in most cases, while those based on $\widehat{\mu}_{Bayes}(a_1, a_2)$ achieve the nominal level; intervals based on this estimator are also shorter $> 98\%$ of the time (not shown). Interestingly, in contrast to the results in the more realistic scenarios in the main paper, the frequentist estimators are relatively much less efficient than $\widehat{\mu}_{Bayes}(a_1, a_2)$. Finally, Table 2 shows that under Scenario 0 all estimators can exhibit notable efficiency loss under adaptive randomization relative to SR, with $\widehat{\mu}_{Bayes}(a_1, a_2)$, although showing some loss, faring considerably better than the other estimators. Overall, these results suggest that one base post-trial inference on this estimator in settings where all regimes achieve similar values.

Scenario 3 results, $n = 120$ and $n = 1000$. Table 3 presents in-trial results for Scenario 3 under the smaller sample size $n = 120$, which would be considered "small" in practice for a SMART involving six embedded regimes, and $n = 1000$, chosen as a sample size that, while possibly larger than resources might allow in some settings, represents a situation where large sample theory would be likely provide good approximations to finite-sample performance. Not surprisingly, for $n = 120$, gains in overall pCR rate under RAR are negligible, as are increases in the proportions of patients consistent with the optimal regime. Randomization probabilities under RAR do increase with more aggressive adaptation, particularly for stage 1, but apparently not sufficiently to produce gains in the previous measures. In contrast, for $n = 1000$, gains of up to 5% in the overall pCR rate are obtained under RAR, with roughly 50% of patients consistent with the optimal regime under more aggressive versions. This feature is undoubtedly a consequence of the very high randomization probabilities under RAR for the stage 1 and 2 treatments associated with the optimal regime. Evidently, with the rich information from this large sample, the RAR methods are able to identify definitively the optimal regime and assign patients to it.

Table 4 shows post-trial results. With $n = 120$, performance is degraded for the Bayesian

estimator, which is very downward biased, whereas the bias is much more modest under $n = 1000$, demonstrating that this bias is in part a sample size issue. Likewise, the downward bias of the weighted estimator is mile for $n_2 = 120$ and almost eliminated entirely with $n = 1000$, again demonstrating that this bias is tied to sample size. Coverage probabilities mostly fall short of the nominal 0.95 level for $n = 120$, reflecting the considerable uncertainty; for $n = 1000$, coverage achieves the nominal level. The length of intervals based on the Bayesian estimator is shorter than the others 85-90% of the time for $n = 120$ and about 80% of the time for $n_2 = 1000$ (not shown). The estimators $\widehat{\mu}_{Bayes}(a_1, a_2)$ and $\widehat{\mu}_{wtsamp}(a_1, a_2)$ estimators are mostly equivalent in terms of efficiency under RAR with both sample sizes.

Table 5 shows that efficiency relative to SR is either the same or worse under $n = 120$ for all RAR methods. With the large sample size $n = 1000$, considerable gains in efficiency over SR under RAR are possible with all estimators.

[Table 3 about here.]

[Table 4 about here.]

[Table 5 about here.]

Temporal effects scenario results, $n = 200$. As noted in the main paper, we considered a scenario under which there is a temporal trend with $n = 200$. Specifically, we took the underlying pCR rates

$$p_1(a_1) = P(Y_1 = 1 \mid A_1 = a_1), \quad a_1 = 0, 1$$

$$p_2(a_1, a_2) = P(Y_2 = 1 \mid A_1 = a_1, Y_1 = 0, A_2 = a_2), \quad (a_1, a_2) \in \mathcal{A}_1 \times \mathcal{A}_2$$

$$p_3(a_1, a_2) = P(Y_3 = 1 \mid A_1 = a_1, Y_1 = 0, A_2 = a_2 Y_2 = 0), \quad (a_1, a_2) \in \mathcal{A}_1 \times \mathcal{A}_2$$

to change over time, which implies from (E1) of the main paper that, for any regime $\{a_1, a_2\}$, the true value $\mu(a_1, a_2)$ changes over time. The scenario is a modified version of antagonistic Scenario 3 in the main paper. Namely, with $\text{expit}(u) = e^u/(1 + e^u)$, for a participant who

enrolled in the trial at time $E \in (0, T_{end})$, where as in all simulations E is distributed as uniform on 0 to T_{end}, with mean $m_E = T_{end}/2$, we took, now letting the pCR rates depend on E,

$$p_1(a_1, E) = \text{expit}\{\eta_{10} + \eta_{11}(E - m_E)\},$$

where $\eta_{11} = 0.005$ for $a_1 = 0, 1$ and $\text{expit}(\eta_{10}) = 0.30$ for $a_1 = 0$ and $\text{expit}(\eta_{10}) = 0.40$ for $a_1 = 1$. Likewise, we took

$$p_2(a_1, a_2, E) = \text{expit}\{\eta_{20} + \eta_{21}(E - m_E)\},$$

where $\eta_{21} = 0.005$ for all $(a_1, a_2) = (0,0), (0,1), (0,2), (1,0), (1,1), (1,2)$ and $\text{expit}(\eta_{20}) = 0.60, 0.50, 0.30, 0.18, 0.15, 0.10$ for $(a_1, a_2) = (0,0), (0,1), (0,2), (1,0), (1,1), (1,2)$, respectively. We continued to take $p_3(a_1, a_2) = 0.15$, $(a_1, a_2) = (0,0), (0,1), (0,2), (1,0), (1,1), (1,2)$, as in all other scenarios. Under these conditions, it is straightforward to calculate exactly the true pCR rate (value) for each of the six embedded regimes, yielding $\mu(a_1, a_2) = 0.711, 0.657, 0550, 0.558, 0.544, 0.521$ for $(a_1, a_2) = (0,0), (0,1), (0,2), (1,0), (1,1), (1,2)$, and thus regimes $\{0,0\}, \{0,1\}, \{0,2\}\{1,0\}, \{1,1\}, \{1,2\}$, respectively.

Table 6 shows in-trial results. The overall pCR rate increases modestly under bRAR relative to SR; the proportions of patients consistent with the optimal and worst-performing regimes and the final randomization probabilities show similar patterns to those under the non-temporal scenarios.

[Table 6 about here.]

Table 7 shows the post-trial results. There is no effect of the temporal trend in pCR rates on the ability to identify the optimal regime under bRAR relative to SR, with the proportion of trials where the optimal regime is correctly identified increasing with the aggressiveness of adaptation. The quality of inference on the value of the optimal regime is degraded relative to scenarios with no temporal trends in that coverage of 95% confidence intervals is considerably lower than the nominal level. Evidently, and not unexpectedly,

temporal trends have an impact on inference, analogous to experience with inference on treatment effects conventional RCTs with adaptive randomization.

It is possible to extend the methodology we have presented in this paper to incorporate discrete baseline covariates that could possibly be used in final analyses to account for temporal effects without significantly increasing the complexity of the methods. Such methodology is beyond the scope of the current paper but will be fully developed and implemented for post-trial analyses if there is strong evidence of temporal trends in, for example, the patient population over the course of the trial.

[Table 7 about here.]

E.3 "Graduation" of regimes

As noted in the main paper and discussed in Khoury et al. (2024) and Shatsky et al. (2024), the I-SPY2 trial program is focused on signal finding; i.e., identifying treatments that are likely to be successful in phase 3 trials. The reconfiguration to a SMART enables not only evaluation of novel investigational neoadjuvant agents on this basis but also of entire treatment regimes, and agents and regimes that have sufficiently high probability of such success are said to "graduate" from the trial. Graduated regimes are highlighted as those that merit further study as recommended strategies for best using new and previously graduated "best-in-class" treatments toward the precision oncology goal of patients achieving pCR while minimizing the likelihood that they receive unneeded therapy and experience associated unnecessary toxicities.

Here, we discuss graduation of regimes in the I-SPY2 SMART and consider two graduation criteria suggested by Khoury et al. (2024) and Shatsky et al. (2024). In both, information on the value of a given subtype-specific regime from the I-SPY2 SMART is compared to what the investigators refer to as a subtype-specific "dynamic control." The dynamic control is a subtype-specific probability distribution constructed from a weighted combination of

posterior distributions of pCR rates based on the data from previously-tested agents in the pre-SMART I-SPY2 trial, including the I-SPY2 historical control of paciltaxel followed by doxorubicin-cyclophosphamide.

In Khoury et al. (2024) and Shatsky et al. (2024), this comparison is as follows. For $a_1 \in \mathcal{A}_1$, $a_2 \in \mathcal{A}_2$, denote the posterior distribution of the value (true pCR rate) $\mu(a_1, a_2)$ of subtype-specific regime $\{a_1, a_2\}$ by $\rho\{\mu(a_1, a_2) \mid \mathcal{D}_{final}\}$, where, as in the main paper, $\mathcal{D}_{final}$ comprises the final data from the trial. Denote the probability distribution of the subtype-specific pCR rate corresponding to the dynamic control as $\rho(\mu_{DC})$. Then, if $X_{\{a_1,a_2\}}$ is a random variable with distribution $\rho\{\mu(a_1, a_2) \mid \mathcal{D}_{final}\}$ and X_{DC} is a random variable with distribution $\rho(\mu_{DC})$, the regime $\{a_1, a_2\}$ is said to graduate from the SMART if

$$\text{pr}(X_{\{a_1,a_2\}} > X_{DC}) > 0.85, \tag{E2}$$

where pr(·) represents roughly posterior probability based on all past data, including $\mathcal{D}_{final}$ at the end of the SMART.

This first graduation criterion can be implemented by approximating the probability in (E2) by

$$M^{-2} \sum_{m=1}^{M} \sum_{m'=1}^{M} I(X^{(m)}_{\{a_1,a_2\}} > X^{(m')}_{DC}), \tag{E3}$$

where $X^{(m)}_{\{a_1,a_2\}}$, $m = 1, \dots, M$, and $X^{(m)}_{DC}$, $m = 1, \dots, M$, are samples of size M from each of the distributions of $X_{\{a_1,a_2\}}$ and X_{DC}, respectively, and comparing the result to 0.85. To obtain random draws from the distribution of $X_{\{a_1,a_2\}}$, i.e., the posterior distribution $\rho\{\mu(a_1, a_2) \mid \mathcal{D}_{final}\}$, we proceed in a manner similar to estimation of $\mu(a_1, a_2)$ by $\widehat{\mu}_{Bayes}(a_1, a_2)$ at the end of the trial for the subtype of interest by the mean of an approximation to the posterior distribution of $\mu(a_1, a_2)$. Namely, we draw a sample of size M from each posterior distribution in (9) of the main paper to obtain $\Theta^{(m)}_1$, $m = 1, \dots, M$, and substitute these in (5) of the main paper to obtain the sample $X^{(m)}_{\{a_1,a_2\}} = \mu^{(m)}(a_1, a_2)$, $m = 1, \dots, M$. The sample from the dynamic control can be obtained in a similar fashion.

In the simulations we report below, as the specific formulation of the dynamic control is complex and proprietary, based on Figure 4 of Khoury et al. (2024) and Figure 3 of Shatsky et al. (2024), we represent the distribution $\rho(\mu_{DC})$ here by a normal distribution.

Under the second graduation criterion, a subtype-specific regime is said to graduate from the SMART if the posterior probability that the value of the regime exceeds a subtype-specific threshold pCR rate is greater than 0.85. That is, writing $\text{pr}(\cdot \mid \mathcal{D}_{final})$ to denote posterior probability under $\rho\{\mu(a_1, a_2) \mid \mathcal{D}_{final}\}$, for subtype-specific fixed threshold th, regime $\{a_1, a_2\}$ graduates from the trial if

$$\text{pr}\{\mu(a_1, a_2) > th \mid \mathcal{D}_{final}\} > 0.85. \tag{E4}$$

A natural choice for the threshold th is the mean of the dynamic control distribution $\rho(\mu_{DC})$. As for (E2), the posterior probability in (E4), can be approximated for given th by drawing a sample of size M from each posterior distribution in (9) of the main paper to obtain $\Theta_1^{(m)}$, $m = 1, \ldots, M$, and substituting these in (5) of the main paper to obtain $X_{\{a_1,a_2\}}^{(m)} = \mu^{(m)}(a_1, a_2)$, $m = 1, \ldots, M$; the posterior probability in (E4) is then approximated as

$$M^{-1} \sum_{m=1}^{M} I\{X_{\{a_1,a_2\}}^{(m)} > th\}. \tag{E5}$$

Subtype-specific regime $\mu(a_1, a_2)$ would then graduate if (E5) exceeds 0.85.

We evaluate the properties of the graduation strategies based on (E2) and (E4) in the context of the simulations in Section 4 of the main paper and previously in this section. For each graduation strategy, for several of the simulation scenarios and each randomization scheme, we first consider the true optimal subtype-specific regime $\{a_1^{opt}, a_2^{opt}\}$ and a "null" situation where the dynamic control distribution, which we take to be normal as described next, has mean $th = \mu(a_1^{opt}, a_2^{opt})$, so is centered at the true value of the regime of interest. Thus, for the graduation strategy based on (E2), the dynamic control, and thus the distribution of X_{DC}, has mean th, and for the graduation strategy based on (E4), the value of the regime of interest does not exceed the threshold; i.e., $\mu(a_1^{opt}, a_2^{opt}) > th$ does not hold. All simulations

reported next are based on 5000 Monte Carlo trials, and the approximations (E3) and (E5) are based on $M = 1000$ draws.

For the graduation strategy based on (E2), we took the probability distribution of the dynamic control to be normal with mean th and standard deviation the same as that of the approximate posterior distribution of $\mu(a_1^{opt}, a_2^{opt})$ (as determined by M samples), so that the distributions of X_{a_1,a_2} and X_{DC} have the same variance; this choice is consistent with the visual evidence in Khoury et al. (2024) and Shatsky et al. (2024) cited above. In Table 8, the entries for each scenario and randomization scheme reported for $\widehat{\mu}_{Bayes}(a_1, a_2)$ are the Monte Carlo average of indicators that (E3) exceeds 0.85, so represent the proportion of trials for which the regime would graduate despite the distributions of $X_{\{a_1,a_2\}}$ and X_{DC} being similar. The results show that, under this null situation for Scenario 3 with $n = 200$, the proportion of trials where the regime would mistakenly be concluded to graduate is very low, about 0.03 under simple, uniform randomization (SR), increasing modestly under bRAR. With smaller sample size $n = 120$, these probabilities are smaller, around 0.02-0.03, and are larger with $n = 1000$, around 0.05. Under the Temporal effects scenario with $n = 200$, which is similar to Scenario 3, the proportions are mostly inflated under bRAR relative to those for Scenario 3 with this same n. The proportions are similar to those for Scenario 3 with $n = 200$ for Scenario 5, with two regimes achieving the largest value/pCR rate, and are somewhat larger for Scenario 0, where all regimes achieve the same pCR rate. Overall, across scenarios, the probability of mistakenly recommending a regime for graduation when its true pCR rate offers no improvement over previous treatments as reflected by the dynamic control is low.

For comparison, we also consider a "frequentist" version of the graduation strategy under which the posterior distribution $\mu(a_1^{opt}, a_2^{opt})$ is approximated by the asymptotic (large n) normal distribution of $\widehat{\mu}_{samp}(a_1, a_2)$ and $\widehat{\mu}_{wtsamp}(a_1, a_2)$, respectively, assuming the true value of the regime of interest and thus mean of this distribution is th. That is, instead of

sampling from the posterior, we obtained M samples from each of these asymptotic normal distributions. (This approach is not used in the I-SPY2 SMART.) From Table 8, across all scenarios, the proportion of trials where the regime would mistakenly be concluded to graduate under this approach for both $\widehat{\mu}_{samp}(a_1, a_2)$ and $\widehat{\mu}_{wtsamp}(a_1, a_2)$ is larger than that based on sampling from the posterior, in many cases considerably so. We conclude that the graduation strategy based on (E2) as implemented by sampling from the posterior as in (E3), is preferred over these frequentist approximations when the regime of interest does not offer improvement over previous treatments as reflected by the dynamic control.

[Table 8 about here.]

In Table 9, we show analogous results to those in Table 8 for the graduation strategy based on (E4). Here, the entries for each scenario and randomization scheme reported for $\widehat{\mu}_{Bayes}(a_1, a_2)$ in Table 9 are the Monte Carlo average of the indicators that (E5) based on M samples exceeds 0.85, so represent the proportion of trials for which the regime would graduate under this criterion despite its value not exceeding th. The results show that, under this null situation for Scenario 3 with $n = 200$, the proportion of trials where the regime would mistakenly be concluded to graduate is low, about 0.07 under simple, uniform randomization (SR), increasing modestly under bRAR. Interestingly, with smaller sample size $n = 120$, these probabilities are smaller, and are larger with $n = 1000$; we discuss this feature further momentarily. Under the Temporal effects scenario with $n = 200$, which is similar to Scenario 3, the proportions are inflated relative to those for Scenario 3 with this same n. The proportions are somewhat larger than those for Scenario 3 with $n = 200$ for Scenario 5, with two regimes achieving the largest value/pCR rate, and for Scenario 0, where all regimes achieve the same pCR rate.

For comparison, we also consider a frequentist graduation criterion in the spirit of (E5) based on $\widehat{\mu}_{samp}(a_1, a_2)$ and $\widehat{\mu}_{wtsamp}(a_1, a_2)$ (that is not used in the I-SPY2 SMART) un-

der which the null hypothesis $H_0 : \mu(a_1^{opt}, a_2^{opt}) \leqslant th$ is tested against the alternative $H_A : \mu(a_1^{opt}, a_2^{opt}) > th$ using a one-sided Wald test at level of significance 0.15 based on each of the estimators $\widehat{\mu}_{samp}(a_1, a_2)$ and $\widehat{\mu}_{wtsamp}(a_1, a_2)$. In Table 9, the entries for each scenario and randomization scheme reported for $\widehat{\mu}_{samp}(a_1, a_2)$ and $\widehat{\mu}_{wtsamp}(a_1, a_2)$ are the Monte Carlo proportion of trials for which H_0 is rejected. As expected, the proportions are mostly in the range of 0.13 to 0.17, so appearing to target 0.15; not unexpectedly, under the Temporal effects scenario, the proportions are inflated. Although this criterion and that based on (E5) are very different, because the posterior distribution of a parameter and asymptotic distribution of an estimator of the parameter should be similar as the sample size n approaches infinity, we would expect the entries to become more similar as n increases, which appears to be the case for Scenario 3 as n increases from 200 to 1000. Under Scenario 3, we also ran a simulation of SR only with $n = 5000$; the proportions of trials where the regime would mistakenly be concluded to graduate based on (E5) and testing H_0 based on $\widehat{\mu}_{samp}(a_1, a_2)$ were 0.126 and 0.148, respectively, supporting this view.

[Table 9 about here.]

To gain insight into the properties of both graduation strategies when the true value of a regime exceeds the threshold th, we considered a second situation in which we instead took $th = 0.50$. For the graduation strategy based on (E2), Table 10 shows representative results under Scenario 3, for which, from Table 1 in the main paper, the true optimal true optimal subtype-specific regime $\{0, 0\}$ $\mu(a_1^{opt}, a_2^{opt})$ has true value 0.712, the second most efficacious regime $\{0, 1\}$ has true value 0.658, and the least efficacious regime $\{1, 2\}$ has true value 0.520. In the table, the entries for $\widehat{\mu}_{Bayes}(a_1, a_2)$ are the Monte Carlo average of the indicators that (E3) based on $M = 1000$ exceeds 0.85, so represent the proportion of trials for which each regime would graduate when the threshold $th = 0.50$. Under all randomization schemes, this strategy for identifying highly efficacious regimes identifies the true optimal

regime $\{0,0\}$ as graduating 87 to 92% of the time and identifies the second highest regime $\{0,1\}$ as graduating about 60 to 75% of the time. The worst regime, with true value 0.52, which barely exceeds th, is identified as graduating only 8 to 14% of the time. Here, the "frequentist" implementation of (E3) leads to slightly higher graduation rates than those based on the sampling from the posterior.

[Table 10 about here.]

Table 11 shows analogous results for the graduation strategy based on (E4); the entries for $\widehat{\mu}_{Bayes}(a_1,a_2)$ are the Monte Carlo average of the indicators that (E5) based on $M=1000$ exceeds 0.85, so represent the proportion of trials for which each regime would graduate when the threshold $th=0.50$. Under all randomization schemes, this strategy for identifying highly efficacious regimes identifies the true optimal regime $\{0,0\}$ as graduating 90 to 95% of the time and identifies the second highest regime $\{0,1\}$ as graduating about 70 to 81% of the time. The worst regime, with true value 0.52, which barely exceeds th, is identified as graduating only 15 to 22% of the time. Overall, the values are modestly larger than those based on (E2) and (E3), which take account of the entire distributions. For the frequentist criteria based on $\widehat{\mu}_{samp}(a_1,a_2)$ and $\widehat{\mu}_{wtsamp}(a_1,a_2)$, the entries in Table 11 for each of the three regimes correspond to the Monte Carlo power of the test of $H_0: \mu(a_1,a_2) \leqslant th$ versus $H_A: \mu(a_1,a_2) > th$ based on each estimator conducted at level 0.15 and are qualitatively similar or slightly larger, particularly when the test is based on $\widehat{\mu}_{wtsamp}(a_1,a_2)$.

[Table 11 about here.]

Overall, the results suggest that both graduation strategies will identify highly efficacious regimes with high probability while erroneously recommending regimes with values no different from previous treatments (as summarized by the dynamic control) with low probability.

References

Almirall, D., Nahum-Shani, I., Sherwood, N. E., and Murphy, S. A. (2014). ntroduction to SMART designs for the development of adaptive interventions: with application to weight loss research. *Translational Behavioral Medicine* **4,** 260–274.

Ertefaie, A., Wu, T., Lynch, K. G., and Nahum-Shani, I. (2015). Identifying a set that contains the best dynamic treatment regimes. *Biostatistics* **17,** 135–148.

Khoury, K., Meisel, J. L., Yau, C., Rugo, H. S., Nanda, R., Davidian, M., and et al. (2024). Datopotamab–deruxtecan in early-stage breast cancer: the sequential multiple assignment randomized I-SPY2.2 phase 2 trial. *Nature Medicine* **30,** 3737–3747.

Norwood, P., Davidian, M., and Laber, E. (2024). Adaptive randomization methods for sequential multiple assignment randomized trials (SMARTs) via Thompson sampling. *Biometrics* **80,** ujae152.

Shatsky, R. A., Trivedi, M. A., Yau, C., Nanda, R., Rugo, H., Davidian, M., and et al. (2024). Datopotamab–deruxtecan plus durvalumab in early-stage breast cancer: the sequential multiple assignment randomized I-SPY2.2 phase 2 trial. *Nature Medicine* **30,** 3728–3736.

Thall, P. and Wathen, K. (2007). Practical Bayesian adaptive randomization in clinical trials. *European Journal of Cancer* **43,** 859–866.

Tsiatis, A. A., Davidian, M., Holloway, S., and Laber, E. (2020). *Dynamic Treatment Regimes: Statistical Methods for Precision Medicine.* Chapman and Hall/CRC Press, Boca Raton, Florida.

Zhang, K. W., Janson, L., and Murphy, S. A. (2021a). Inference for batched bandits. *arXiv preprint arXiv:2002.03217* .

Zhang, K. W., Janson, L., and Murphy, S. A. (2021b). Statistical inference with M-estimators on adaptively collected data. *Advances in Neural Information Processing Systems* **34,** 7460–7471.

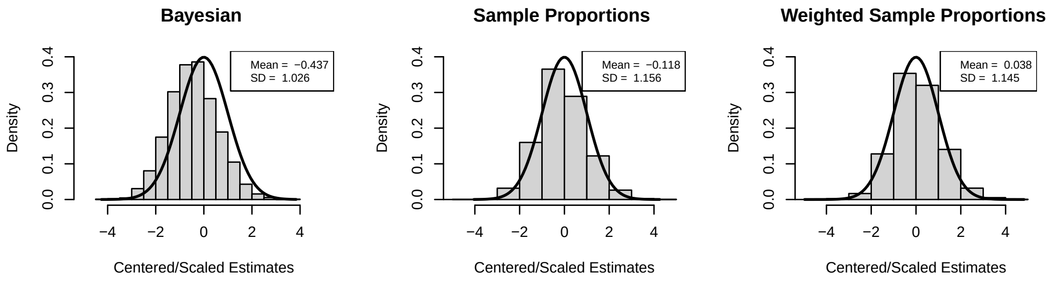

Figure 1. Histograms of 5000 centered and scaled estimates of $\mu(a_1^{opt}, a_2^{opt})$ for Scenario 3 under RAR with $\psi_t = 1$ using the Bayesian estimator $\widehat{\mu}_{Bayes}(a_1, a_2)$, estimator based on sample proportions $\widehat{\mu}_{samp}(a_1, a_2)$, and estimator based on weighted sample proportions $\widehat{\mu}_{wtsamp}(a_1, a_2)$.

Table 1
Post-trial results, $n = 200$, Scenarios 2, 4, and 0. Entries are as in Table 3 of the main paper.

Estimator	Measure	SR	BR(0.25)	BR(0.5)	BR(0.75)	BR(1)	BR($0.5t/T_{end}$)	BR(t/T_{end})
					Scenario 2, $\mu(a_1^{opt}, a_2^{opt}) = 0.712$			
$\widehat{\mu}_{Bayes}(a_1, a_2)$	Prop Est Correct	0.725	0.757	0.769	0.789	0.782	0.756	0.776
	Est pCR Rate Opt Regime	0.685	0.683	0.682	0.681	0.677	0.682	0.680
	Coverage	0.953	0.951	0.938	0.930	0.922	0.946	0.930
	Length	0.266	0.243	0.229	0.220	0.218	0.236	0.223
$\widehat{\mu}_{samp}(a_1, a_2)$	Prop Est Correct	0.723	0.756	0.769	0.789	0.777	0.759	0.773
	Est pCR Rate Opt Regime	0.713	0.704	0.700	0.698	0.692	0.702	0.697
	Coverage	0.923	0.943	0.939	0.938	0.930	0.943	0.936
	Length	0.278	0.253	0.238	0.230	0.228	0.246	0.232
	Rel Efficiency	0.898	1.038	1.060	1.038	1.003	1.056	1.066
$\widehat{\mu}_{wtsamp}(a_1, a_2)$	Prop Est Correct	–	0.756	0.769	0.789	0.777	0.759	0.773
	Est pCR Rate Opt Regime	–	0.708	0.706	0.704	0.701	0.707	0.703
	Coverage	–	0.938	0.939	0.941	0.942	0.939	0.941
	Length	–	0.252	0.239	0.232	0.232	0.246	0.234
	Rel Efficiency	–	1.061	1.139	1.147	1.153	1.105	1.162
					Scenario 4, $\mu(a_1^{opt}, a_2^{opt}) = 0.712$			
$\widehat{\mu}_{Bayes}(a_1, a_2)$	Prop Est Correct	0.695	0.737	0.741	0.769	0.759	0.741	0.756
	Est pCR Rate Opt Regime	0.682	0.682	0.679	0.678	0.675	0.681	0.679
	Coverage	0.946	0.944	0.930	0.932	0.921	0.942	0.932
	Length	0.266	0.244	0.233	0.226	0.226	0.237	0.226
$\widehat{\mu}_{samp}(a_1, a_2)$	Prop Est Correct	0.704	0.743	0.749	0.774	0.765	0.747	0.759
	Est pCR Rate Opt Regime	0.710	0.704	0.698	0.695	0.691	0.701	0.696
	Coverage	0.928	0.941	0.935	0.935	0.933	0.938	0.943
	Length	0.279	0.254	0.243	0.237	0.237	0.247	0.235
	Rel Efficiency	0.931	1.047	1.061	1.032	0.978	1.055	1.088
$\widehat{\mu}_{wtsamp}(a_1, a_2)$	Prop Est Correct	–	0.743	0.749	0.774	0.765	0.745	0.759
	Est pCR Rate Opt Regime	–	0.708	0.703	0.703	0.700	0.706	0.703
	Coverage	–	0.940	0.938	0.937	0.936	0.936	0.946
	Length	–	0.253	0.244	0.239	0.241	0.247	0.237
	Rel Efficiency	–	1.075	1.141	1.161	1.109	1.097	1.202
					Scenario 0, $\mu(a_1^{opt}, a_2^{opt}) = 0.521$			
$\widehat{\mu}_{Bayes}(a_1, a_2)$	Prop Est Correct	0.158	0.169	0.173	0.174	0.169	0.163	0.161
	Est pCR Rate Opt Regime	0.516	0.511	0.504	0.500	0.496	0.508	0.500
	Coverage	0.949	0.954	0.940	0.945	0.950	0.948	0.953
	Length	0.287	0.289	0.292	0.298	0.305	0.290	0.296
$\widehat{\mu}_{samp}(a_1, a_2)$	Prop Est Correct	0.159	0.167	0.173	0.175	0.168	0.165	0.164
	Est pCR Rate Opt Regime	0.518	0.511	0.501	0.495	0.487	0.507	0.500
	Coverage	0.935	0.935	0.917	0.918	0.915	0.929	0.925
	Length	0.310	0.312	0.316	0.324	0.331	0.313	0.321
	Rel Efficiency	0.771	0.746	0.682	0.635	0.593	0.730	0.662
$\widehat{\mu}_{wtsamp}(a_1, a_2)$	Prop Est Correct	–	0.167	0.173	0.175	0.168	0.165	0.164
	Est pCR Rate Opt Regime	–	0.516	0.509	0.507	0.501	0.514	0.506
	Coverage	–	0.938	0.921	0.915	0.904	0.930	0.921
	Length	–	0.314	0.322	0.334	0.331	0.313	0.321
	Rel Efficiency	–	0.761	0.708	0.647	0.590	0.741	0.666

Table 2

Monte Carlo relative efficiency of the estimator for the optimal regime for Scenarios 1 - 5 and 0 under adaptive randomization to the estimator under SR, defined as Monte Carlo mean square error of the estimator under SR divided by that under the indicated adaptive randomization scheme. For $\widehat{\mu}_{wtsamp}(a_1, a_2)$, efficiency is relative to $\widehat{\mu}_{samp}(a_1, a_2)$ under SR.

Scenario	Estimator	BR(0.25)	BR(0.5)	BR(0.75)	BR(1)	BR($0.5t/T_{end}$)	BR(t/T_{end})
1	$\widehat{\mu}_{Bayes}(a_1, a_2)$	0.962	0.868	0.800	0.733	0.930	0.831
	$\widehat{\mu}_{samp}(a_1, a_2)$	1.047	0.953	0.857	0.765	1.017	0.897
	$\widehat{\mu}_{wtsamp}(a_1, a_2)$	1.075	1.020	0.931	0.844	1.051	0.975
2	$\widehat{\mu}_{Bayes}(a_1, a_2)$	1.051	0.997	0.934	0.820	1.034	0.935
	$\widehat{\mu}_{samp}(a_1, a_2)$	1.215	1.178	1.080	0.917	1.216	1.110
	$\widehat{\mu}_{wtsamp}(a_1, a_2)$	1.242	1.266	1.194	1.054	1.272	1.211
3	$\widehat{\mu}_{Bayes}(a_1, a_2)$	0.962	1.011	0.802	0.749	0.973	0.872
	$\widehat{\mu}_{samp}(a_1, a_2)$	1.083	1.184	0.890	0.825	1.128	1.000
	$\widehat{\mu}_{wtsamp}(a_1, a_2)$	1.115	1.266	0.997	0.932	1.184	1.097
4	$\widehat{\mu}_{Bayes}(a_1, a_2)$	1.050	0.941	0.876	0.776	1.035	0.964
	$\widehat{\mu}_{samp}(a_1, a_2)$	1.180	1.072	0.972	0.815	1.173	1.126
	$\widehat{\mu}_{wtsamp}(a_1, a_2)$	1.213	1.153	1.093	0.924	1.220	1.245
5	$\widehat{\mu}_{Bayes}(a_1, a_2)$	0.932	0.824	0.724	0.634	0.879	0.756
	$\widehat{\mu}_{samp}(a_1, a_2)$	1.018	0.903	0.778	0.651	0.992	0.829
	$\widehat{\mu}_{wtsamp}(a_1, a_2)$	1.045	0.964	0.855	0.714	1.032	0.903
0	$\widehat{\mu}_{Bayes}(a_1, a_2)$	0.968	0.827	0.819	0.770	0.944	0.854
	$\widehat{\mu}_{samp}(a_1, a_2)$	0.937	0.731	0.674	0.592	0.894	0.734
	$\widehat{\mu}_{wtsamp}(a_1, a_2)$	0.956	0.760	0.687	0.589	0.907	0.739

Table 3

In-trial results for Scenario 3, $n = 120$ and $n_2 = 1000$. Entries are as in Table 2 of the main paper. Maximum Monte Carlo standard error of entries across randomization schemes followed by the Monte Carlo standard error for SR (for comparison) is given in parentheses for each measure.

Measure	Scenario	SR	BR(0.25)	BR(0.5)	BR(0.75)	BR(1)	BR($0.5t/T_{end}$)	BR(t/T_{end})
					$n = 120$			
Overall pCR Rate (0.0007, 00006)	3	0.590	0.597	0.602	0.607	0.610	0.600	0.606
Consist w/Opt (0.0017, 0.0006)	3	0.244	0.272	0.301	0.321	0.338	0.284	0.316
Consist w/Worst (0.0011, 0.0006)	3	0.257	0.241	0.203	0.212	0.203	0.233	0.217
Rand Prob a_1 Opt (0.0029, –)	3	0.500	0.585	0.660	0.706	0.740	0.656	0.737
Rand Prob a_2 Opt (0.0039, –)	3	0.333	0.407	0.470	0.504	0.537	0.465	0.546
					$n = 1000$			
Overall pCR Rate (0.0003, 0.0002)	3	0.590	0.620	0.636	0.643	0.647	0.625	0.638
Consist w/Opt (0.0015, 0.0002)	3	0.243	0.385	0.475	0.523	0.544	0.416	0.496
Consist w/Worst (0.0004, 0.0002)	3	0.252	0.178	0.142	0.126	0.119	0.168	0.138
Rand Prob a_1 Opt (0.0013, –)	3	0.500	0.836	0.920	0.940	0.946	0.922	0.947
Rand Prob a_2 Opt (0.0027, –)	3	0.333	0.608	0.733	0.801	0.826	0.723	0.824

Table 4

Post-trial results, Scenario 3, $n = 120$ and $n_2 = 1000$. $\mu(a_1^{opt}, a_2^{opt})$ = 0.712 for regime $\{0, 0\}$. Entries are as in Table 3 of the main paper. Maximum Monte Carlo standard errors of Est pCR Rate Opt Regime and Length across randomization schemes are 0.0012 and 0.0011, respectively, for $\widehat{\mu}_{Bayes}(a_1, a_2)$ (0.0010 and 00006 for SR); 0.0015 and 0.0016 for $\widehat{\mu}_{samp}(a_1, a_2)$ (0.0013 and 0.0.0009 for SR); and 0.0015 and 0.0017 for $\widehat{\mu}_{wtsamp}(a_1, a_2)$ for $n = 120$ (0.0013 and – for SR); and 0.0004 and 0.0003 for $\widehat{\mu}_{Bayes}(a_1, a_2)$ (0.0004 and 0.0001 for SR); 0.0005 and 0.0003 for $\widehat{\mu}_{samp}(a_1, a_2)$ (0.0004 and 0.0001 for SR); and 0.0003 and 0.0003 for $\widehat{\mu}_{wtsamp}(a_1, a_2)$ (0.0004 and –) for $n = 1000$. Maximum Monte Carlo standard errors for Coverage and Prop Est Correct across all entries are 0.0038 and 0.0071, respectively.

Estimator	Measure	SR	BR(0.25)	BR(0.5)	BR(0.75)	BR(1)	BR($0.5t/T_{end}$)	BR(t/T_{end})
					$n = 120$			
$\widehat{\mu}_{Bayes}(a_1, a_2)$	Prop Est Correct	0.536	0.556	0.584	0.570	0.579	0.561	0.594
	Est pCR Rate Opt Regime	0.670	0.667	0.665	0.658	0.656	0.665	0.662
	Coverage	0.952	0.940	0.940	0.924	0.924	0.938	0.929
	Length	0.331	0.317	0.306	0.304	0.302	0.312	0.303
$\widehat{\mu}_{samp}(a_1, a_2)$	Prop Est Correct	0.546	0.571	0.595	0.578	0.576	0.569	0.599
	Est pCR Rate Opt Regime	0.712	0.702	0.696	0.684	0.680	0.699	0.690
	Coverage	0.911	0.927	0.931	0.925	0.927	0.930	0.928
	Length	0.355	0.342	0.332	0.333	0.329	0.337	0.328
	Rel Efficiency	0.843	0.932	0.967	1.014	0.860	0.961	0.918
$\widehat{\mu}_{wtsamp}(a_1, a_2)$	Prop Est Correct	–	0.571	0.595	0.578	0.576	0.569	0.599
	Est pCR Rate Opt Regime	–	0.706	0.705	0.695	0.693	0.705	0.700
	Coverage	–	0.924	0.930	0.925	0.922	0.928	0.922
	Length	–	0.339	0.330	0.332	0.330	0.335	0.328
	Rel Efficiency	–	0.957	1.037	1.014	0.953	1.006	0.990
					$n = 1000$			
$\widehat{\mu}_{Bayes}(a_1, a_2)$	Prop Est Correct	0.885	0.926	0.945	0.951	0.942	0.935	0.944
	Est pCR Rate Opt Regime	0.706	0.705	0.706	0.706	0.706	0.705	0.706
	Coverage	0.948	0.942	0.948	0.944	0.938	0.946	0.937
	Length	0.125	0.096	0.086	0.082	0.081	0.092	0.084
$\widehat{\mu}_{samp}(a_1, a_2)$	Prop Est Correct	0.884	0.927	0.946	0.953	0.942	0.936	0.944
	Est pCR Rate Opt Regime	0.712	0.709	0.709	0.709	0.709	0.709	0.709
	Coverage	0.947	0.947	0.948	0.948	0.940	0.950	0.943
	Length	0.126	0.096	0.086	0.082	0.081	0.092	0.084
	Rel Efficiency	0.989	1.067	1.060	1.069	1.065	1.066	1.069
$\widehat{\mu}_{wtsamp}(a_1, a_2)$	Prop Est Correct	–	0.927	0.946	0.953	0.942	0.936	0.944
	Est pCR Rate Opt Regime	–	0.710	0.710	0.710	0.710	0.710	0.710
	Coverage	–	0.948	0.951	0.951	0.952	0.954	0.947
	Length	–	0.098	0.088	0.085	0.084	0.094	0.087
	Rel Efficiency	–	1.065	1.066	1.089	1.121	1.068	1.086

Table 5

Monte Carlo relative efficiency of the estimator for the optimal regime under adaptive randomization to the estimator under SR, defined as in Table 2, Scenario 3, $n = 120$ and $n = 1000$

n	Estimator	BR(0.25)	BR(0.5)	BR(0.75)	BR(1)	BR($0.5t/T_{end}$)	BR(t/T_{end})
120	$\widehat{\mu}_{Bayes}(a_1, a_2)$	0.929	0.889	0.757	0.699	0.905	0.796
	$\widehat{\mu}_{samp}(a_1, a_2)$	1.028	1.019	0.833	0.713	1.032	0.867
	$\widehat{\mu}_{wtsamp}(a_1, a_2)$	1.055	1.094	0.911	0.790	1.080	0.936
1000	$\widehat{\mu}_{Bayes}(a_1, a_2)$	1.461	1.771	1.734	1.558	1.562	1.671
	$\widehat{\mu}_{samp}(a_1, a_2)$	1.577	1.900	1.875	1.678	1.684	1.806
	$\widehat{\mu}_{wtsamp}(a_1, a_2)$	1.574	1.909	1.910	1.766	1.688	1.834

Table 6

In-trial results for temporal effects scenario, $n = 200$. Entries are as in Table 2 of the main paper. Maximum Monte Carlo standard error of entries across randomization schemes followed by the Monte Carlo standard error for SR (for comparison) is given in parentheses for each measure.

Measure	Scenario	SR	BR(0.25)	BR(0.5)	BR(0.75)	BR(1)	BR($0.5t/T_{end}$)	BR(t/T_{end})
Overall pCR Rate (0.0006, 0.0005)	Temporal	0.590	0.600	0.608	0.614	0.619	0.604	0.612
Consist w/Opt (0.0018, 0.0004)	Temporal	0.243	0.289	0.330	0.361	0.384	0.306	0.351
Consist w/Worst (0.0010, 0.0004)	Temporal	0.258	0.229	0.208	0.193	0.181	0.222	0.197
Rand Prob a_1 Opt (0.0025, –)	Temporal	0.500	0.627	0.727	0.790	0.824	0.723	0.826
Rand Prob a_2 Opt (0.0040, —)	Temporal	0.333	0.440	0.525	0.575	0.608	0.517	0.602

Table 7

Post-trial results, $n = 200$, Temporal Scenario. Entries are as in Table 3 of the main paper. Maximum Monte Carlo standard errors of Est pCR Rate Opt Regime and Length across randomization schemes are 0.0011 and 0.0010, respectively, for $\widehat{\mu}_{Bayes}(a_1, a_2)$ (0.0009 and 0.0004 for SR); 0.0012 and 0.0012 for $\widehat{\mu}_{samp}(a_1, a_2)$ (0.0010 and 0.0004 for SR); and 0.0011 and 0.0013 for $\widehat{\mu}_{wtsamp}(a_1, a_2)$ (0.0010 and – for SR). Maximum Monte Carlo standard errors for Coverage and Prop Est Correct across all entries are 0.0038 and 0.0071, respectively.

Estimator	Measure	SR	BR(0.25)	BR(0.5)	BR(0.75)	BR(1)	BR($0.5t/T_{end}$)	BR(t/T_{end})
				Temporal Scenario, $\mu(a_1^{opt}, a_2^{opt}) = 0.711$				
$\widehat{\mu}_{Bayes}(a_1, a_2)$	Prop Est Correct	0.631	0.642	0.683	0.673	0.681	0.669	0.679
	Est pCR Rate Opt Regime	0.684	0.684	0.685	0.684	0.682	0.688	0.685
	Coverage	0.946	0.933	0.923	0.909	0.900	0.930	0.912
	Length	0.267	0.245	0.233	0.227	0.226	0.238	0.228
$\widehat{\mu}_{samp}(a_1, a_2)$	Prop Est Correct	0.640	0.643	0.685	0.677	0.687	0.678	0.682
	Est pCR Rate Opt Regime	0.712	0.707	0.705	0.702	0.698	0.709	0.703
	Coverage	0.923	0.921	0.912	0.904	0.927	0.917	0.905
	Length	0.279	0.256	0.244	0.238	0.238	0.248	0.239
	Rel Efficiency	0.909	0.988	0.998	0.960	0.945	0.981	0.978
$\widehat{\mu}_{wtsamp}(a_1, a_2)$	Prop Est Correct	–	0.643	0.685	0.677	0.687	0.678	0.682
	Est pCR Rate Opt Regime	–	0.708	0.708	0.705	0.703	0.711	0.706
	Coverage	–	0.921	0.923	0.922	0.912	0.924	0.924
	Length	–	0.257	0.246	0.243	0.244	0.250	0.243
	Rel Efficiency	–	1.033	1.113	1.082	1.110	1.048	1.124

Table 8

Graduation strategy based on (E2): Post-trial graduation results under the "null" situation described in the text based on 5000 Monte Carlo trials, where the regime of interest is the true optimal regime. The results for $\widehat{\mu}_{Bayes}(a_1, a_2)$ are the Monte Carlo average of (E3) as described in the text; those for $\widehat{\mu}_{samp}(a_1, a_2)$ and $\widehat{\mu}_{wtsamp}(a_1, a_2)$ are the Monte Carlo average of (E3) where the posterior distribution of the true optimal regime is approximated by the asymptotic distribution of each estimator as described in the text. The maximum Monte Carlo standard error of entries across all scenarios and randomization schemes is 0.0048; the maximum across all scenarios for SR is 0.0046.

Estimator	SR	BR(0.25)	BR(0.5)	BR(0.75)	BR(1)	BR($0.5t/T_{end}$)	BR(t/T_{end})
			Scenario 3, $n = 200$, $th = \mu(a_1^{opt}, a_2^{opt}) = 0.712$				
$\widehat{\mu}_{Bayes}(a_1, a_2)$	0.030	0.036	0.033	0.033	0.036	0.035	0.039
$\widehat{\mu}_{samp}(a_1, a_2)$	0.099	0.086	0.076	0.069	0.075	0.080	0.083
$\widehat{\mu}_{wtsamp}(a_1, a_2)$	–	0.096	0.092	0.086	0.091	0.090	0.097
			Scenario 3, $n = 120$, $th = \mu(a_1^{opt}, a_2^{opt}) = 0.712$				
$\widehat{\mu}_{Bayes}(a_1, a_2)$	0.023	0.025	0.027	0.025	0.027	0.023	0.029
$\widehat{\mu}_{samp}(a_1, a_2)$	0.120	0.095	0.087	0.078	0.079	0.092	0.084
$\widehat{\mu}_{wtsamp}(a_1, a_2)$	–	0.103	0.101	0.094	0.097	0.101	0.102
			Scenario 3, $n = 1000$, $th = \mu(a_1^{opt}, a_2^{opt}) = 0.712$				
$\widehat{\mu}_{Bayes}(a_1, a_2)$	0.051	0.050	0.052	0.053	0.058	0.050	0.054
$\widehat{\mu}_{samp}(a_1, a_2)$	0.080	0.071	0.070	0.072	0.078	0.068	0.068
$\widehat{\mu}_{wtsamp}(a_1, a_2)$	–	0.081	0.076	0.082	0.084	0.076	0.078
			Temporal Scenario, $n = 200$, $th = \mu(a_1^{opt}, a_2^{opt}) = 0.711$				
$\widehat{\mu}_{Bayes}(a_1, a_2)$	0.036	0.051	0.061	0.066	0.073	0.057	0.066
$\widehat{\mu}_{samp}(a_1, a_2)$	0.105	0.115	0.126	0.124	0.132	0.122	0.124
$\widehat{\mu}_{wtsamp}(a_1, a_2)$	–	0.116	0.118	0.118	0.127	0.118	0.118
			Scenario 5, $n = 200$, $th = \mu(a_1^{opt}, a_2^{opt}) = 0.658$				
$\widehat{\mu}_{Bayes}(a_1, a_2)$	0.040	0.039	0.040	0.036	0.037	0.039	0.038
$\widehat{\mu}_{samp}(a_1, a_2)$	0.097	0.087	0.081	0.069	0.068	0.070	0.075
$\widehat{\mu}_{wtsamp}(a_1, a_2)$	–	0.099	0.096	0.091	0.086	0.085	0.090
			Scenario 0, $n = 200$, $th = \mu(a_1^{opt}, a_2^{opt}) = 0.521$				
$\widehat{\mu}_{Bayes}(a_1, a_2)$	0.058	0.058	0.048	0.051	0.046	0.049	0.043
$\widehat{\mu}_{samp}(a_1, a_2)$	0.084	0.077	0.067	0.065	0.057	0.072	0.060
$\widehat{\mu}_{wtsamp}(a_1, a_2)$	–	0.084	0.083	0.085	0.081	0.087	0.076

Table 9

Graduation strategy based on (E4): Post-trial graduation results under the "null" situation described in the text based on 5000 Monte Carlo trials, where the regime of interest is the true optimal regime. . The results for $\widehat{\mu}_{Bayes}(a_1, a_2)$ are the Monte Carlo average of (E5) as described in the text; those for $\widehat{\mu}_{samp}(a_1, a_2)$ and $\widehat{\mu}_{wtsamp}(a_1, a_2)$ are the Monte Carlo proportion of trials for which H_0 is rejected using a one-sided test at level 0.15; see text for details. The maximum Monte Carlo standard error of entries across all scenarios and randomization schemes is 0.0058; the maximum across all scenarios for SR is 0.0056.

Estimator	SR	BR(0.25)	BR(0.5)	BR(0.75)	BR(1)	BR($0.5t/T_{end}$)	BR(t/T_{end})
			Scenario 3, $n = 200$, $th = \mu(a_1^{opt}, a_2^{opt}) = 0.712$				
$\widehat{\mu}_{Bayes}(a_1, a_2)$	0.070	0.072	0.073	0.070	0.078	0.072	0.079
$\widehat{\mu}_{samp}(a_1, a_2)$	0.176	0.160	0.148	0.137	0.144	0.150	0.150
$\widehat{\mu}_{wtsamp}(a_1, a_2)$	–	0.170	0.172	0.160	0.170	0.171	0.177
			Scenario 3, $n = 120$, $th = \mu(a_1^{opt}, a_2^{opt}) = 0.712$				
$\widehat{\mu}_{Bayes}(a_1, a_2)$	0.055	0.052	0.056	0.058	0.062	0.053	0.059
$\widehat{\mu}_{samp}(a_1, a_2)$	0.191	0.169	0.154	0.136	0.142	0.160	0.153
$\widehat{\mu}_{wtsamp}(a_1, a_2)$	–	0.182	0.179	0.169	0.171	0.174	0.181
			Scenario 3, $n = 1000$, $th = \mu(a_1^{opt}, a_2^{opt}) = 0.712$				
$\widehat{\mu}_{Bayes}(a_1, a_2)$	0.110	0.111	0.107	0.102	0.124	0.113	0.111
$\widehat{\mu}_{samp}(a_1, a_2)$	0.154	0.146	0.136	0.146	0.150	0.147	0.138
$\widehat{\mu}_{wtsamp}(a_1, a_2)$	–	0.156	0.149	0.161	0.161	0.163	0.151
			Temporal Scenario, $n = 200$, $th = \mu(a_1^{opt}, a_2^{opt}) = 0.711$				
$\widehat{\mu}_{Bayes}(a_1, a_2)$	0.075	0.097	0.119	0.128	0.133	0.109	0.122
$\widehat{\mu}_{samp}(a_1, a_2)$	0.182	0.190	0.202	0.209	0.217	0.203	0.217
$\widehat{\mu}_{wtsamp}(a_1, a_2)$	–	0.193	0.201	0.202	0.210	0.193	0.201
			Scenario 5, $n = 200$, $th = \mu(a_1^{opt}, a_2^{opt}) = 0.658$				
$\widehat{\mu}_{Bayes}(a_1, a_2)$	0.088	0.090	0.088	0.080	0.081	0.077	0.082
$\widehat{\mu}_{samp}(a_1, a_2)$	0.172	0.158	0.147	0.130	0.129	0.138	0.139
$\widehat{\mu}_{wtsamp}(a_1, a_2)$	–	0.173	0.168	0.163	0.160	0.155	0.169
			Scenario 0, $n = 200$, $th = \mu(a_1^{opt}, a_2^{opt}) = 0.521$				
$\widehat{\mu}_{Bayes}(a_1, a_2)$	0.126	0.121	0.114	0.106	0.097	0.116	0.100
$\widehat{\mu}_{samp}(a_1, a_2)$	0.147	0.141	0.135	0.123	0.116	0.142	0.118
$\widehat{\mu}_{wtsamp}(a_1, a_2)$	–	0.155	0.162	0.158	0.151	0.156	0.150

Table 10

Graduation strategy based on (E2): Post-trial graduation results under the situation where $th = 0.50$ described in the text based on 5000 Monte Carlo trials for the most, second most, and least efficacious regimes in Scenario 3, $n = 200$. Entries are as in Table 8. The maximum Monte Carlo standard error of entries across all randomization schemes is 0.0069; the maximum for SR is 0.0066.

Estimator	Regime	SR	BR(0.25)	BR(0.5)	BR(0.75)	BR(1)	BR($0.5t/T_{end}$)	BR(t/T_{end})
				Scenario 3, $n = 200$, $th = 0.50$				
$\widehat{\mu}_{Bayes}(a_1, a_2)$	{0,0}	0.877	0.877	0.889	0.861	0.847	0.883	0.879
$\widehat{\mu}_{samp}(a_1, a_2)$		0.911	0.906	0.911	0.881	0.865	0.902	0.900
$\widehat{\mu}_{wtsamp}(a_1, a_2)$		–	0.913	0.923	0.899	0.880	0.914	0.911
$\widehat{\mu}_{Bayes}(a_1, a_2)$	{0,1}	0.682	0.685	0.651	0.624	0.601	0.657	0.638
$\widehat{\mu}_{samp}(a_1, a_2)$		0.733	0.731	0.695	0.663	0.644	0.709	0.675
$\widehat{\mu}_{wtsamp}(a_1, a_2)$		–	0.750	0.727	0.703	0.682	0.734	0.706
$\widehat{\mu}_{Bayes}(a_1, a_2)$	{1,2}	0.113	0.096	0.081	0.083	0.078	0.100	0.082
$\widehat{\mu}_{samp}(a_1, a_2)$		0.128	0.112	0.097	0.100	0.094	0.118	0.097
$\widehat{\mu}_{wtsamp}(a_1, a_2)$		–	0.123	0.123	0.132	0.133	0.135	0.120

Table 11

Graduation strategy based on (E4): Post-trial graduation results under the situation where $th = 0.50$ described in the text based on 5000 Monte Carlo trials for the most, second most, and least efficacious regimes in Scenario 3, $n = 200$. Entries are as in Table 9. The maximum Monte Carlo standard error of entries across all randomization schemes is 0.0064; the maximum for SR is 0.0059.

Estimator	Regime	SR	BR(0.25)	BR(0.5)	BR(0.75)	BR(1)	BR($0.5t/T_{end}$)	BR(t/T_{end})
				Scenario 3, $n = 200$, $th = 0.50$				
$\widehat{\mu}_{Bayes}(a_1, a_2)$	{0,0}	0.947	0.935	0.937	0.910	0.890	0.935	0.923
$\widehat{\mu}_{samp}(a_1, a_2)$		0.957	0.943	0.947	0.921	0.902	0.948	0.931
$\widehat{\mu}_{wtsamp}(a_1, a_2)$		–	0.948	0.952	0.932	0.915	0.953	0.941
$\widehat{\mu}_{Bayes}(a_1, a_2)$	{0,1}	0.817	0.806	0.772	0.735	0.708	0.788	0.748
$\widehat{\mu}_{samp}(a_1, a_2)$		0.833	0.822	0.790	0.755	0.726	0.803	0.764
$\widehat{\mu}_{wtsamp}(a_1, a_2)$		–	0.835	0.810	0.781	0.756	0.821	0.789
$\widehat{\mu}_{Bayes}(a_1, a_2)$	{1,2}	0.217	0.192	0.171	0.164	0.157	0.194	0.172
$\widehat{\mu}_{samp}(a_1, a_2)$		0.224	0.191	0.175	0.169	0.162	0.198	0.172
$\widehat{\mu}_{wtsamp}(a_1, a_2)$		–	0.207	0.206	0.208	0.214	0.222	0.208
